# PDGF‐BB‐Dependent Neurogenesis Buffers Depressive‐Like Behaviors by Inhibition of GABAergic Projection from Medial Septum to Dentate Gyrus

**DOI:** 10.1002/advs.202301110

**Published:** 2023-06-16

**Authors:** Hou‐Hong Li, Yang Liu, Hong‐Sheng Chen, Ji Wang, Yu‐Ke Li, Yang Zhao, Rui Sun, Jin‐Gang He, Fang Wang, Jian‐Guo Chen

**Affiliations:** ^1^ Department of Pharmacology School of Basic Medicine Tongji Medical College Huazhong University of Science and Technology Wuhan 430030 China; ^2^ The Key Laboratory for Drug Target Researches and Pharmacodynamic Evaluation of Hubei Province Wuhan 430030 China; ^3^ The Research Center for Depression Tongji Medical College Huazhong University of Science Wuhan 430030 China; ^4^ The Key Laboratory of Neurological Diseases (HUST) Ministry of Education of China Wuhan 430030 China

**Keywords:** depressive‐like behaviors, MS‐DG pathway, neurogenesis, PDGF‐BB

## Abstract

Hippocampal circuitry stimulation is sufficient to regulate adult hippocampal neurogenesis and ameliorate depressive‐like behavior, but its underlying mechanism remains unclear. Here, it is shown that inhibition of medial septum (MS)‐dentate gyrus (DG) circuit reverses the chronic social defeat stress (CSDS)‐induced depression‐like behavior. Further analysis exhibits that inhibition of gamma‐aminobutyric acidergic neurons in MS projecting to the DG (MS^GABA+^‐DG) increases the expression of platelet‐derived growth factor‐BB (PDGF‐BB) in somatostatin (SOM) positive interneurons of DG, which contributes to the antidepressant‐like effects. Overexpression of the PDGF‐BB or exogenous administration of PDGF‐BB in DG rescues the effect of chronic stress on the inhibition of neural stem cells (NSCs) proliferation and dendritic growth of adult‐born hippocampal neurons, as well as on depressive‐like behaviors. Conversely, knockdown of PDGF‐BB facilitates CSDS‐induced deficit of hippocampal neurogenesis and promotes the susceptibility to chronic stress in mice. Finally, conditional knockdown platelet‐derived growth factor receptor beta (PDGFR*β*) in NSCs blocks an increase in NSCs proliferation and the antidepressant effects of PDGF‐BB. These results delineate a previously unidentified PDGF‐BB/PDGFR*β* signaling in regulating depressive‐like behaviors and identify a novel mechanism by which the MS^GABA+^‐DG pathway regulates the expression of PDGF‐BB in SOM‐positive interneurons.

## Graphical abstract

The study describes the functional connectivity in the MS^GABA+^‐DG circuit and the novel downstream molecule PDGF‐BB. Inhibition of MS^GABA+^‐DG pathway increases the expression of PDGF‐BB in SOM‐positive interneurons, which is capable of enhancing adult hippocampal neurogenesis in a PDGFR*β*‐dependent manner by activating the Janus kinase‐signal transducer 2 and activator of transcription 3 (JAK2/STAT3) signaling pathway, leading to the improvement of chronic stress‐induced depressive‐like behavior.

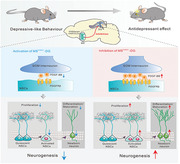

## 1 Introduction

Major depressive disorder (MDD) is the leading cause of disability worldwide, and over 300 million people globally are estimated to suffer from depression, equivalent to 4.4% of the world's population.^[^ ^1^ ^]^ The symptoms of depression are heterogeneous and highly complex, which is attributed to a variety of factors, including genetic predisposition and environmental conditions, such as stress.^[^ ^2^ ^]^ Previous studies have shown that hippocampal atrophy is observed in the patients with depression, including decreased neurogenesis, structural abnormalities, and reduction of hippocampal volume, which is reversed by antidepressant treatments.^[^ ^3^ ^]^ However, the mechanism underpinning the impairment of adult hippocampal neurogenesis in MDD remains elusive.

Previous studies have reported the neuronal circuitry mechanism regulating the dynamic, multi‐stage process of adult neurogenesis, from the activation of quiescent neural stem cells (NSCs) to the survival and synaptic integration of newborn neurons.^[^ ^4^, ^5^ ^]^ Moreover, a study has demonstrated that gamma‐aminobutyric acidergic (GABAergic) projection neurons from the medial septum (MS) send axonal collaterals to parvalbumin (PV)‐expressing interneurons in the dentate gyrus (DG), which is necessary and sufficient to maintain the quiescence of adult NSCs.^[^ ^6^ ^]^ Tozuka et al. found that GABAergic inputs to hippocampal progenitor cells promoted the activity‐dependent neuronal differentiation in the subgranular zone of the adult DG.^[^ ^7^ ^]^ Song et al. showed that adult‐born neurons received local connections from multiple types of GABAergic interneurons, including PV‐positive basket cells, somatostatin (SOM)‐positive cells.^[^ ^8^ ^]^ PV‐expressing interneurons constitute the largest population among diverse GABAergic neurons, which are usually characterized by fast‐spiking profile.^[^ ^9^ ^]^ Optogenetic activation of PV‐expressing interneurons in the DG promotes the survival and development of newborn neurons, and GABA released from PV‐expressing interneurons in the DG serves as a unique local circuit component to maintain the quiescence of adult NSCs and inhibits the symmetrical self‐renewal.^[^ ^10^ ^]^ In addition, activation of cholecystokinin (CCK) interneurons in the DG induces a robust depolarization of NSCs and promotes neurogenic proliferation of NSCs through a dominant astrocyte‐mediated glutamatergic signaling cascade.^[^ ^11^ ^]^ These findings led us to speculate on the role of neuronal circuitry mediated by other GABAergic interneurons in the hippocampal neurogenesis paralleled with behavioral changes in animal model of depression.

The platelet‐derived growth factor (PDGF) family consists of five dimeric PDGF isoforms: PDGF‐AA, AB, BB, CC, DD, which are made up of four PDGF polypeptide chains donated PDGF‐A, ‐B, ‐C, and ‐D. The PDGF isoforms bind to two distinct tyrosine kinase receptors, PDGF receptor alpha (PDGFR*α*), and PDGF receptor beta (PDGFR*β*).^[^ ^12^ ^]^ Emerging evidence shows that PDGF‐mediated signaling regulates diverse functions in the brain, including neurogenesis. Zachrisson et al. found that exogenous administration of PDGF‐BB promoted the proliferation of neural progenitor cells in the subventricular zone in rodent model of Parkinson's disease.^[^ ^13^ ^]^ Meanwhile, a clinical trial reveals that antidepressant treatment elevates the plasma level of PDGF‐BB.^[^ ^14^ ^]^ However, it is unknown whether PDGF‐BB can improve chronic stress‐induced impairment of adult hippocampal neurogenesis and depressive‐like behavior.

Here, we found that chronic inhibition of GABAergic afferents from the MS to the DG ameliorated depressive‐like behaviors through elevating PDGF‐BB expression in SOM‐positive interneuron in the DG. The upregulation of PDGF‐BB level was sufficient to improve stress‐induced deficiency of adult hippocampal neurogenesis and depressive‐like behaviors by activation of PDGFR*β* in the NSCs. Our results identified the MS^GABA+^‐DG circuit as a specific top‐down pathway that mediated the pathological processes of PDGF‐BB deficiency‐induced behavioral abnormality, providing a previously unknown relationship between PDGF‐BB/PDGFR*β* signaling in MS^GABA+^‐DG projection and mood disorders.

## 2 Results

### 2.1 Inhibition of MS‐DG Pathway Ameliorates Depressive‐Like Behaviors of Mice

**Figure 1:**
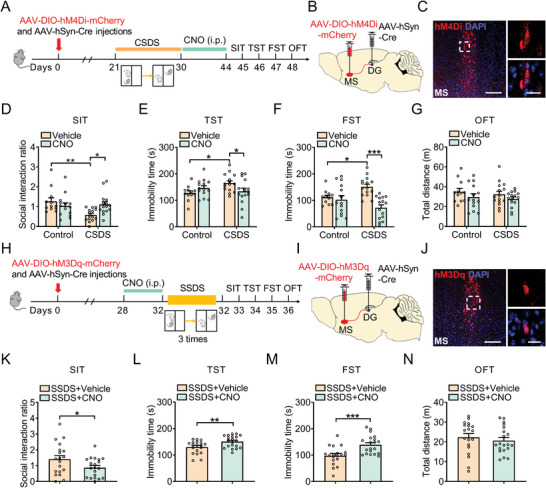
Inhibition of MS‐DG pathway ameliorates depressive‐like behavior of mice. A) Experimental timeline of CSDS protocol and behavioral tests. B) Bilateral delivery of retrograde AAV‐expressing Cre‐recombinase in the DG and AAV carrying Cre‐dependent hM4Di‐mCherry into the MS of C57BL/6J mice. C) mCherry labeling (red) showing hM4Di‐expressing neuron in the DG at low (left) and high (right) magnification. Scale bar: 200 µm (left), 20 µm (right). D) Effects of chemogenetic inhibition of MS‐DG neurons on social interaction ratio in SIT. *n* = 12–15 per group. E) Effects of chemogenetic inhibition of MS‐DG neurons on the immobility time in TST. *n* = 12–15 per group. F) Effects of chemogenetic inhibition of MS‐DG neurons on the immobility time in FST. *n* = 12–15 per group. G) Effects of chemogenetic inhibition of MS‐DG neurons on the locomotor activity in OFT. *n* = 12–15 per group. H) Schematic of SSDS protocol detailing the timing for hM3Dq expression and behavioral tests. I) Bilateral delivery of retrograde AAV expressing Cre‐recombinase in the DG and AAV carrying Cre‐dependent hM3Dq‐mCherry into the MS of C57BL/6J mice. J) mCherry labeling (red) showing hM3Dq‐expressing neuron in the DG at low (left) and high (right) magnification. Scale bar: 200 µm (left), 20 µm (right). K) Effects of chemogenetic activation of MS‐DG neurons on social interaction ratio in SIT. *n* = 19–20 per group. L) Effects of chemogenetic activation of MS‐DG neurons on the immobility time in TST. *n* = 19–20 per group. M) Effects of chemogenetic activation of MS‐DG neurons on the immobility time in FST. *n* = 19–20 per group. N) Effects of chemogenetic inhibition of MS‐DG neurons on the locomotor activity in OFT. *n* = 19–20 per group. Data are expressed as mean ± SEM. Two‐way ANOVA followed by the Bonferroni's post hoc test (D–G), Student's *t‐*test (K–N). *p < 0.05, **p < 0.01, ***p < 0.001. The statistical details can be found in Table S4, Supporting Information.

MS projections to the hippocampal has been demonstrated to participate in the regulation of anxiety‐like behaviors.^[^ ^15^ ^]^ To examine the function of the MS‐DG pathway in stress‐related behavior, we first determined the structural connection between MS and DG with anterograde and retrograde tracing strategies, respectively. Anterograde tracing by injecting AAV‐green fluorescent protein (GFP) into the MS demonstrated that MS neurons sent strong direct efferents into the DG (Figure S1A,B, Supporting Information). Next, we injected the retrograde tracer cholera toxin subunit B (CTB) into the DG of mice and confocal microscopic images showed that retrogradely labeled cells were detected in MS (Figure S1C,D, Supporting Information). To determine whether the projection from the MS to the DG was involved in chronic stress‐induced depression, retrograde adeno‐associated virus expressing Cre‐recombinase driven by hSyn promoter (AAV‐hSyn‐Cre) was injected into the DG, and Cre‐dependent AAV encoding the clozapine‐N‐oxide (CNO)‐activated inhibitory designer receptors exclusively activated by designer drugs (DREADD), hM4Di (AAV‐DIO‐hM4Di‐mCherry),^[^ ^16^ ^]^ was delivered into the MS (**Figure** **1**A–C), resulting in selective expression of hM4Di in DG‐projecting MS neurons. Three weeks after virus infusion, mice received chronic social defeat stress (CSDS) composed of repeated exposure to social defeat stress for 10 min daily for 10 consecutive days.^[^ ^17^ ^]^ It was found that the defeated mice exhibited social avoidance as measured by lower social interaction ratio in the social interaction test (SIT) and reduced sucrose preference in the sucrose preference test (SPT) (Figure S2, Supporting Information). Chemogenetic inhibition of the MS to DG projection resulted in an increase in social interaction ratio of defeated mice compared with that of vehicle‐treated defeated mice (Figure 1D). Furthermore, chemogenetic inhibition reversed the despair behavior of defeated mice, as indicated by the reduction of immobility time in the tail suspension test (TST) and forced swim test (FST) (Figure 1E,F). Silencing of MS to DG projection did not alter the locomotor activity of control and defeated mice (Figure 1G). In addition, chronic inhibition of the MS to DG projection by 5‐day injection of CNO reduced immobility time in the FST and TST in naïve mice expressing hM4Di (Figure S3, Supporting Information), suggesting that inhibition of MS to DG projection produces antidepressant‐like effects. Brain slice electrophysiology recordings validated that firing rate of the MS neurons expressing hM4Di can be silenced by CNO (Figure S4A–C, Supporting Information). The above results demonstrate that chemogenetic silencing of the MS‐DG circuit prevents depressive‐like phenotypes both in healthy and CSDS mice.

Next, to investigate whether chemogenetic activation of the MS to DG projection was sufficient to induce depressive‐like behaviors, the AAV‐DIO‐hM3Dq‐mCherry and a retrograde AAV‐hSyn‐Cre were infused into MS and DG, respectively (Figure 1H–J).^[^ ^18^ ^]^ Whole‐cell patch‐clamp recordings showed that application of CNO produced a significant depolarization of the resting membrane potential, validating this methodological approach (Figure S4D, Supporting Information). Four weeks after virus injection, mice were intraperitoneally administered CNO for 5 days. It was found that activation of MS to DG projection neurons had no effect on the behaviors in naïve mice (Figure S5, Supporting Information). However, when combined with subthreshold social defeat stress (SSDS), which is usually applied for evaluation of susceptibility, activation of MS‐DG projection by CNO could produce depressive‐like phenotypes in multiple tests. In SIT, CNO treatment decreased the social interaction ratio (Figure 1K), and increased the immobility time in TST and FST, without affecting the locomotor activity of SSDS‐treated mice in OFT (Figure 1L–N), indicating that stimulation of MS to DG projection facilitates the susceptibility of mice to stress.

### 2.2 Inhibition of MS^GABA+^‐DG Projection Induces Expression of PDGF‐BB in SOM Interneurons Resulting in Antidepressant‐Like Effects

Adult neurogenesis in the DG of hippocampus is functionally implicated in behavioral responses to stress and antidepressants. It has been reported that inhibition of MS‐DG GABAergic neuron is involved in modulation of adult hippocampal neurogenesis.^[^ ^6^ ^]^ In order to better understand how MS‐DG projection was involved in depressive‐like behaviors, the differentially expressed genes (DEGs) in two Gene Expression Omnibus (GEO) datasets with those related to neurogenesis (GSE8091) and depression (GSE151807) were analyzed. The results showed that 346 and 12138 DEGs were uniquely expressed in each group and 855 DEGs were co‐expressed in both group (Figure S6A, Supporting Information). The Kyoto Encyclopedia of Genes and Genomes (KEGG) pathway enrichment analysis indicated that the biological processes of 855 DEGs were involved in the development of nervous system, regulation of cell cycle, and PDGFR signaling pathway, and so on (Figure S6B, Supporting Information). PDGF can act as a mitogen in the early phase of neural precursor cells differentiation to expand the pool of immature neurons.^[^ ^19^ ^]^ PDGF‐AA has been shown to function as a potent mitogen for oligodendrocyte progenitors.^[^ ^20^ ^]^ PDGF‐CC has emerged as a new player for its potential therapeutic efficacy in various neurodegenerative diseases, such as Alzheimer's disease and Parkinson's disease.^[^ ^21^ ^]^ Previous studies have demonstrated that PDGF‐BB‐mediated signaling regulates diverse functions in the central nervous system, such as neurogenesis,^[^ ^22^ ^]^ cell survival,^[^ ^23^ ^]^ and synaptogenesis.^[^ ^24^ ^]^ These findings prompted us to examine whether PDGF‐BB could participate in the regulation of depressive‐like behaviors in MS‐DG circuit. As shown in Figure S6, Supporting Information, the mRNA and protein expression of PDGF‐BB was decreased in the DG of CSDS‐treated mice (Figure S6C,D, Supporting Information). We then elucidated the underlying mechanisms. Inhibition of MS to DG projection increased the mRNA level of PDGF‐BB in CNO‐treated mice exposing to CSDS (Figure S6E, Supporting Information). Conversely, CNO treatment decreased the mRNA level of PDGF‐BB in the SSDS‐treated mice expressing hM3Dq (Figure S6F, Supporting Information), suggesting the MS‐DG pathway is the direct upstream modulator of PDGF‐BB expression.

Accumulating evidence demonstrates that dorsal hippocampus receives GABAergic input from MS.^[^ ^25^ ^]^ Thus, we targeted the GABAergic MS neurons of Vgat‐Cre mice with expression of AAV‐DIO‐hM4Di‐mCherry and cannulated onto the terminal areas in the DG to explore the role of MS^GABA+^‐DG projection in depressive‐like behaviors (Figure S6G, Supporting Information). Images obtained by confocal microscopy showed a selective and restricted expression of AAV‐DIO‐hM4Di‐mCherry in the GABAergic neurons from Vgat‐Cre mice in MS and a high GABAergic fiber density in DG (Figure S6H,I, Supporting Information). We found that selective inhibition of MS^GABA+^‐DG projection in Vgat‐Cre mice by CNO decreased immobility time in both TST and FST, with no effect on locomotor activity (Figure S6J–L, Supporting Information), indicating that the inhibition of MS^GABA+^‐DG projection improves depressive‐like behaviors. Furthermore, CNO‐mediated selective inhibition of MS^GABA+^‐DG projection increased the mRNA level of PDGF‐BB in Vgat‐Cre mice (Figure S6M, Supporting Information).

**Figure 2:**
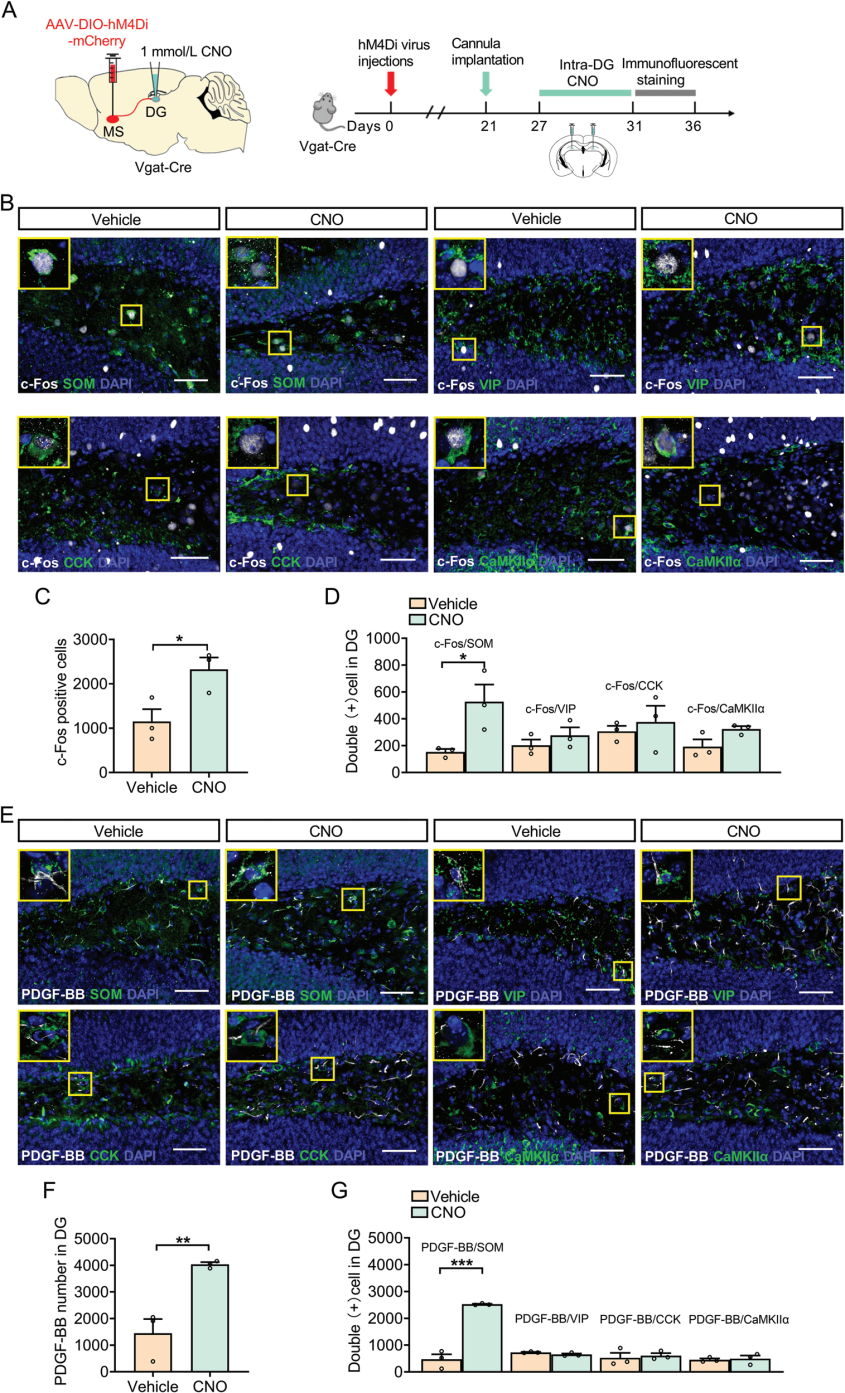
Inhibition of MS^GABA+^‐DG projection increases PDGF‐BB expression in SOM‐positive neurons. A) Schematic of viral infusion and cannula implantation. Timeline of experimental procedure. B) Double‐stained c‐Fos (white) with SOM, VIP, CCK, and CaMKII*α* (green) in the DG of mice treated with vehicle or CNO. Panels on the left are magnified images showing the colocalization of c‐Fos expression with SOM, VIP, CCK, and CaMKII*α*. Scale bars: 50 µm. C) DREADD inhibition of MS^GABA+^‐DG projection led to an increase in c‐Fos expression in the DG. *n* = 3 per group. D) DREADD inhibition of MS^GABA+^‐DG projection led to an increase in c‐Fos expression in SOM‐positive neurons. *n* = 3 per group. E) Double‐stained PDGF‐BB (white) and SOM‐, VIP‐, CCK‐, and CaMKII*α* (green)‐positive neurons in the DG of mice treated with vehicle or CNO. Panels on the left are magnified images showing the colocalization of PDGF‐BB expression with SOM, VIP, CCK, and CaMKII*α*. Scale bars: 50 µm. F) DREADD inhibition of MS^GABA+^‐DG projection led to an increase in PDGF‐BB expression in DG. *n* = 3 per group. G) DREADD inhibition of MS^GABA+^‐DG projection led to an increase in PDGF‐BB expression in SOM‐positive neurons. *n* = 3 per group. Data are expressed as mean ± SEM. Student's *t‐*test (C, D, F, G). *p < 0.05, **p < 0.01, ***p < 0.001. The statistical details can be found in Table S4, Supporting Information.

In order to determine which cell types of the DG are involved in the functions of PDGF‐BB described above, immunofluorescent staining was performed from coronal brain sections of Vgat‐Cre mice expressed hM4Di using a set of neuronal markers, including CCK, vasoactive intestinal peptide (VIP), calmodulin‐dependent protein kinase II*α* subtype (CaMKII*α*), and SOM combined with PDGF‐BB immunostaining (**Figure** **2**A,B). We assessed the activation of DG neuron by analysis of the immediate early gene c‐Fos, which is used as a marker of neuronal activation. Immunofluorescent analysis showed that inhibition of MS^GABA+^‐DG projection increased the expression of c‐Fos in the DG (Figure 2B,C). Importantly, CNO treatment only significantly increased the expression of c‐Fos in SOM‐positive neurons, but not in VIP‐, CCK‐, or CaMKII*α*‐positive neurons (Figure 2D). Therefore, we evaluated whether inhibition of the MS to DG projection could increase PDGF‐BB expression in SOM‐positive neurons. After CNO treatment, the results revealed that inhibition of MS^GABA+^‐DG projection‐induced PDGF‐BB immunoreactivity was detected in a significantly higher level of SOM neurons, whereas there was no change in PDGF‐BB expression in VIP‐, CCK‐, CaMKII*α*‐positive neurons than that of control groups (Figure 2E–G). These results indicate that the increased activity of SOM‐positive neurons via inhibition of MS^GABA+^‐DG projection induces PDGF‐BB expression in the DG.

### 2.3 Overexpression of PDGF‐BB Reverses CSDS‐Induced Depressive‐Like Behaviors

**Figure 3:**
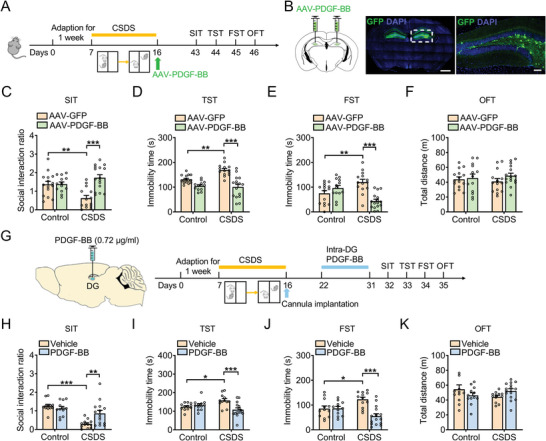
Overexpression of PDGF‐BB reverses CSDS‐induced depressive‐like behaviors of mice. A) Experimental timeline of viral infusion, CSDS protocol, and behavioral tests. B) Representative photomicrographs of injection sites in the hippocampal DG and panels on the right are infected neurons. Scale bars: 1000 µm (left), 100 µm (right). C) Effects of PDGF‐BB overexpression on the social interaction ratio in SIT. *n* = 13–16 per group. D) Effects of PDGF‐BB overexpression on immobility time in TST. *n* = 13–16 per group. E) Effects of PDGF‐BB overexpression on the immobility time in FST. *n* = 13–16 per group. F) Effects of PDGF‐BB overexpression on the locomotor activity in OFT. *n* = 13–16 per group. G) Schematic timeline of CSDS protocol, PDGF‐BB treatment, and behavioral tests. H) Effects of PDGF‐BB treatment on the social interaction ratio in SIT. *n* = 12–15 per group. I) Effects of PDGF‐BB treatment on the immobility time in TST. *n* = 12–15 per group. J) Effects of PDGF‐BB treatment on the immobility time in FST. *n* = 12–15 per group. K) Effects of PDGF‐BB treatment on the locomotor activity in OFT. *n* = 12–15 per group. Data are expressed as mean ± SEM. Two‐way ANOVA followed by the Bonferroni's post hoc test (C–F, H–K). *p < 0.05, **p < 0.01, ***p < 0.001. The statistical details can be found in Table S4, Supporting Information.

To investigate the effect of PDGF‐BB on depressive‐like behaviors, a recombinant AAV viral vectors encoding PDGF‐BB (AAV‐PDGF‐BB) were used to overexpress PDGF‐BB in the DG after the mice were exposed to CSDS (**Figure** **3**A,B). The expression efficiency of AAV‐PDGF‐BB were examined by ELISA in naïve mice (Figure S7, Supporting Information). We found that, compared with AAV‐GFP‐treated mice, the overexpression of PDGF‐BB in the DG increased social interaction ratio in SIT and reduced immobility time in FST and TST of defeated mice without affecting locomotor activity (Figure 3C–F). Next, recombinant PDGF‐BB protein was administrated into DG bilaterally (0.0072/0.072/0.72 µg mL^−1^) for 3 days in naïve mice to further validate the antidepressant effects of PDGF‐BB. It was found that acute treatment with recombinant PDGF‐BB protein (0.72 µg mL^−1^) decreased immobility time in the FST, but not locomotor activity (Figure S8, Supporting Information). For chronic administration, recombinant PDGF‐BB protein was locally injected into the DG bilaterally at a dose of 0.72 µg mL^−1^ daily for 10 days reversed the CSDS‐induced depressive‐like behaviors characterized by decreased social interaction ratio in SIT, increased immobility time in TST and FST (Figure 3G–J), however, microinjection of PDGF‐BB had no effect on basal locomotor activity (Figure 3K), indicating that the elevation of PDGF‐BB level in the DG promotes the resilience of mice to chronic stress.

### 2.4 Knockdown of PDGF‐BB in the DG Promotes the Susceptibility of Mice to Stress

**Figure 4:**
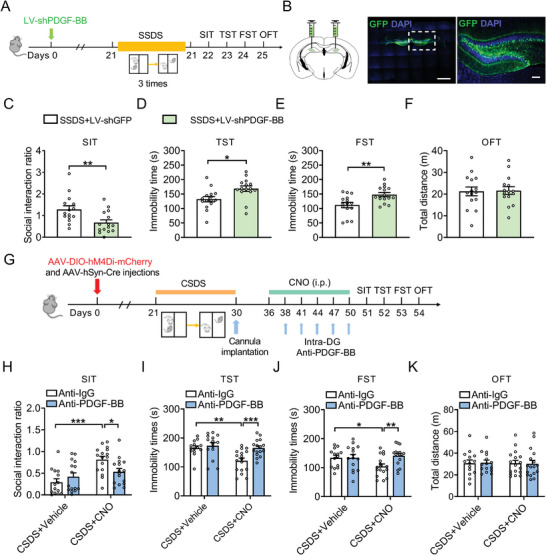
Downregulation of PDGF‐BB in the DG promotes the susceptibility of mice to stress. A) Timeline of experiments of virus injection, SSDS protocol, and behavioral tests. B) Representative photomicrographs of injection sites in the hippocampal DG and panels on the right are infected neurons. Scale bars: 1000 µm (left), 100 µm (right). C) Effects of PDGF‐BB knockdown on the social interaction ratio of SIT in the mice exposed to SSDS. *n* = 15 per group. D) Effects of PDGF‐BB knockdown on the immobility time of TST in the mice exposed to SSDS. *n* = 15 per group. E) Effects of PDGF‐BB knockdown on the immobility time of FST in the mice exposed to SSDS. *n* = 15 per group. F) Effects of PDGF‐BB knockdown on the locomotor activity in OFT in the mice exposed to SSDS. *n* = 15 per group. G) Experimental design for anti‐PDGF‐BB treatment. H) Effects of CNO treatment for anti‐PDGF‐BB or anti‐IgG treated mice following CSDS on the social interaction ratio in SIT. *n* = 14–16 per group. I) Effects of CNO treatment for anti‐PDGF‐BB or anti‐IgG treated mice following CSDS on the immobility time in TST. *n* = 14–17 per group. J) Effects of CNO treatment for anti‐PDGF‐BB or anti‐IgG treated mice following CSDS on the immobility time in FST. *n* = 14–17 per group. K) Effects of CNO treatment for anti‐PDGF‐BB or anti‐IgG treated mice following CSDS on the locomotor activity in OFT. *n* = 14–17 per group. Data are expressed as mean ± SEM. Student's *t‐*test (C–F), Two‐way ANOVA followed by the Bonferroni's post hoc test (H–K). *p < 0.05, **p < 0.01, ***p < 0.001. The statistical details can be found in Table S4, Supporting Information.

To determine whether lack of PDGF‐BB in the DG contributed to depressive‐like behaviors, lentivirus‐mediated expression of specific short hairpin RNA (shRNA) against PDGF‐BB (LV‐shPDGF‐BB) was stereotaxically infused into the bilateral DG of naïve mice (**Figure** **4**A,B) and decrease the expression of DPGF‐BB which confirmed by ELISA (Figure S9A,B, Supporting Information). The behavioral results showed when exposed to SSDS, LV‐shPDGF‐BB‐treated mice displayed a reduction in social interaction ratio and increased immobility time in the TST and FST, with little effect on locomotor activity compared with that of LV‐shGFP‐treated mice (Figure 4C–F). However, LV‐shPDGF‐BB did not induce behavioral abnormalities in the naïve mice (Figure S9C–F, Supporting Information). These results suggest that PDGF‐BB knockdown in the DG facilitates the susceptibility of mice to stress.

To further solidify the role of PDGF‐BB signaling specifically at the MS‐DG pathway in the regulation of CSDS‐induced depressive‐like behavior, a well‐characterized neutralizing antibody against PDGF‐BB (anti‐PDGF‐BB) was continuously infused into the DG of defeated mice expressing hM4Di for 15 days (Figure 4G).^[^ ^26^ ^]^ The results showed that CSDS‐treated mice expressing hM4Di infused with anti‐PDGF‐BB to the DG showed social avoidance with a lower social interaction ratio in SIT, whereas CSDS‐treated mice expressing hM4Di infused with IgG did not (Figure 4H). Immobility was increased in CSDS‐treated mice expressing hM4Di infused with anti‐PDGF‐BB compared with CSDS‐treated mice expressing hM4Di infused with IgG in both TST and FST (Figure 4I,J). There was no effect on locomotor activity (Figure 4K). These results provide evidence that the loss of PDGF‐BB in DG blocks the antidepressant action of inhibition of MS‐DG projection.

### 2.5 Overexpression of PDGF‐BB in the DG Prevents CSDS‐Induced Deficiency in Hippocampal Neurogenesis

Considering that stress‐induced deficiency in adult hippocampal neurogenesis contributes to the pathogenesis of depression, which is reversed by antidepressant treatments and PDGF‐BB prevents hippocampal neuronal cell death and promote neurogenesis,^[^ ^27^ ^]^ we therefore investigated the neurogenic effects of PDGF‐BB in the hippocampus of stressed mice. By using bromodeoxyuridine (BrdU) and doublecortin (DCX) to identify proliferating cells and immature neurons, respectively, it was found that the numbers of BrdU positive and BrdU/DCX positive cells were lower in the subgranular zone (SGZ) of hippocampal DG in defeated mice than that in control mice (Figure S10A–D, Supporting Information), which were reversed by recombinant PDGF‐BB treatment (Figure S10B–D, Supporting Information). Then, we assessed the effect of PDGF‐BB overexpression on the population of radial glia‐like stem cells and nonracial progenitors. As shown in Figure S10E–H, recombinant PDGF‐BB increased the numbers of BrdU/Sox2/GFAP positive cells, a marker of activated NSCs (aNSCs), but not the Sox2/GFAP positive cells, a marker of quiescent NSCs (qNSCs), in the DG of defeated mice, indicating that PDGF‐BB selectively increases the number of aNSCs, with no effect on qNSCs. Furthermore, there was a significant increment in the ratio of aNSCs to qNSCs in PDGF–BB–treated defeated mice compared with Vehicle–treated defeated mice. Considering that newborn neurons in the DG functionally integrate into existing hippocampal circuits and influence hippocampus‐dependent learning and memory,^[^ ^28^ ^]^ the retroviral vector expressing GFP (ROV‐GFP) were stereotactically injected into DG to observe the dendritic growth of newborn neurons in adult mice (Figure S10I, Supporting Information). Four weeks later, the reduction in both dendritic length, number of branch points and intersectionswere observed in ROV‐GFP‐labeled newborn neurons of CSDS‐treated mice, which was reversed by exogenous administration of recombinant PDGF‐BB protein into the DG (Figure S10J–M, Supporting Information). These results suggest that PDGF‐BB is required for the maturation of newborn neurons in adult hippocampal DG.

**Figure 5:**
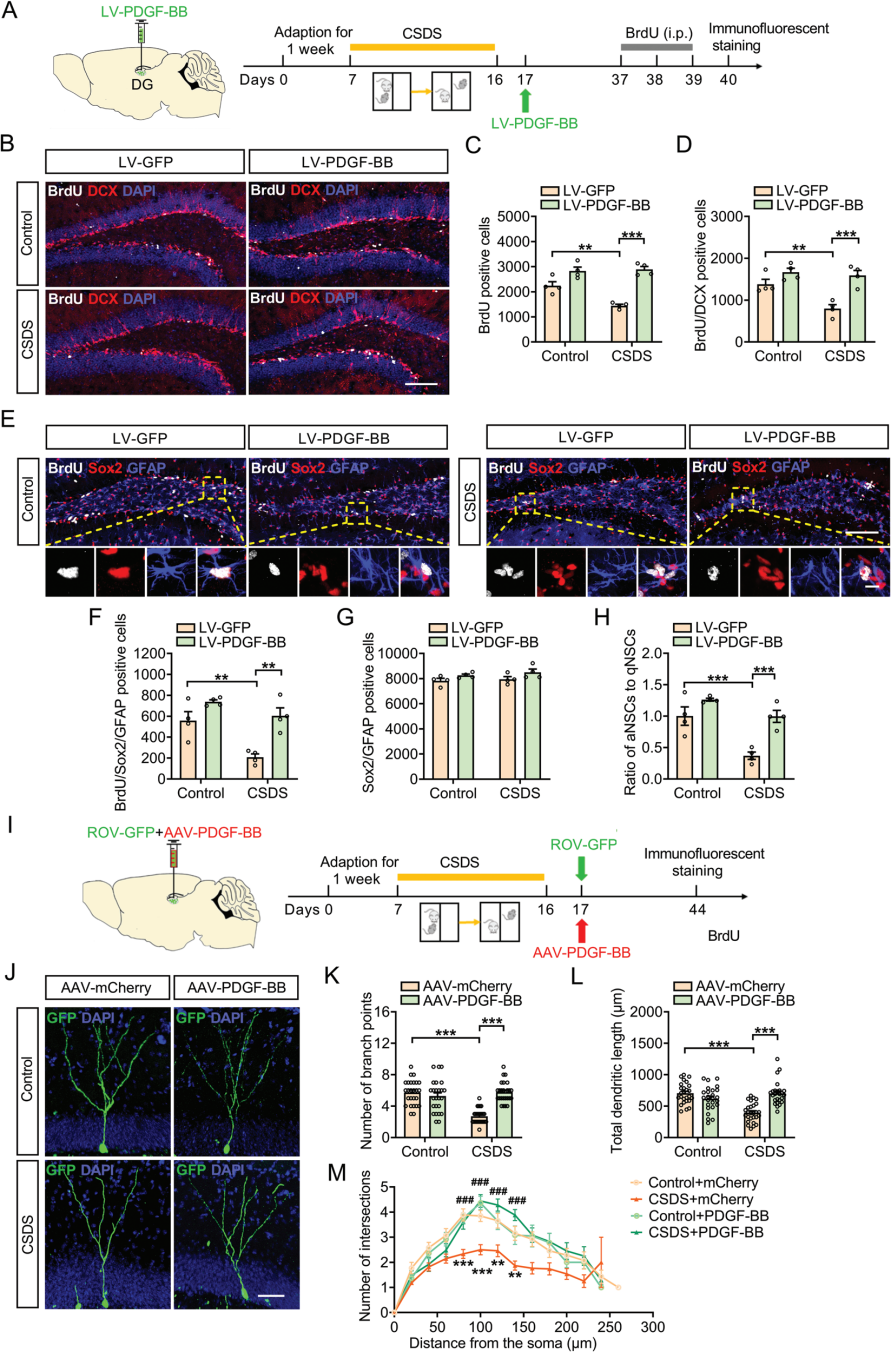
Stress‐induced deficits in the adult hippocampal neurogenesis is blocked by PDGF‐BB overexpression. A) Experimental timelines for CSDS protocol, viral infection, and the BrdU injection protocol. B) Immunofluorescence images for BrdU (white), DCX (red) within the SGZ of the hippocampus in control or defeated mice injected with LV‐GFP or LV‐PDGF‐BB. Scale bar: 100 µm. C) Quantification of the number of BrdUpositive cells in SGZ of hippocampus after PDGF‐BB overexpression. *n* = 4 per group. D) Quantification of the number of BrdU/DCXpositive cells in SGZ of the hippocampus after PDGF‐BB overexpression. *n* = 4 per group. E) Immunofluorescence images for BrdU (white), Sox2 (red), and GFAP (blue) within the SGZ of the hippocampus in in control or defeated mice injected with LV‐GFP or LV‐PDGF‐BB. Panels on the below are magnified images showing that the colocalization of BrdU expression with GFAP and Sox2. Scale bar: 100 µm (top), 10 µm (below). F) Quantification of the number of BrdU/Sox2/GFAPpositive NSCs in SGZ of the hippocampus after PDGF‐BB overexpression. *n* = 4 per group. G) Quantification of the number of Sox2/GFAPpositive NSCs in SGZ of the hippocampus after PDGF‐BB overexpression. *n* = 4 per group. H) The ratio of aNSCs to qNSCs. *n* = 4 per group. I) Experimental timelines for CSDS protocol, PDGF‐BB treatment, and the virus injection protocol. J) Representative images of ROV‐GFP‐labeled newborn neurons in AAV‐mCherry or AAV‐PDGF‐BB‐injected mice 4 weeks after retrovirus injection. Scale bars: 50 µm. K) Quantification of branch number of ROV‐GFP‐labeled newborn neurons in AAV‐mCherry or AAV‐PDGF‐BB‐injected mice 4 weeks after retrovirus injection. *n* = 25–27 neurons per group. L) Quantification of dendritic length of ROV‐GFP‐labeled newborn neurons in AAV‐mCherry or AAV‐PDGF‐BB‐injected mice 4 weeks after retrovirus injection. *n* = 25–27 neurons per group. M) Sholl analysis of dendritic complexity of ROV‐GFP‐labeled newborn neurons in AAV‐mCherry or AAV‐PDGF‐BB‐injected mice 4 weeks after retrovirus injection. *n* = 23–27 neurons per group. Data are expressed as mean ± SEM. Two‐way ANOVA followed by the Bonferroni's post hoc test (C, D, F–H, K–M). *p < 0.05, **p < 0.01, ***p < 0.001. The statistical details can be found in Table S4, Supporting Information.

Next, we infused LV‐PDGF‐BB into the DG of mice to further investigate the role of PDGF‐BB in regulation of adult hippocampal neurogenesis. We labeled the proliferating cells with the BrdU, 24 h prior to perfusion (**Figure** **5**A). It was shown that microinfusion of LV‐PDGF‐BB into the DG increased the numbers of BrdU, BrdU/DCX, and BrdU/Sox2/GFAP positive cells of defeated mice compared with that of LV‐GFP‐treated defeated mice (Figure 5B–F). Importantly, overexpression of PDGF‐BB did not affect the number of Sox2/GFAP positive cells (Figure 5G), but it did significantly increase the proportion of BrdU/Sox2/GFAP to Sox2/GFAP positive cells in LV‐PDGF‐BB‐injected mice exposure to CSDS relative to the LV‐GFP administration (Figure 5H). Moreover, transduced newborn neurons (ROV‐GFP) in defeated mice injected with AAV‐PDGF‐BB featured a more complicated dendritic arbor that was characterized by an increase in the total dendrite length, branch points, and number of intersections than defeated mice injected with AAV‐mCherry (Figure 5I–M). These results suggest that the elevation of PDGF‐BB level in the DG ameliorates chronic stress‐induced defective hippocampal neurogenesis, which contributes to the antidepressant effects of PDGF‐BB.

### 2.6 Downregulation of PDGF‐BB Impairs Hippocampal Neurogenesis and Morphogenesis of Dendrites in DG

**Figure 6:**
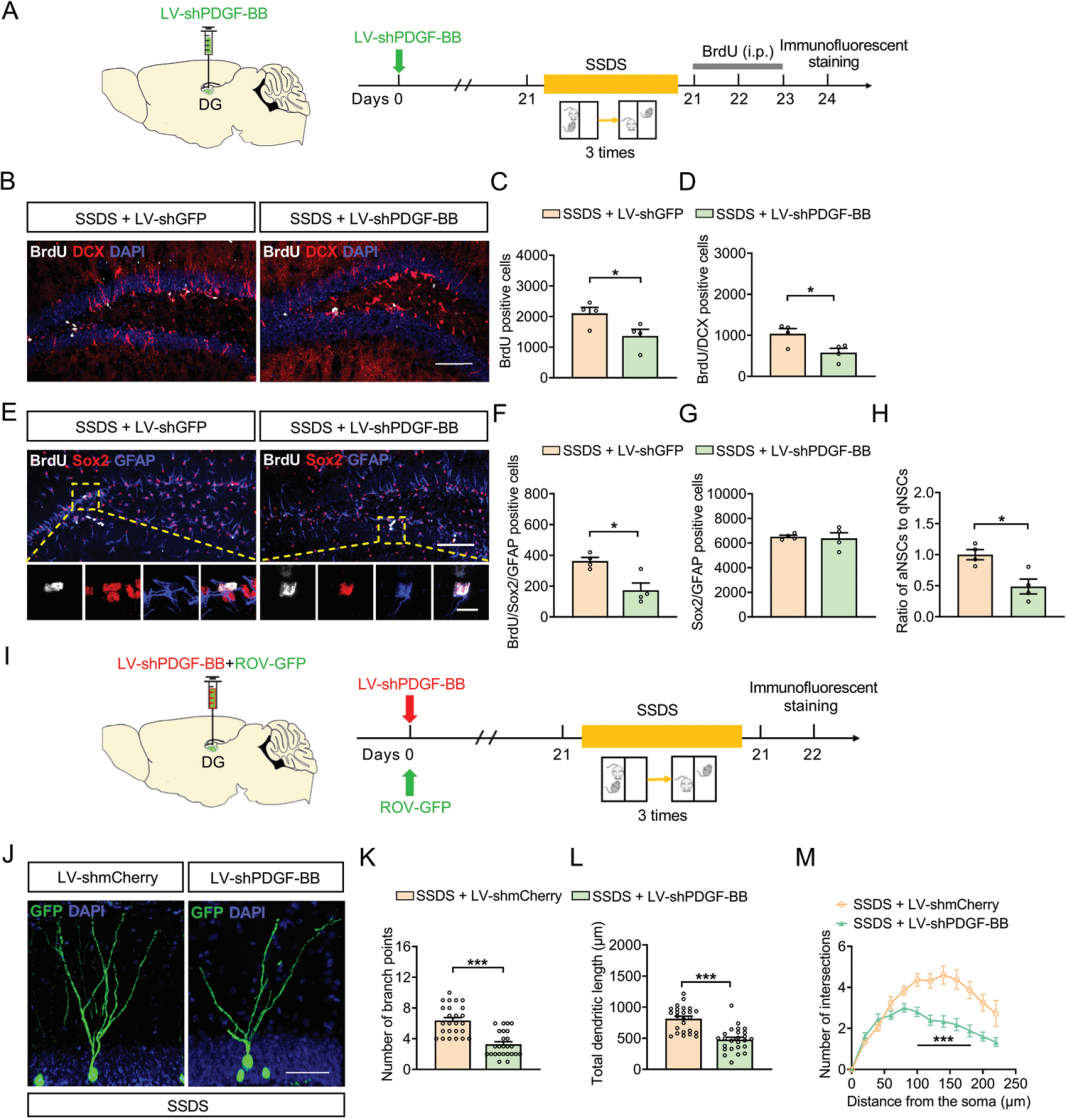
Downregulation of PDGF‐BB impairs adult hippocampal neurogenesis and dendritic morphogenesis of DG. A) Experimental timelines for viral infection, SSDS protocol, and BrdU injection. B) Immunofluorescence images for BrdU (white), DCX (red) within the SGZ of the hippocampus in control or SSDS‐treated mice injected with LV‐shGFP or LV‐shPDGF‐BB. Scale bar: 100 µm. C) Quantification of the number of BrdUpositive cells in the SGZ of hippocampus of mice injected with LV‐shGFP or LV‐shPDGF‐BB. *n* = 4 per group. D) Quantification of the number of BrdU/DCXpositive cells in the SGZ of hippocampus of mice injected with LV‐shGFP or LV‐shPDGF‐BB. *n* = 4 per group. E) Immunofluorescence images for BrdU (white), Sox2 (red), and GFAP (blue) in the SGZ of hippocampus of control or defeated injected with LV‐shGFP or LV‐shPDGF‐BB. Panels on the below are magnified images showing that the colocalization of BrdU expression with GFAP and Sox2. Scale bar: 100 µm (top), 10 µm (below). F) Quantification of the number of BrdU/Sox2/GFAPpositive NSCs in the SGZ of hippocampus of mice injected with LV‐shGFP or LV‐shPDGF‐BB. *n* = 4 per group. G) Quantification of the number of Sox2/GFAPpositive NSCs in the SGZ of hippocampus of mice injected with LV‐shGFP or LV‐shPDGF‐BB. *n* = 4 per group. H) The ratio of aNSCs to qNSCs. *n* = 4 per group. I) Experimental timelines for viral infection and SSDS protocol. J) Representative images of ROV‐GFP labeled newborn neurons in LV‐shmCherry or LV‐shPDGF‐BB‐injected stressed mice 4 weeks after retrovirus injection. Scale bars = 50 µm. K) Quantification of branch number of ROV‐GFP labeled newborn neurons in LV‐shmCherry or LV‐shPDGF‐BB‐injected stressed mice 4 weeks after retrovirus injection. *n* = 25 neurons per group. L) Quantification of dendritic length of ROV‐GFP labeled newborn neurons in LV‐shmCherry or LV‐shPDGF‐BB‐injected stressed mice 4 weeks after retrovirus injection. *n* = 25 neurons per group. M) Sholl analysis of dendritic complexity of ROV‐GFP labeled newborn neurons in LV‐shmCherry or LV‐shPDGF‐BB‐injected stressed mice 4 weeks after retrovirus injection. *n* = 17–25 neurons per group. All data are presented as the mean ± SEM. Student's *t‐*test (C, D, F–H, K–M). *p < 0.05, **p < 0.01, ***p < 0.001. The statistical details can be found in Table S4, Supporting Information.

To determine whether downregulation of DG levels of PDGF‐BB can cause a deficiency in adult hippocampal neurogenesis, LV‐shPDGF‐BB or LV‐shGFP virus was injected bilaterally into DG regions of mice. Three weeks after viral injections, mice were subjected to SSDS paradigm (**Figure** **6**A). After a 24 h‐pulse of BrdU incorporation, there were less BrdU positive proliferating cells and BrdU/DCX positive newborn neurons in the DG of LV‐shPDGF‐BB‐injected SSDS‐treated mice compared with LV‐shGFP‐injected SSDS‐treated mice (Figure 6B–D). Moreover, there were fewer number of BrdU/Sox2/GFAP positive cells in the DG of the LV‐shPDGF‐BB‐injected SSDS‐treated mice (Figure 6E,F). Although there was no difference in the number of Sox2/GFAP positive cells between LV‐shPDGF‐BB‐ and LV‐shGFP‐injected SSDS‐treated mice (Figure 6G), the proportion of aNSCs to qNSCs was lower in the LV‐shPDGF‐BB‐injected SSDS‐treated mice than that in LV‐shGFP‐injected SSDS‐treated mice (Figure 6H), indicating that PDGF‐BB deficiency in DG induces the dormant state of NSCs. We further tested the effect of knockdown PDGF‐BB on the dendritic development of newborn neurons in the DG of SSDS‐treated mice. It was found that ROV‐GFP‐labeled newborn neurons in LV‐shPDGF‐BB‐injected SSDS‐treated mice displayed less branch number, shorter dendrites, and less dendritic complexity compared with that of LV‐shmCherry‐injected SSDS‐treated mice (Figure 6I–M). The above results suggest that reducing the expression of PDGF‐BB in the DG impairs adult hippocampal neurogenesis in response to stress, which may be responsible for the facilitation of sensitivity to stress.

### 2.7 Specific Knockdown of PDGFR*β* in NSCs Leads to Depressive‐Like Behaviors through Impairing Adult Hippocampal Neurogenesis

**Figure 7:**
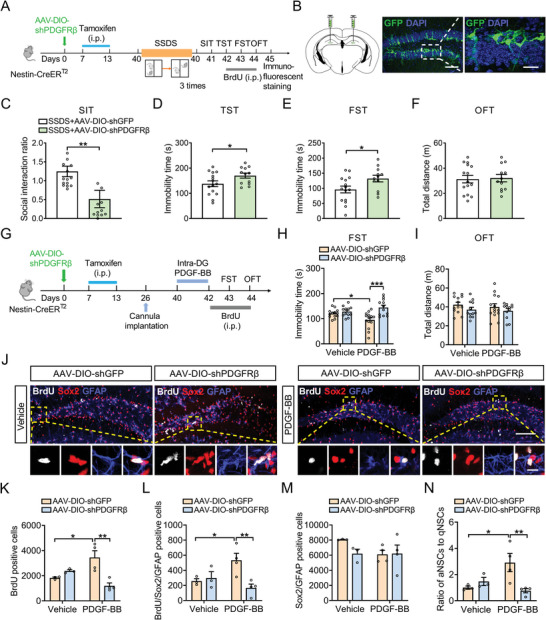
Knockdown of PDGFR*β* from NSCs leads to depressive‐like behaviors through impairing adult hippocampal neurogenesis. A) Experimental design of virus‐mediated delivery of PDGFR*β* gene into Nestin‐CreER^T2^ mice, and the BrdU injection protocol. B) Stereotaxic injection of AAV‐DIO‐shGFP or AAV‐DIO‐shPDGFR*β* into DG. Scale bar: 100 µm (left), 20 µm (right). C) Effects of PDGFR*β* knockdown after SSDS on the social interaction ratio in SIT. *n* = 12–15 neurons per group. D) Effects of PDGFR*β* knockdown after SSDS on the immobility time in TST. *n* = 12–15 neurons per group. E) Effects of PDGFR*β* knockdown after SSDS on the immobility time in FST. *n* = 12–15 neurons per group. F) Effects of PDGFR*β* knockdown after SSDS on the locomotor activity in OFT. *n* = 12–15 neurons per group. G) Schematic timeline of viral infection, PDGF‐BB treatment and BrdU injection. H) Effects of PDGF‐BB treatment on the immobility time in FST for AAV‐DIO‐shGFP and AAV‐DIO‐shPDGFR*β*‐injected mice. *n* = 12–14 per group. I) Effects of PDGF‐BB treatment on the locomotor activity in the OFT for AAV‐DIO‐shGFP and AAV‐DIO‐shPDGFR*β*‐injected mice. *n* = 12–14 per group. J) Immunofluorescence images for GFAP (blue), Sox2 (red), and BrdU (white) in the SGZ of the hippocampus in AAV‐DIO‐shGFP and AAV‐DIO‐shPDGFR*β*‐injected mice treated with vehicle or PDGF‐BB. Panels on the below are magnified images showing that the colocalization of BrdU expression with GFAP and Sox2. Scale bar: 100 µm (top), 10 µm (below). K) Quantification of the number of BrdUpositive cells in the DG of hippocampus in AAV‐DIO‐shGFP and AAV‐DIO‐shPDGFR*β*‐injected mice treated with vehicle or PDGF‐BB. *n* = 3–4 per group. L) Quantification of the number of BrdU/Sox2/GFAPpositive NSCs in the SGZ of the hippocampus in AAV‐DIO‐shGFP and AAV‐DIO‐shPDGFR*β*‐injected mice treated with vehicle or PDGF‐BB. *n* = 3–4 per group. M) Quantification of the number of Sox2/GFAPpositive NSCs in the SGZ of the hippocampus in AAV‐DIO‐shGFP and AAV‐DIO‐shPDGFR*β*‐injected mice treated with vehicle or PDGF‐BB. *n* = 3–4 per group. N) The ratio of aNSCs to qNSCs in the SGZ of the hippocampus in AAV‐DIO‐shGFP and AAV‐DIO‐shPDGFR*β*‐injected mice treated with vehicle or PDGF‐BB. *n* = 3–4 per group. Data are expressed as mean ± SEM. Student's *t‐*test (C–F), Two‐way ANOVA followed by the Bonferroni's post hoc test (H, I, K–N). *p < 0.05, **p < 0.01, ***p < 0.001. The statistical details can be found in Table S4, Supporting Information.

Considering that PDGF‐BB exerts its biological functions through binding with PDGFR*β* that has been found to be expressed by adult ventricular‐subventricular zone,^[^ ^29^ ^]^ we first investigate the role of PDGFR*β* in depressive‐like behaviors, the level of PDGFR*β* in the DG of defeated mice was measured. It was found that the expression of PDGFR*β* protein was decreased in the DG of CSDS‐treated mice (Figure S11A, Supporting Information). Next we asked whether PDGFR*β* is expressed in NSCs of hippocampal DG. Immunofluorescent staining with Nestin and PDGFR*β* antibodies showed that the immunofluorescence of PDGFR*β* was observed in the NSCs (Figure S11B,C, Supporting Information). Furthermore, PDGFR*β* was conditionally knocked down in NSCs by injecting Cre‐dependent AAV‐DIO‐shPDGFR*β* into DG of adult Nestin‐CreER^T2^ mice (Figure S11D,E, Supporting Information). Four weeks after tamoxifen induction, mice injected with control or PDGFR*β* shRNA virus were subjected to SSDS (**Figure** **7**A,B). Behavioral results showed that the downregulation of PDGFR*β* in NSCs resulted in an obvious increase in social avoidance (Figure 7C). Meanwhile, SSDS‐treated mice with PDGFR*β* shRNA spent more time immobile in the TST and FST (Figure 7D,E), with no change in locomotor activity (Figure 7F). More importantly, acute administration of recombinant PDGF‐BB protein daily for 3 days decreased immobility time in the FST, which was blunted by knockdown of PDGFR*β* in the NSCs, with no effects on locomotor activity (Figure 7G–I). These findings support the notion that the ablation of PDGFR*β* in the adult NSCs of DG promotes the susceptibility of mice to stress.

Given that the deficiency of adult neurogenesis was induced by CSDS, we wondered whether the decreased PDGFR*β* signaling is involved in this process. AAV‐DIO‐shPDGFR*β* or AAV‐DIO‐shGFP was injected into DG of Nestin‐CreER^T2^ mice. Four weeks after the AAV injections, we found that PDGFR*β* downregulation induced a relative reduction in proliferating cells (BrdU positive cells), aNSCs (GFAP/Sox2/BrdU positive cells), and the ratio of aNSCs to qNSCs in mice exposed to SSDS (Figure S11F–I, Supporting Information). However, the loss of PDGFR*β* did not affect the number of Sox2/GFAP positive cells (Figure S11J, Supporting Information), indicating that the decrease in protein level of PDGFR*β* in NSCs contributes to the impairment of hippocampal neurogenesis on stressed condition. To further investigate whether the downregulation of PDGFR*β* in the NSCs could inhibit the promoting effects of PDGF‐BB on hippocampal neurogenesis, AAV‐DIO‐shPDGFR*β* or AAV‐DIO‐shGFP‐injected mice was infused either vehicle or PDGF‐BB into DG after tamoxifen injection. It was shown that PDGF‐BB promoted the proliferation of NSCs depicted by increase in BrdU and BrdU/Sox2/GFAP positive cells, and the ratio of aNSCs to qNSCs, which were completely attenuated by downregulation of PDGFR*β* in the NSCs (Figure 7J–N). Hence, the above results suggest that PDGFR*β* in the DG NSCs is required for PDGF‐BB‐enhanced hippocampal neurogenesis and antidepressant effects.

To gain insights into how PDGF‐BB/PDGFR*β* regulates adult hippocampal neurogenesis and depressive‐like behaviors, we employed the search tool for the retrieval of interaction gene/proteins (STRING) database to identify the most connected functional proteins with PDGFR*β* (Figure S12A, Supporting Information). Gene set enrichment analysis (GSEA) showed a significant increase in the expression of signal transducer and activator of transcription 3 (STAT3), signal transducer and activator of transcription 5 (STAT5), and Kirsten rat sarcoma viral oncogene homolog (Kras) in fluoxetine‐treated mice, all of which have been shown to be interacted with PDGFR*β* (Figure S12B–D, Supporting Information). The Janus kinase‐signal transducer and activator of transcription (JAK‐STAT) signaling pathway has been reported to be involved in brain development.^[^ ^30^ ^]^ The results showed that microinjection of recombinant PDGF‐BB protein into DG daily for 3 days increased the expression of JAK2 and STAT3 in the DG (Figure S12E–G, Supporting Information). Consistent with the in vivo results, treatment of the neurosphere cultures with PDGF‐BB increased the expression of JAK2 and STAT3 protein (Figure S12H–J, Supporting Information), indicating that JAK2/STAT3 signaling pathway might be the downstream pathway of PDGF‐BB/PDGFR*β*.

## 3 Discussion

In the present study, we discovered that circuit‐specific PDGF‐BB/PDGFR*β* signaling collapse at MS‐DG pathway linked chronic stress to depressive‐like behaviors in mice. Specifically, we found that the inhibition of MS^GABA+^‐DG projection protected against chronic stress‐induced depressive‐like behaviors by increasing PDGF‐BB expression from SOM‐positive neurons in the DG. Importantly, overexpression of PDGF‐BB or exogenous administration of PDGF‐BB in the DG that bound with PDGFR*β* in the NSCs promoted the adult hippocampal neurogenesis and ameliorated the depressive‐like behaviors. These studies identified a critical role of the MS^GABA+^‐DG projection in the generation of depressive‐like behaviors after chronic stress, and revealed a PDGF‐BB‐mediated mechanism underlying the improvement of chronic stress‐induced impairment of hippocampal neurogenesis and behavioral abnormality.

Exposure to chronic stress is one of the most critical factors in the onset of depressive disorders.^[^ ^31^ ^]^ Altered hippocampal neurogenesis has been implicated in the precipitation of anxiety‐ and depressive‐like behaviors of rodent models of psychiatric diseases, as well as in the improving effects produced by different classes of antidepressants, trophic factor, or physiological stimuli, such as physical exercise.^[^ ^32^ ^]^ Recent work demonstrates that network activity plays a critical role in the survival of newly generated neurons and hippocampal neurogenesis in the adult brain.^[^ ^5^, ^6^, ^32^ ^]^ The MS is one of the major downstream targets of projection from the hippocampus and has been implicated as a neural substrate of anxiety and mood‐related behavior.^[^ ^33^ ^]^ Stimulation of MS‐DG cholinergic pathway ameliorates the deficits of spatial memory.^[^ ^34^ ^]^ Optogenetic stimulation of septohippocampal terminals or selective chemogenetic activation of acetylcholine‐positive inputs to hippocampus increases stress‐related behaviors,^[^ ^35^ ^]^ however, to date, the function of MS‐DG pathway in depression is largely unknown. In the present study, we identified the MS‐DG pathway as a new circuit in regulating depressive‐like behaviors. The hM3Dq‐triggerd activation of MS‐DG projection neurons facilitated chronic stress‐induced depressive‐like behaviors. On the contrary, chronic silencing of the MS‐DG projection via hM4Di ameliorated CSDS‐induced depressive‐like behaviors. In particular, silencing of the MS^GABA+^‐DG projection exerted antidepressant effects. Altogether, our results identified a role of MS^GABA+^‐DG projection in stress response, and the stress‐induced depressive‐like behaviors could be attenuated by inhibition of MS‐DG projection.

Given that the inhibition of MS‐DG circuit could ameliorate depressive‐like behaviors, the molecular mechanism that reduce the activity of MS‐DG circuit was poorly understood. PDGF‐BB is a member of the PDGF family, which is widely expressed in the central nervous system and promotes the proliferation of neuronal precursors and generation of new dopaminergic neurons in both striatum and substantia nigra of rodents, which is similar to brain‐derived neurotrophic factor.^[^ ^36^ ^]^ In addition, long‐range GABAergic projection neurons from MS innervates PV interneurons in the DG to maintain the quiescence of NSCs and adult neurogenesis.^[^ ^6^ ^]^ In our study, the Gene Ontology analysis indicated that the functional role of PDGF signaling pathway was overlaped between depression and neurogenesis, providing a potential mechanism underlying the regulation of PDGF‐BB expression in DG by chemogenetic control of the activity MS‐DG projection neurons. The results from chemogenetic regulation suggest that hM4Di‐silencing and hM3Dq‐activation of MS‐DG pathway regulate the level of PDGF‐BB in the DG, supporting the hypothesis that MS neurons projecting to the DG as a putative upstream signal mediating PDGF‐BB expression in DG might be involved in the pathogenesis of depression. Furthermore, we confirmed that the expression of PDGF‐BB in SOM‐positive neurons was increased in CNO‐treated mice expressing hM4Di, revealing SOM‐positive neurons‐origin PDGF‐BB elevation in the DG by inhibition of MS^GABA+^‐DG projection. Previous studies have shown that PDGF‐BB is synthesized by several types of cells, including brain cells such as pericytes, neurons, microglia, and astrocytes,^[^ ^37^ ^]^ suggesting that the effect of MS‐DG circuit on PDGF‐BB signaling may also be produced on other types of cell besides neurons. Neurogenesis confers resilience to chronic stress by inhibiting the activity of mature granule cells in the ventral DG.^[^ ^38^ ^]^ Treatment of hippocampal slices or cultures with PDGF‐BB decreases the surface localization of NR2B and inhibits NR2B‐containing *N*‐methyl‐D‐aspartate receptor currents.^[^ ^39^ ^]^ In this way, PDGF‐BB signaling in the DG might prevent depressive‐like behaviors associated with the decline in the activity of hippocampal neurons. Both glutamatergic and cholinergic neurons send direct monosynaptic inputs to the hippocampus.^[^ ^40^ ^]^ Thus, understanding how SOM‐positive neurons in DG increased PDGF‐BB expression requires a full picture of the MS‐DG circuitry and remains an important topic for future investigation.

Depressed patients and chronically stressed animals have reduced hippocampal activity and volume as well as decreased expression of activity‐dependent genes and processes, including reduced adult neurogenesis in the hippocampus.^[^ ^41^ ^]^ In line with these observations, we showed that CSDS induced an inhibition of the proliferation and differentiation of NSCs, an imbalance between the aNSCs and qNSCs, and eventually a depletion of adult neurogenesis. Multiple lines of evidence suggest that PDGF‐BB is involved in the pathophysiology of neuropsychiatric and neurodegenerative disorders, including Alzheimer's disease,^[^ ^42^ ^]^ autism,^[^ ^43^ ^]^ and Parkinson's disease.^[^ ^13^ ^]^ Intranasal administration A1‐exosomes can prevent neurogenesis abnormalities and memory dysfunction accompanied by increased PDGF‐BB concentration in the hippocampus after status epilepticus,^[^ ^44^ ^]^ and PDGF‐BB is activated in the transplantation of mesenchymal stem cells, which alleviated schizophrenia‐like behavior and enhanced hippocampal neurogenesis.^[^ ^45^ ^]^ However, whether abnormal PDGF‐BB signaling contributes to depressive‐like behaviors remains largely unknown. In this study, we observed that the level of PDGF‐BB was decreased in the DG of defeated mice, which was consistent with clinical observation that bupropion‐plus‐escitalopram improves anhedonia, which in turn results in improved overall depression severity in depressed patients with elevated PDGF‐BB level.^[^ ^46^ ^]^ PDGF‐BB upregulation in the DG by delivery of AAV‐PDGF‐BB or pharmacological manipulations of PDGF‐BB inhibited depressive‐like behaviors and exerted beneficial effects in adult hippocampal neurogenesis in CSDS‐treated mice. Our results provided the direct evidence that PDGF‐BB mediated resilience to chronic stress. Meanwhile, PDGF‐BB signaling in the MS‐DG circuit was critical in modulating the behavioral response to stress, since infusion of PDGF‐BB‐neutralizing antibody directly into DG resulted in the decrease in the antidepressant action induced by inhibition of the MS‐DG pathway. Given these findings, in tandem with our data demonstrating that the inhibition of the MS‐DG circuit was sufficient to ameliorate depressive‐like behaviors, we deduce that PDGF‐BB signaling collapse within the MS‐DG circuit could represent a mechanistic link between stress exposure and impairment in hippocampal neurogenesis as well as stress‐induced depressive‐like behaviors.

Hippocampal neurogenesis is supported by radial glia‐like adult neural stem cells that are relatively quiescent, which is essential for maintenance of stem cell pools over long periods in the adult mammalian brain.^[^ ^47^ ^]^ Once qNSCs become activated and enter the cell cycle, they may self‐renew and generate proliferating NSCs (active NSCs, aNSCs) that subsequently differentiate into newborn dentate granule neurons and integrate into hippocampal neuronal circuits to become mature granule neurons.^[^ ^48^ ^]^ The balance between qNSCs and aNSCs is regulated by various factors and was involved in important cognitive functions such as memory and learning.^[^ ^49^ ^]^ Our results showed that the activation of adult NSCs was promoted by the overexpression of PDGF‐BB in DG, leading to the increased proliferation and differentiation of adult NSCs, and the dendritic branching, total length, and dendritic complexity deficits were reversed by PDGF‐BB, indicating that PDGF‐BB might be a regulator of neuronal maturation in adult hippocampal neurogenesis. More importantly, ablation of PDGFR*β* specifically in NSCs suppressed the activation and proliferation of NSCs in the DG, resulting in the prevention of PDGF‐BB‐induced antidepressant effects. Previous study has revealed that selective deletion of PDGFR*β* in NSCs of ventricular‐subventricular zone in the adult mice promotes qNSC activation,^[^ ^29^ ^]^ in contrast to deleting PDGFR*β* embryonically.^[^ ^50^ ^]^ Therefore, we could not completely exclude the possibility that PDGFR*β* knockout in other cell type such as pericytes at different stages of brain development may alter the function of PDGF‐BB. Our study shed light on the important role of PDGF‐BB in regulating adult hippocampal neurogenesis, promoting generation and maturation of newborn neurons, as well as a role of PDGFR*β*‐expressing NSCs‐related signaling pathway in maintaining NSCs in an active state without the depletion of NSCs pool.

Loss of PDGFR*β* compromised the maintenance of NSCs pool in the SGZ, leading to reduced neurogenesis and inducing depressive‐like behaviors. However, little is known about the related transcription factors and signaling pathways of PDGFR*β* in modulating hippocampal neurogenesis. According to the prediction of molecular interaction, we found that Kras, STAT3, STAT5 and other proliferation‐related genes were downstream target molecules of PDGFR*β*. It has been demonstrated that chronic fluoxetine treatment reverses the behavioral abnormalities, and prevents the inhibition of hippocampal neurogenesis induced by corticosterone treatment.^[^ ^51^ ^]^ On the other hand, the GSEA analysis showed that fluoxetine upregulated the level of JAK2, STAT3, Kras and promoted hippocampal neurogenesis. Previous study has found that treatment with PDGF‐BB restores neural function by regulating astrocytic PDGFR*β* signaling in STAT3‐dependent manner after subarachnoid hemorrhage.^[^ ^52^ ^]^ Moreover, the proliferation and differentiation of neural progenitor cells are differentially regulated by the JAK pathway.^[^ ^53^ ^]^ Our in vitro experiments showed that exposure of NSCs to PDGF‐BB resulted in increased JAK2‐STAT3 signaling. These observations suggest a possibility for the therapeutic potential of agonists of JAK‐STAT pathway for depressed patients. Further study is needed to investigate the neurogenesis‐related mechanism underpinning the JAK‐STAT pathway activator, for example, interferons,^[^ ^54^ ^]^ which is reported to be key mediators of astrogliogenesis in NSCs.^[^ ^55^ ^]^

**Figure 8:**
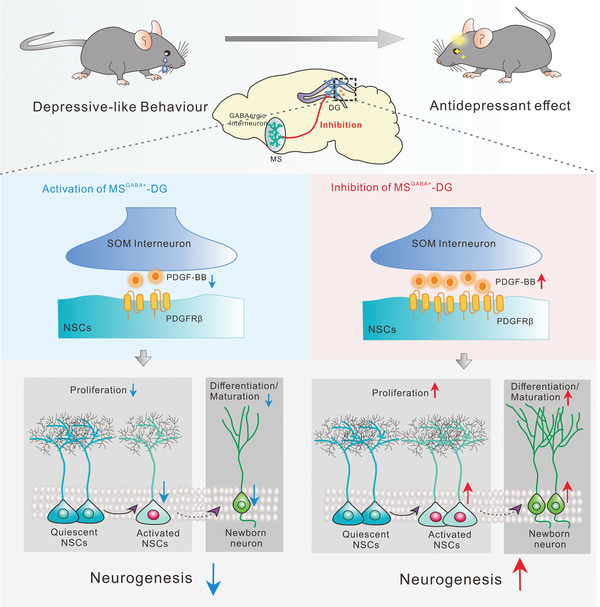
A model depicting the role of PDGF‐BB in the CSDS‐induced depressive‐like behavior. Chronic chemogenetic inhibition of MS‐DG projection neurons induced the elevation of PDGF‐BB in SOM‐positive interneurons in the DG, which enhanced adult hippocampal neurogenesis via acting on PDGFR*β* in the NSCs and alleviated CSDS‐induced depressive‐like behavior. On the contrary, activation of MS‐DG projection neurons induced depressive‐like behavior in the mice exposed to SSDS by decreasing the expression of PDGF‐BB in the DG, resulting in the impairment of adult hippocampal neurogenesis.

Together, we have found the functional connectivity in the MS^GABA+^‐DG circuit and its previously unidentified downstream molecule PDGF‐BB, which is capable of enhancing hippocampal neurogenesis via activating the JAK2/STAT3 pathway to mediate stress resilience in a PDGFR*β*‐dependent manner. Therefore, PDGF‐BB may be a critical link of hippocampal neurogenesis and depression, and a promising molecular target for the treatment of stress‐related psychiatric disorders (**Figure** **8**).

## 4 Experimental Section

### Mice

Male C57BL/6J mice (aged 6–8weeks) were obtained from the Animal Center of Hunan Silaike Jingda Laboratory Animal Corporation Ltd (Changsha, Hunan, China). Retired male CD‐1 mice were purchased from Vital River Laboratory Animal Technology Corporation Ltd. (Beijing, China). Vgat‐Cre mice and Nestin‐CreER^T2^ mice were obtained from in‐house breeders.^[^ ^56^ ^]^All mice were kept on a 12‐h light/dark cycle with ad libitum access to food and water. Male mice between 8 to 12 weeks of age were used for all behavioral tests. All procedures involving mice were approved by the Animal Welfare Committee of Huazhong University of Science and Technology and were in accordance with the National Institutes of Health Guide for the Care and Use of Laboratory Animals (No. [2020] IACUC Number: 3213).

### Stress Procedures

Chronic social defeat stress (CSDS) was carried out as previously described.^[^ ^17a^ ^]^ Briefly, C57BL/6J mice were placed in a resident home cage containing a novel CD‐1 aggressor for 10 min per day. After the physical defeat, C57BL/6J mice and CD‐1 mice were housed overnight in a two‐compartment cage and separated by a clear, perforated plexiglass allowing for sensory contact. This repeated stress was continued for 10 days, in which C57BL/6 mice faced a new aggressor each day. Control C57BL/6J mice were housed in same mouse cage with a different littermate for the length of stress.

The subthreshold stress defeat (SSDS) paradigm was performed as previously described.^[^ ^17a^ ^]^ The C57BL/6J mice were subjected to 3 sessions of 5‐min physically defeated by a CD‐1 aggressor. Then, the experimental mice were housed with the same aggressor CD‐1 mouse in a two compartments cage to allow the 15‐min sensory contact. At the end of the third session, stressed mice were single‐housed and subjected to behavior tests in the next day.

### Social Interaction Test

The two‐stage social interaction test was carried out with a novel CD1 mouse under dark conditions. Mice were placed into a novel, open‐field arena (42 × 42 × 42 cm) with an empty wire cage at one side (interaction zone). The movement of mice were tracked for 2.5 min with an empty cage (no target), and immediately followed by another 2.5 min in the presence of an unfamiliar CD‐1 mouse in the cage (target). The trajectory and movement of the mice were tracked using ANY‐maze software (Stoelting Co, USA). From these two stages, an SIT index (time in the interaction zone with CD‐1 mice/time in the interaction zone with empty cage) was calculated to classify mice into susceptible (ratio < 1) and resilient (ratio ≥ 1).

### Tail Suspension Test

Mice were individually suspended by the tail taped and roughly 20 cm above the floor. The video was recorded by ANY‐maze software. The immobility time (s) was manually measured during a 6‐min test period.

### Forced Swim Test

Mice were placed in a transparent cylinder (31 cm high, 24 cm diameter) filled with 24 ± 1 °C water to a depth of 20 cm and forced to swim for a 6‐min session. The duration time of immobility within the last 4 min of the 6‐min trial was measured by an experimenter blinded to the treatment groups.

### Sucrose Preference Test

The SPT was used to monitor anhedonia phenotype, a core symptom of depression. At the adaptation stage, the mice were given a choice between two identical bottles containing either drinking water or 1% sucrose for 24 h and the bottle locations were exchanged at 12 h. The amount of liquid consumption from each bottle was measured for 12 h after adaptation. The sucrose preference was calculated by determining the percentage of sucrose consumption/total liquid intake.

### Open Field Test

The OFT was performed by allowing mice to explore an open arena (42 cm width, 42 cm length, 42 cm height) for 10 min. The total distance was monitored by the ANY‐maze software (Stoelting CO, USA). And the total distance was used to assess the motor ability of mice. All animals were habituated to the testing room for 1 h before the behavioral test.

### Real‐Time Quantitative PCR

Total RNA was isolated from brain hippocampal dentate gyrus (DG) tissues using TRIzol reagent (Invitrogen, Carlsbad, CA) according to the manufacturer's instructions. RNA (500 ng) was then reverse transcribed into cDNA with RevertAid First Strand cDNA Synthesis Kit (Fermentas, Thermo Fisher Scientific Inc., Waltham, MA, USA) according to manufacturer protocols. The real‐time qPCR was performed in a StepOnePlus Real‐Time PCR System (Applied Biosystems, Foster City, CA) with SYBR Green PCR Master Mix (Applied Biosystems, Foster City, CA). Samples were heated to 95 °C for 10 min followed by 40 cycles of 95 °C for 15 s, 60 °C for 30 s, and 72 °C for 30 s. Levels of target gene mRNAs were calculated with the 2‐ΔΔCt method as normalized to GAPDH. The primer sequences used in real time PCR were listed in Table S1, Supporting Information.

### Western Blotting

Mice hippocampus DG tissues were homogenized in lysis buffer (RIPA) that contained protease (Roche, Basel, Switzerland) and phosphatase inhibitors (Sigma‐Aldrich, St. Louis, MO, USA) on ice for 30 min, and subsequently centrifuged at 12 000 rpm for 30 min at 4 °C. Supernatants were collected and the total protein samples were quantified by the bicinchoninic acid assay (Beyotime Biotechnology, Haimen, China). The protein supernatant was mixed with 3× loading buffer, and then deactivated in 95 °C for 10 min. 30 µg protein was separated by 8% SDS polyacrylamide gel and were transferred to nitrocellulose membranes (Millipore, Bedford, MA, USA). The immunoblots were incubated overnight at 4 °C with primary antibody: anti‐PDGFR*β* (#3169, 1:1000; Millipore, Bedford, MA, USA), anti‐JAK2 (#17670‐1‐AP, 1:1000; Proteintech, Wuhan, China), anti‐STAT3 (#10253‐2‐AP, 1:1000; Proteintech, Wuhan, China), *β*‐actin (#8480, 1:2000; Santa Cruz Biotechnology, California, USA). HRP‐labeled secondary antibodies were obtained from Thermo Scientific, Rockford, IL, and were used at a dilution of 1:5000. The densities of protein blots were obtained and analyzed using an odyssey Clx imaging system (LI−COR Biosciences).

### Enzyme Linked Immunosorbent Assay (ELISA)

The same process as above, and the hippocampus DG lysate was diluted for the required concentration. The platelet‐derived growth factor‐BB (PDGF‐BB) protein was measured using the PDGF‐BB ELISA system (EA‐2519; Signosis, Silicon Valley, USA) as previously published.^[^ ^44^ ^]^

### Primary Culture of Adult Hippocampal Neural Stem Cells In Vitro

NSCs were isolated from the young adult 8–10 weeks old DG, mechanically dissociated and enzymatically digested for 30 min at 37 °C in DMEM/F‐12 (#C11330500BT, Gibco, Grand Island, USA) media containing 10 mg mL^−1^ Papain (#LK003178, Worthington Biochemicals, Lakewood, USA), 20 mg mL^−1^ Dispase II (#04942078001, Sigma‐Aldrich, St. Louis, MO, USA), and 1 mg mL^−1^ DNase I (#DN25, Sigma‐Aldrich, St. Louis, MO, USA) based on published methods. Cells were then plated in Neurobasal media containing neurobasal (#21103‐049, Gibco, Grand Island, USA), supplemented with B27 (#17504‐044, Gibco, Grand Island, USA), l‐glutamic acid (#G3126, Sigma‐Aldrich, St. Louis, MO, USA), and penicillin streptomycin (#03‐031‐1BCS, Biological Industries, Kibbutz Beit‐Haemek, Israel). Media was changed every 2 days. About 4 weeks later, neurospheres would be formed and then passaged for further study. The PDGF‐BB (100 ng mL^−1^) was added in culture for 36 h. Characterization of neural stem/progenitor cell cultures was evaluated by immunofluorescent staining with the specific neural stem cell markers neuroepithelial stem cell protein (Nestin).

### Drug Treatments

For microinjection experiments, mice were bilaterally implanted with stainless steel guide cannulas in DG (AP, 2.0 mm, ML, ±1.5 mm, DV, 2.1 mm) or lateral ventricle (AP, 0.3 mm, ML, ±1.0 mm, DV, −2.5 mm). Recombinant PDGF‐BB protein (R&D systems, Minnesota, USA) was dissolved in cell PBS and infused into DG or lateral ventricle with a volume of 4 or 2 µL per side. Anti‐PDGF‐BB monoclonal antibody (R&D systems, Minnesota, USA) or IgG isotype control (Santa Cruz Biotechnology, California, USA) was infused into DG at a dose of 4 µg per mouse in 100 µL sterile saline.

For bromodeoxyuridine (BrdU) labeling, BrdU (Sigma, St Louis, MO, USA) was diluted in sterile 0.9% NaCl solution to make a 5 mg mL^−1^ stock solution and intra‐peritoneal injection at a dose of 50 mg kg^−1^ for 3 days with a 24 h interval between each injection. Twenty‐four hours after the last BrdU injection, the mice were perfused with 0.9% saline followed by 4% paraformaldehyde as described below.

To induce transgene expression in NSCs, tamoxifen (Sigma, St Louis, MO, USA) was dissolved at a concentration of 10 mg mL^−1^ in ethanol and sunflower seed oil under constant agitation at room temperature. To induce recombination, 8‐week‐old animals were intraperitoneally injected with 75 mg kg^−1^ of tamoxifen or vehicle every 24 h for five consecutive days per animal.

For the chemogenetic test, Clozapine‐N‐oxide (CNO) was purchased from MCE (MedChemExpress, New Jersey, USA). After four weeks allowing for virus expression, mice received an intraperitoneal injection of 5 mg kg^−1^ body weight CNO that was diluted in 0.9% saline solution to yield a final dimethyl sulfoxide concentration of 0.5% for or a local infusion of CNO (1 mm, 2 µL, 5 days) into the DG for 1 h before the behavioral test and biochemical analyses. Saline solution for control injections also consisted of 0.5% DMSO. Animals were monitored after recovery and 4 weeks allowed to pass after the final injection before any analyses were performed.

### Stereotaxic Microinjection

The mice were anesthetized with sodium pentobarbital (40 mg kg^−1^) and then attached to a stereotaxic apparatus. For in vivo viral injection, 300 nL adeno‐associated virus (AAV) or 800 nL lentivirus (LV) was injected into the medial septum (MS) (AP, 0.75 mm, ML, 0 mm, DV, 3.75 mm) or DG with glass capillaries with tip resistance of 5–10 MΩ using a pressure microinjector (Micro 4 system, World Precision Instruments). The needle syringe was removed 10 min after the end of the injection. Virus used in this study were listed in in Table S3, Supporting Information. For retrograde tracing studies, mice were received unilateral infusion of 300 nL cholera toxin subunit B (CTB) conjugated with Alexa 555 (CTB‐555, BrainVTA, Wuhan, China) into DG to allow observation of projecting specific neurons.

### Immunofluorescent Staining

Mice were anesthetized with 1% pentobarbital sodium and perfused intracardially with 0.9% saline followed by 4% paraformaldehyde in PBS. The brains were post‐fixed in paraformaldehyde for 12 h, and then cryoprotected in 15% and 30% sucrose solutions for 2 days at 4 °C. Fixed brains were sectioned with a freezing microtome (CM1900, Leica Microsystems, Wetzlar, Germany) at 30 µm.

For immunofluorescence staining, the tissue sections were washed in PBS, blocked in a buffer containing 5% bull serum albumin and 0.3% Triton X‐100 for 1 h, followed by incubation with primary antibodies diluted in blocking buffer for 24 h at 4 °C. After washing three times, sections were incubated with secondary antibodies for 2 h at 37 °C.

For 5‐bromo‐2‐deoxyuridine (BrdU) staining, sections were incubated in 1 m hydrochloric acid (HCl) for 10 min and then 2 m HCl for 10 min at room temperature and additional 20 min at 37 °C. Next, sections were neutralized in 0.1 m sodium borate (pH 8.5), blocked in 5% BSA and 0.3% Triton X‐100 in phosphate buffer for 1 h, and incubated overnight in primary antibodies in the same blocking buffer. Immunofluorescent detections were performed with secondary antibodies. Antibodies were used as follows: Primary antibodies used were rat anti‐BrdU (#ab6326, 1:1000; Abcam, Cambridge, MA, USA), mouse anti‐glial fibrillary acidic protein (GFAP, #3670, 1:1000; Cell Signaling Technology, Denvars, MA, USA), rabbit anti‐ platelet‐derived growth factor receptor‐beta (PDGFR*β*, #ab32570, 1:300; Abcam, Cambridge, MA, USA), mouse anti‐Nestin (#MAB353, 1:200; Millipore, Bedford, MA, USA), rabbit anti‐ SRY‐related high‐mobility‐group (HMG)‐box protein‐2 (Sox2, #ab97959, 1:1000; Abcam, Cambridge, MA, USA), rabbit anti‐Doublecortin (DCX, #4604, 1:500; Cell Signaling Technology, Denvars, MA, USA), mouse anti‐GAD67 (#ab26116, 1:200; Abcam, Cambridge, MA, USA), rabbit anti‐c‐Fos (#ab190289, 1:1000; Abcam, Cambridge, MA, USA), mouse anti‐cholecystokinin (CCK, #ab37274, 1:200; Abcam, Cambridge, MA, USA), mouse anti‐CamkII‐*α* (#50049, 1:200; Cell Signaling Technology, Denvars, MA, USA), guinea pig anti‐ somatostatin (SOM, #366004, 1:200; Synaptic System, Göttingen, Germany), mouse anti‐ vasoactive intestinal peptide (VIP, #25347, 1:200; Santa Cruz Biotechology, California, USA), rabbit anti‐ PDGF‐BB (#ab178409, 1:100; Abcam, Cambridge, MA, USA), rabbit anti‐CTB (#pab13910, 1:400; Abnova, Taipei City, China ). Fluorescent secondary antibodies used: donkey anti‐rat Alexa Fluor 488 (#A21208, 1:500; Invitrogen, Thermo Fisher Scientific Inc, Waltham, MA, USA), donkey anti‐rabbit Alexa Fluor 488 (#A21206, 1:500; Invitrogen, Thermo Fisher Scientific Inc, Waltham, MA, USA), donkey anti‐rat Alexa Fluor 594 (#A21209, 1:500; Invitrogen, Thermo Fisher Scientific Inc, Waltham, MA, USA), donkey anti‐rabbit Alexa Fluor 594 (#A21207, 1:500; Invitrogen, Thermo Fisher Scientific Inc, Waltham, MA, USA), donkey anti‐mouse Alexa Fluor 647 (#A32787, 1:500; Invitrogen, Thermo Fisher Scientific Inc, Waltham, MA, USA), donkey anti‐rat Alexa Fluor 647 (#712‐605‐153, 1:500; JacksonImmuno Research, West Grove, Pennsylvania, USA), goat anti‐mouse Alexa Fluor 405 (#ab175660, 1:500; Abcam, Cambridge, MA, USA), 4′,6‐diamidino‐2‐phenylindole dihydrochloride (DAPI, #B2261, 1:10000; Sigma, St Louis, MO, USA) was used for counterstaining. Fluorescent images were obtained by a confocal microscope (FV1000, Olympus, Tokyo, Japan).

### Quantification and Dendritic Morphology Analysis

Stereological quantification of cells was performed as previously described with a slight modification.^[^ ^57^ ^]^ Briefly, BrdU positive cells were counted in a one‐in‐ten series of 30‐µm sections (6 sections per mouse) through hippocampus and multiplied by ten to indicate the total number of cells per dorsal DG. To calculate the number of cells showing colocalization of BrdU and other markers (DCX, Sox2, or GFAP), using the same method as above in constrained sections. To quantify the number of c‐Fos positive cells in the DG, every tenth brain slice in four sections from each mouse was selected, and each group contained three mice. The sum of the c‐Fos positive cells was multiplied by ten to estimate the total number of cells per DG.

For the analysis of dendritic morphology, all ROV‐GFP‐labeled newborn neurons with intact dendritic branches were selected for total dendritic length and branch number by Sholl analysis by counting the number of dendrites that crossed a series of concentric circles at 20 µm intervals from the cell soma.

### Electrophysiological Slice Recordings

For recordings in the MS, acute brain slices from mice previously injected with the retrograde virus AAV‐hSyn–Cre into the DG and AAV‐DIO‐hM3Dq/hM4Di‐mCherry into the MS were used. Brain slices were prepared as described previously.^[^ ^58^ ^]^ Briefly, animals were deeply anesthetized using isoflurane and transcardially perfused using ice‐cold high sucrose solution saturated with 95% O_2_/5% CO_2_ consisting of the following (in mM): 210 sucrose, 3.1 sodium pyruvate, 1.0 NaH_2_PO_4_, 11.6 sodium l‐ascorbate, 5.0 MgCl_2_, 26.2 NaHCO_3_, and 20.0 glucose, pH 7.4. Brains were removed from the skull and coronal slices were sectioned at 300 µm thickness with a vibratome (VT1000 S, Leica, Wetzlar, Germany). Slices were then immediately transferred and held in a modified artificial cerebrospinal fluid (ACSF), consisting of the following (in mm): 119.0 NaCl, 4.7 KCl, 1.0 NaH_2_PO_4_‐2H_2_O, 1.3 MgSO_4_, 2.5 CaCl_2_, 26 NaHCO_3_, 10.0 glucose, for recovery until being transferred to a recording chamber. Whole‐cell voltage‐clamp recordings of mCherry^+^ cells in the MS (medial septum) were made using patch electrodes (3–6 MΩ resistance) filled with intracellular solution containing (in mm): 140.0 K‐gluconate, 3.0 KCl, 2.0 MgCl_2_, 10.0 HEPES, 0.2 EGTA, and 2.0 Na‐GTP (adjusted to 280–290 mOsm and pH 7.2). Recordings were initiated 5 min after the loose‐seal cell‐attached recordings were established. For hM4Di/hM3Dq‐mediated inhibition/activation of the MS‐DG pathway, CNO (10 µm) was added to the circulating aCSF after 2–5 min of baseline recordings. Action potential firing frequency from MS area were monitored following bath application of CNO for 10 min. All data were acquired and analyzed using pCLAMP 10 software (Axon Instruments, Molecular Devices) and Mini Analysis Program (Synaptosoft, Decatur, GA, USA).

### Statistical Analysis

Data values were expressed as the group mean ± SEM. Student's *t‐*test was used to compare the differences between two groups; one‐way analysis of variance (ANOVA) followed by Tukey's post hoc test was used for multiple group comparisons. Two‐way ANOVA followed by the Bonferroni's post hoc test was used was used for two group comparisons. All statistical analyses were performed by using Prism version 8.0 software GraphPad. Error bars indicate standard error of the mean. *p* < 0.05 was considered significant. The statistical analyses and sample size applied for each experiment were indicated in the figure legends. Details are provided in Table S4, Supporting Information.

## Conflict of Interest

The authors declare no conflict of interest.

## Author Contributions

H.‐H.L. and Y.L. contributed equally to this work. All studies were conceptualized and designed by J.‐G.C. and F.W. The behavioral tests and immunofluorescence experiments were performed and analyzed by H.‐H.L. and Y.L. The electrophysiology recordings and western blotting were performed and analyzed by H.‐S.C. The in vitro experiments were performed by Y.‐K.L. and R.S. The qPCR examination was performed and analyzed by Y.Z. The paper was written by H.‐H.L. and revised by H.‐S.C., F.W., and J.‐G.C.

## Supporting information

Supporting Information

## Data Availability

The data that support the findings of this study are available from the corresponding author upon reasonable request.
