# Supplementary material for: PDGF‐BB‐Dependent Neurogenesis Buffers Depressive‐Like Behaviors by Inhibition of GABAergic Projection from Medial Septum to Dentate Gyrus
Source: Adv Sci (Weinh). 2023 Jun 16;10(22):2301110. doi: 10.1002/advs.202301110 (PMC10401107; doi:10.1002/advs.202301110)
Supplement: Supplementary file 1 — Supporting Information [file ADVS-10-2301110-s001.pdf]

ADVANCED SCIENCE
Open Access

Supporting Information

for *Adv. Sci.*, DOI 10.1002/advs.202301110

PDGF-BB-Dependent Neurogenesis Buffers Depressive-Like Behaviors by Inhibition of GABAergic Projection from Medial Septum to Dentate Gyrus

Hou-Hong Li, Yang Liu, Hong-Sheng Chen, Ji Wang, Yu-Ke Li, Yang Zhao, Rui Sun, Jin-Gang He, Fang Wang and Jian-Guo Chen**

Supporting information

PDGF-BB-Dependent Neurogenesis Buffers Depressive-Like Behaviors by Inhibition of GABAergic Projection from Medial Septum to Dentate Gyrus

Hou-Hong Li[1], Yang Liu[1], Hong-Sheng Chen[1,2], Ji Wang[1], Yu-Ke Li[1], Yang Zhao[1], Rui Sun[1], Jin-Gang He[1,2,3,4], Fang Wang[1,2,3,4,*], Jian-Guo Chen[1,2,3,4,*]

Supplementary Figures

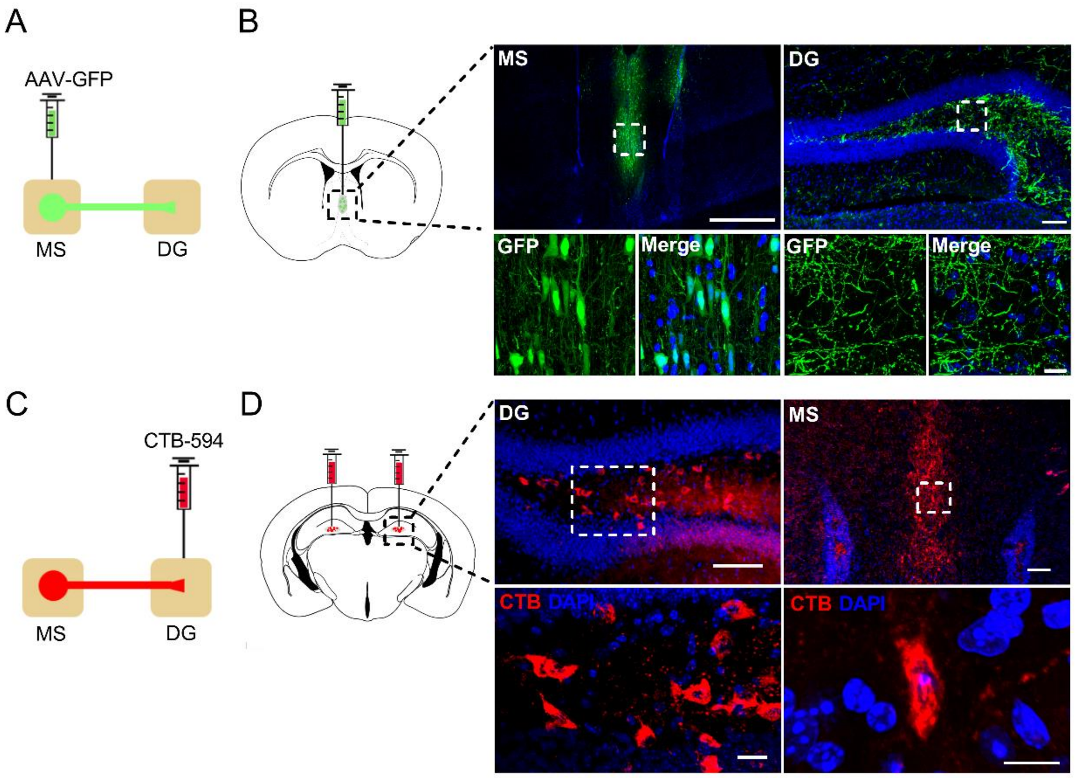

Suppl. Fig. 1 Monosynaptic connection of MS neurons to DG neurons. (A) Schematic of virus injections strategy. **(B)** Images showing GFP expression in the MS (left, green) and GFP-labeled MS axons in the DG (right, green). Top: Scale bar = 1000 µm (left), 100 µm (right). Below: Scale bar = 20 µm. **(C)** Schematic of CTB injections strategy. **(D)** Images showing CTB expression in the DG (left, red) and retrograde labeled neurons in the MS (right, red). Left: Scale bar = 1000 µm (top), 100 µm (below). Right: Scale bar = 100 µm (top), 10 µm (below).

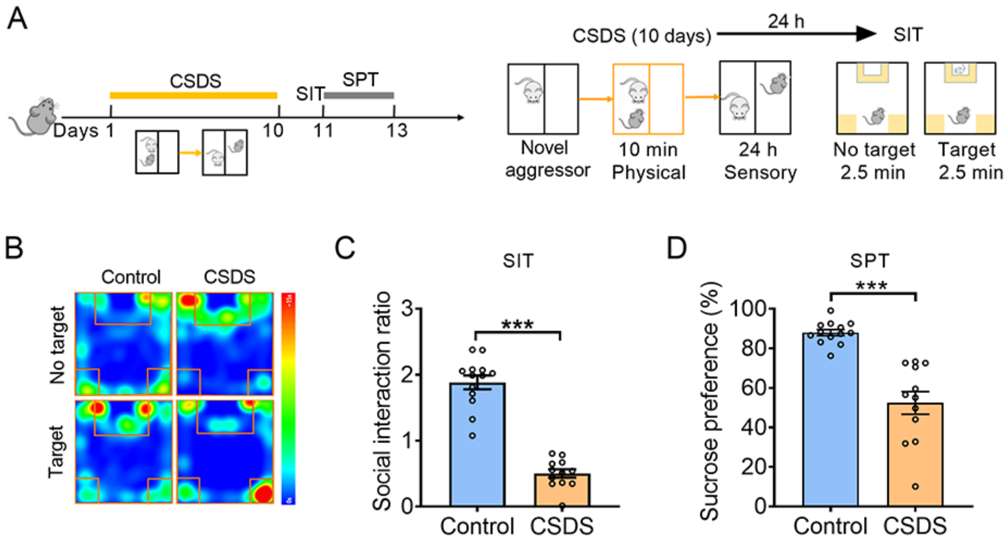

Suppl. Fig. 2 Chronic social defeat stress induces depressive-like behaviors in mice. **(A)** Experimental timeline and behavioral paradigm of CSDS experiments. **(B)** Representative heatmaps of social avoidance behavioral test. **(C)** Depressive-like behaviors in stressed mice, as measured by the social interaction ratio in SIT after CSDS. n = 12-13 per group. **(D)** Depressive-like behaviors in stressed mice, as measured by the sucrose preference in SPT after CSDS. n = 12-13 per group. Data are expressed as mean ± SEM. Student's *t* test (C, D). ***$P < 0.001$. The statistical details can be found in Supplementary Table S4.

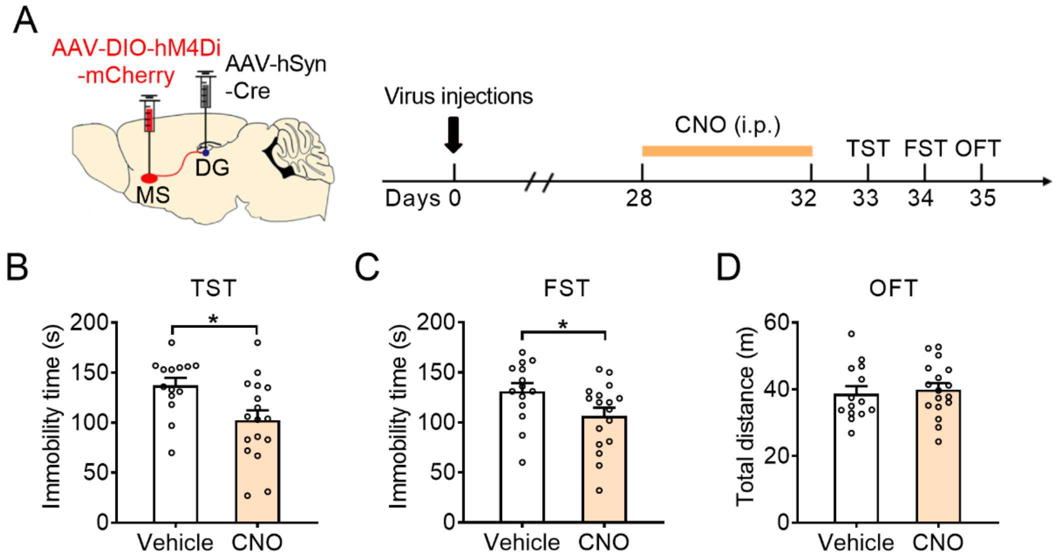

Suppl. Fig. 3 Chemogenetic inhibition of MS-DG projections produces antidepressant effect in naïve mice. (A) Schematic of the stereotaxic injections for selective expression of the hM4Di mCherry in MS-DG projection neurons. **(B)** Behavioral effects of the chemogenetic inhibition of MS-DG projection neurons in the TST. n = 14-17 per group. **(C)** Behavioral effects of the chemogenetic inhibition of MS-DG projection neurons in the FST. n = 14-17 per group. **(D)** Behavioral effects of the chemogenetic inhibition of MS-DG projection neurons in the OFT. n = 14-17 per group. Data are expressed as mean ± SEM. Student's *t* test (B-D). $*P < 0.05$. The statistical details can be found in Supplementary Table S4.

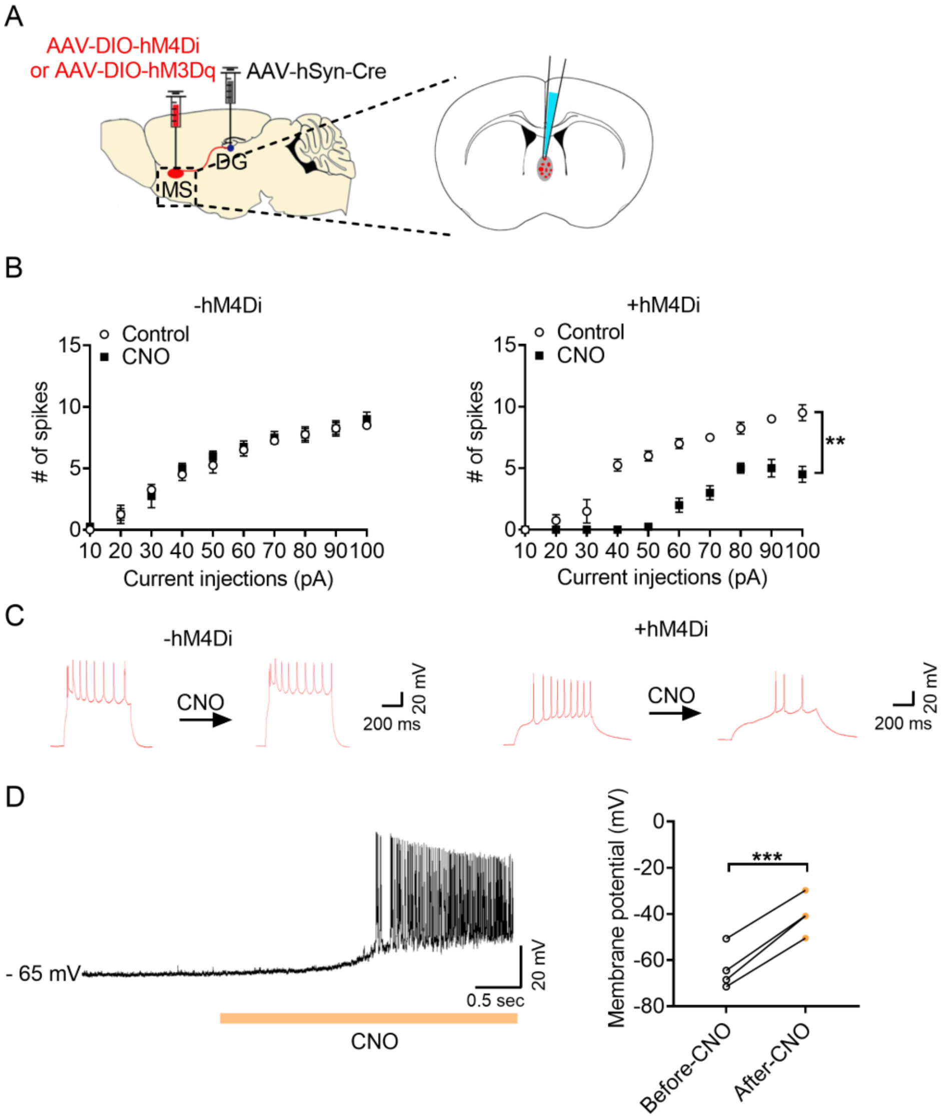

Suppl. Fig. 4 Electrophysiological recording of MS neurons after exogenous expression DREADDs with bath application of CNO. (A) Schematics showing viral vector AAV-hM4Di(hM3Dq)-mCherry infusion in MS and patch-clamp recordings from DG-projecting MS neurons. **(B)** Electrophysiological recording showed the firing of MS neurons expressing hM4Di-mCherry in the presence of 10 μM CNO. n = 4 cells from three mice per group. **(C)** Patch-clamp recording from neurons expressing hM4Di-mCherry in the MS with representative trace of CNO-induced action potential firing in neurons expressing hM4Di-mCherry. **(D)** Patch clamp recording from neurons expressing hM3Dq-mCherry in the MS with representative trace of CNO-induced firing of action potential in neurons expressing hM3Dq-mCherry. Right: Summary of change

in resting membrane potential following bath application of CNO. n = 4 cells from three mice per group. Data are expressed as mean ± SEM. Student's *t* test (B, D). $^{**}P < 0.01$, $^{***}P < 0.001$. The statistical details can be found in Supplementary Table S4.

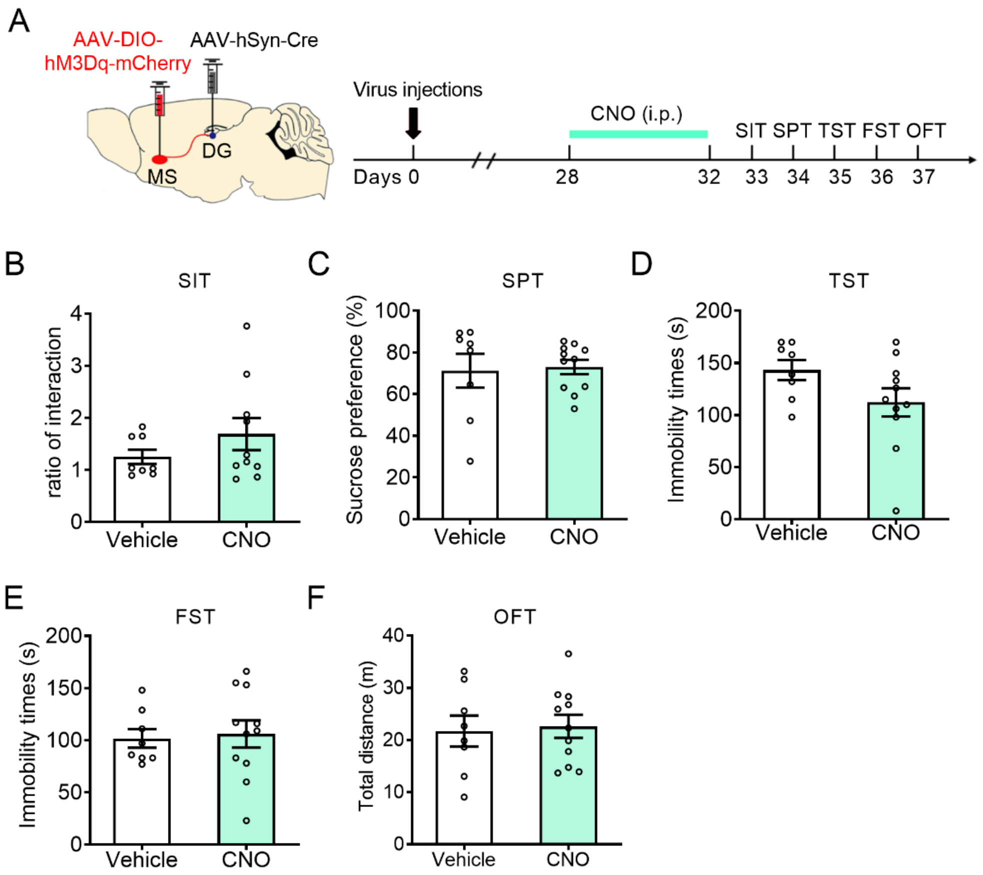

Suppl. Fig. 5 Chemogenetic activation of MS-DG projections has no effects on depressive-like behaviors in naïve mice. (**A**) Schematic of the stereotaxic injections for selective expression of the hM3Dq-mCherry in MS-DG projection neurons. (**B**) Behavioral effects of the chemogenetic activation of MS-DG projection neurons in the SIT. n = 8-10 per group. (**C**) Behavioral effects of the chemogenetic activation of MS-DG projection neurons in the SPT. n = 8-11 per group. (**D**) Behavioral effects of the chemogenetic activation of MS-DG projection neurons in the TST. n = 8-11 per group. (**E**) Behavioral effects of the chemogenetic activation of MS-DG projection neurons in the FST. n = 8-11 per group. (**F**) Behavioral effects of the chemogenetic activation of MS-DG projection neurons in the OFT. n = 8-11 per group. Data are expressed as mean ± SEM. Student's *t* test (B-F).The statistical details can be found in Supplementary Table S4.

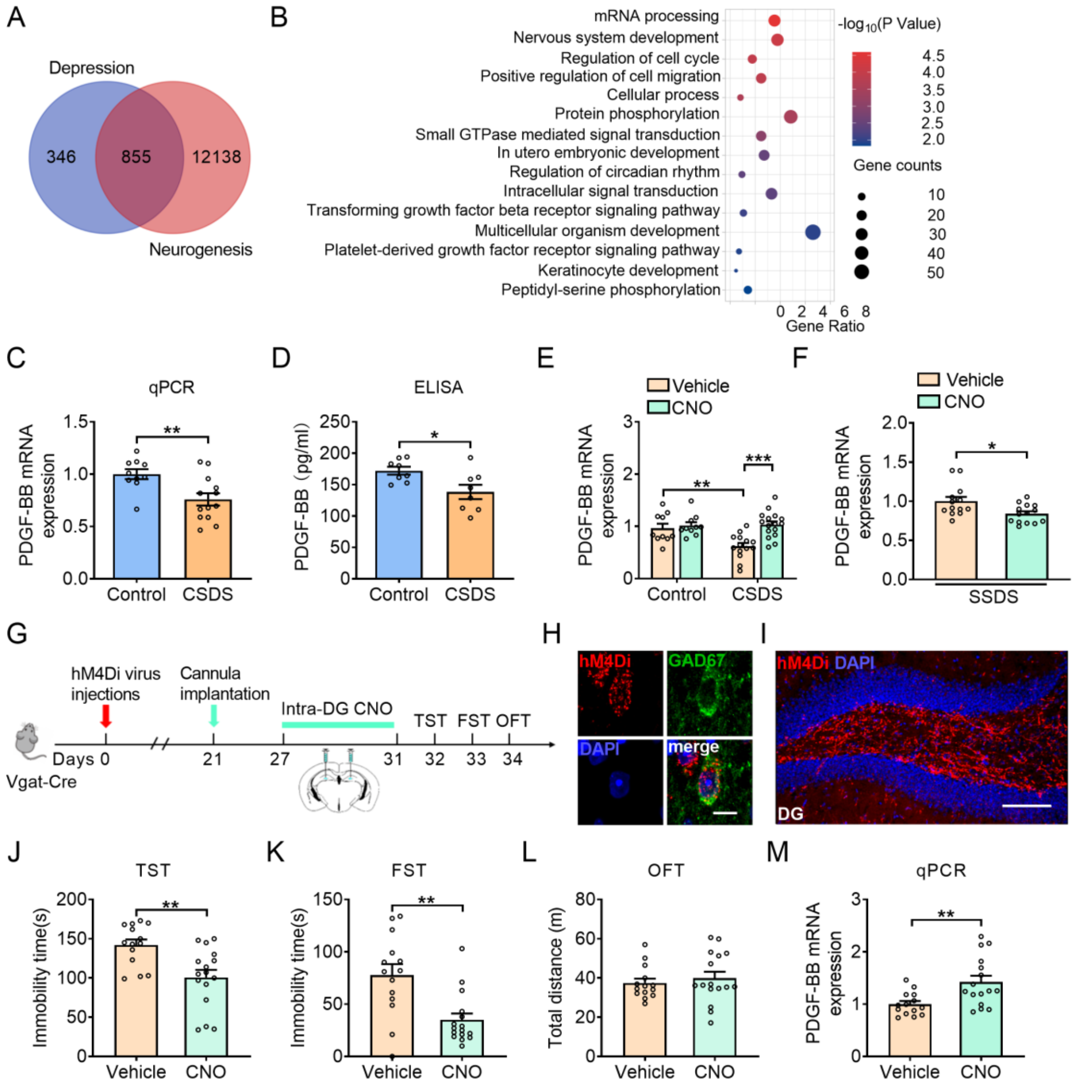

Suppl. Fig. 6. Inhibition of MS^{GABA+}-DG projection induces the expression of PDGF-BB and prevents depression-like behaviors. (**A**) Venn diagram showing the overlap of DEGs in the depression and neurogenesis samples. (**B**) KEGG analysis of the 855 genes. (**C**) PDGF-BB mRNA expression in the DG after CSDS. n = 10-13 per group. (**D**) PDGF-BB protein levels in the DG as determined by ELISA after CSDS. n = 8 per group. (**E**) PDGF-BB mRNA expression in the DG after inhibition of MS-DG projection. n = 10-16 per group. (**F**) PDGF-BB mRNA expression in the DG after activation of MS-DG projection. n = 13-14 per group. (**G**) Timeline of experimental procedure. (**H**) Representative images showed that hM4Di-mCherry labeled neurons in the MS expressing GAD67. Scale bars: 10 μm. (**I**) Confocal fluorescent image showing the distribution of GABAergic axon fibers, which expressed hM4Di-mCherry and

innervated hippocampal DG regions. Scale bars: 100 μm. (**J**) Effects of chemogenetic inhibition of MS-DG GABAergic neurons on the immobility time in TST. n = 14-16 per group. (**K**) Effects of chemogenetic inhibition of MS-DG GABAergic neurons on the immobility time in FST. n = 14-16 per group. (**L**) Effects of chemogenetic inhibition of MS-DG GABAergic neurons on the locomotor activity in the OFT. n = 14-16 per group. (**M**) PDGF-BB mRNA expression in the DG after inhibition of MS^{GABA+}-DG projection in the Vgat-Cre mice. n = 14-16 per group. Data are expressed as mean ± SEM. Student's *t* test (C, D, F, J-M). Two-way ANOVA followed by the Bonferroni's post hoc test (E). $*P < 0.05$, $**P < 0.01$, $***P < 0.001$. The statistical details can be found in Supplementary Table S4.

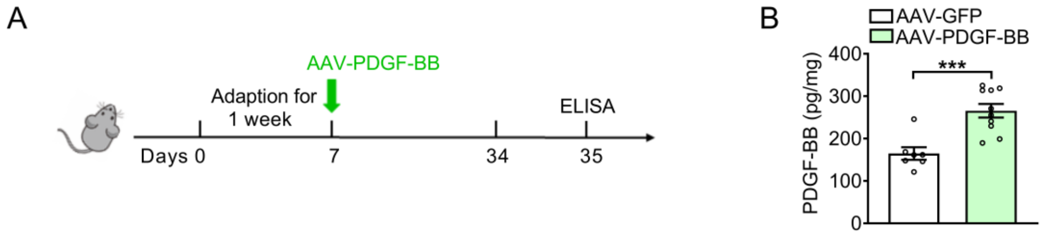

Suppl. Fig. 7 PDGF-BB overexpression in DG after virus injection in naïve mice. (**A**) Experimental timeline of PDGF-BB overexpression in the DG and behavioral test. (**B**) PDGF-BB protein levels in the DG as determined by ELISA four weeks after injection with AAV-GFP or AAV-PDGF-BB. n = 7-10 per group. Data are expressed as mean ± SEM. Student's *t* test. ***$P < 0.001$. The statistical details can be found in Supplementary Table S4.

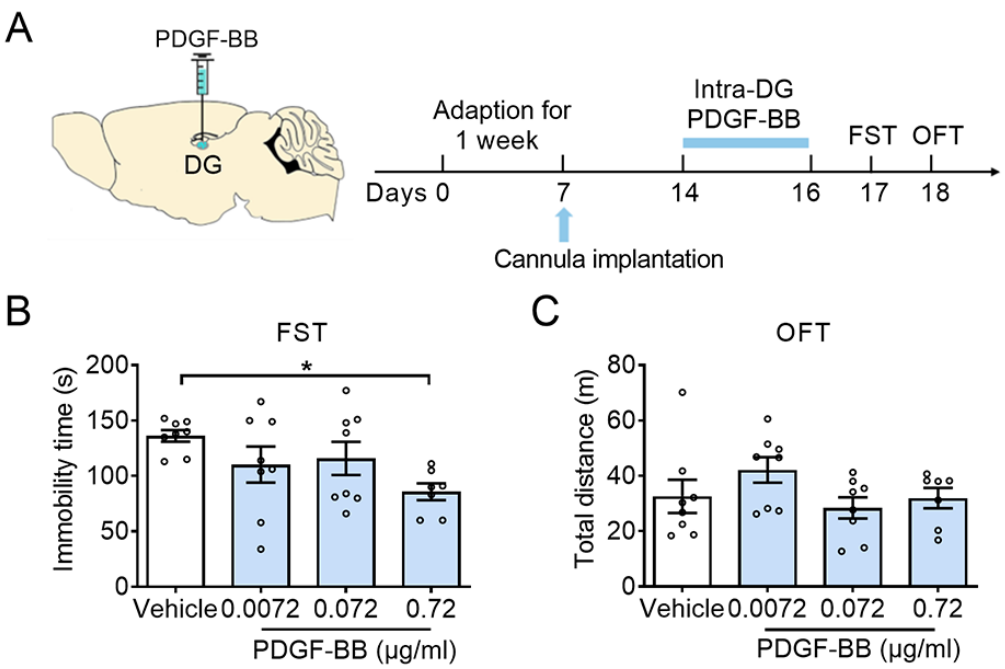

Suppl. Fig. 8 The antidepressant effects of local injection of PDGF-BB into the DG. (**A**) Experimental timeline for behavioral testing after intra-hippocampal infusion of vehicle and PDGF-BB. (**B**) Time spent immobile in the FST following vehicle or PDGF-BB treatment. n = 7-8 per group. (**C**) Locomotor activity measured by the total distance traveled by the naïve mice with vehicle or PDGF-BB treatment. n = 7-8 per group. Data are expressed as mean ± SEM. One-way ANOVA followed by Tukey's post hoc test (B, C). *$P < 0.05$. The statistical details can be found in Supplementary Table S4.

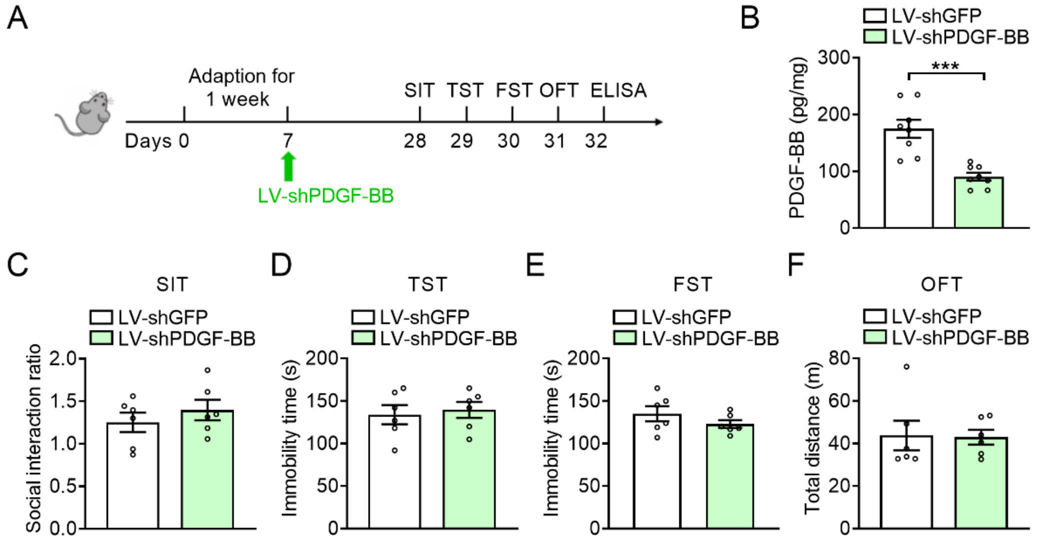

Suppl. Fig. 9 Knockdown of PDGF-BB in the DG of naïve mice does not induce depressive-like phenotypes. (**A**) Experimental timeline of PDGF-BB knockdown in the DG and behavioral test. (**B**) PDGF-BB protein levels in the DG as determined by ELISA three weeks after injection with LV-shGFP or LV-shPDGF-BB. n = 8 per group. (**C**) Behavioral effects of PDGF-BB knockdown in the SIT. n = 6 per group. (**D**) Behavioral effects of PDGF-BB knockdown in the TST. n = 6 per group. (**E**) Behavioral effects of PDGF-BB knockdown in the FST. n = 6 per group. (**F**) Behavioral effects of PDGF-BB knockdown in the OFT. n = 6 per group. Data are expressed as mean ± SEM. Student's *t* test (B-F). ***P < 0.001. The statistical details can be found in Supplementary Table S4.

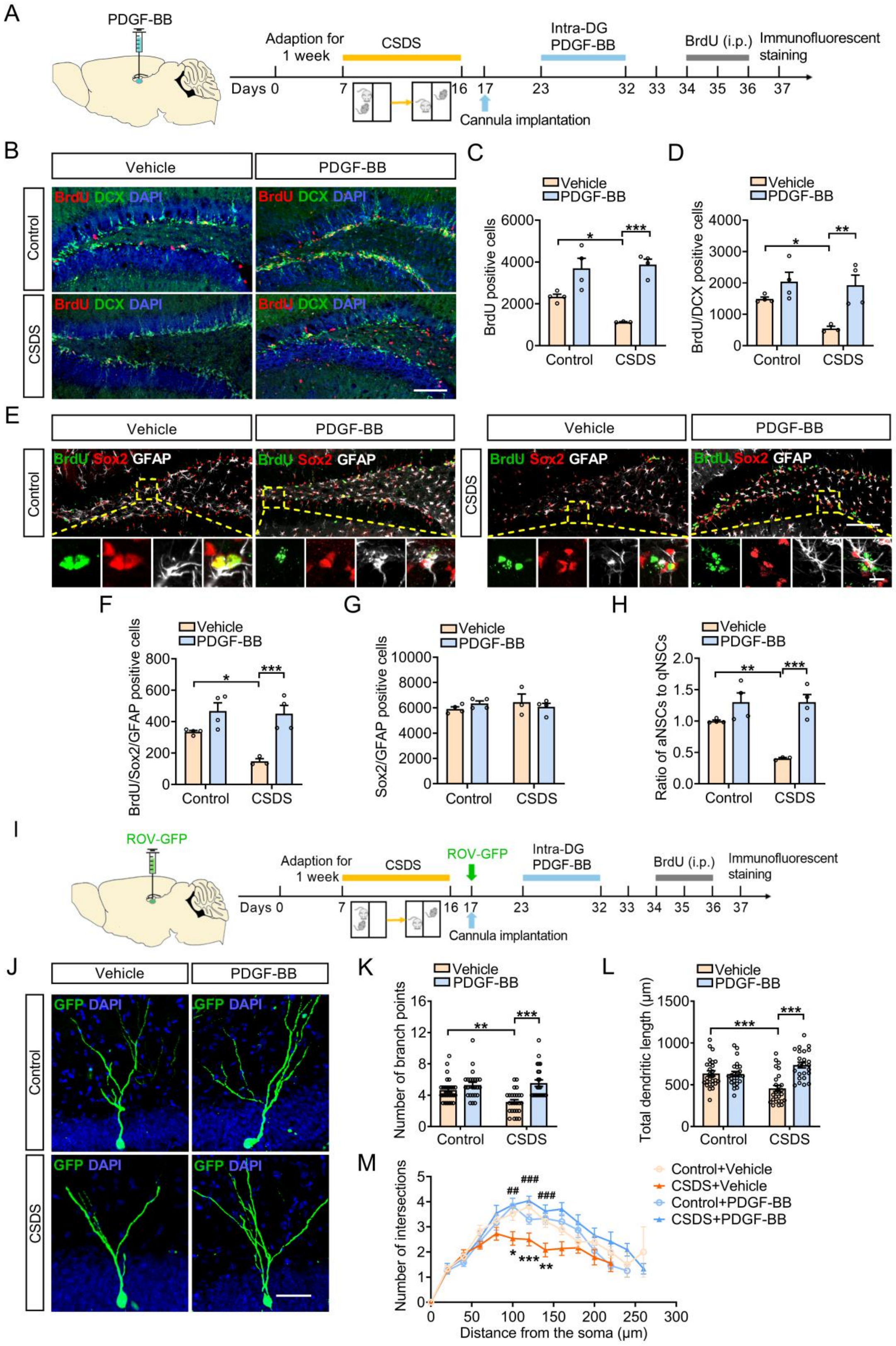

Suppl. Fig. 10 Treatment with PDGF-BB reverses CSDS-induced alteration of hippocampal neurogenesis in the DG. (**A**) Experimental timelines for CSDS protocol, PDGF-BB treatment, and the BrdU injection protocol. (**B**) Immunofluorescence images

for BrdU (red), DCX (green) within the SGZ of the hippocampus in control or CSDS-exposed mice treated with vehicle or PDGF-BB. Scale bars: 100 μm. (**C**) Quantification of the number of BrdU positive cells in SGZ of the hippocampus after PDGF-BB treatment. n = 3-4 per group. (**D**) Quantification of the number of BrdU/DCX positive cells in SGZ of the hippocampus after PDGF-BB treatment. n = 3-4 per group. (**E**) Immunofluorescence images for BrdU (green), Sox2 (red), and GFAP (white) within the SGZ of the hippocampus in control or defeated mice treated with vehicle or PDGF-BB. Panels on the below are magnified images showing that the colocalization of BrdU with GFAP and Sox2. Scale bar: 100 μm (top), 10 μm (below). (**F**) Quantification of the number of BrdU/Sox2/GFAP positive NSCs in SGZ of the hippocampus after PDGF-BB treatment. n = 3-4 per group. (**G**) Quantification of the number of Sox2/GFAP positive NSCs in SGZ of the hippocampus after PDGF-BB treatment. n = 3-4 per group. (**H**) The ratio of aNSCs to qNSCs. n = 3-4 per group. (**I**) Experimental timelines for CSDS protocol, PDGF-BB treatment, and the virus injection protocol. (**J**) Representative images of ROV-GFP labeled newborn neurons in vehicle- or PDGF-BB-treated mice 4 weeks after retrovirus injection. Scale bars: 50 μm. (**K**) Quantification of branch number of ROV-GFP labeled newborn neurons in vehicle- or PDGF-BB-treated mice 4 weeks after retrovirus injection. n = 24-27 neurons per group. (**L**) Quantification of dendritic length of ROV-GFP labeled newborn neurons in vehicle- or PDGF-BB-treated mice 4 weeks after retrovirus injection. n = 24-27 neurons per group. (**M**) Sholl analysis of dendritic complexity of ROV-GFP labeled newborn neurons in vehicle- or PDGF-BB-treated mice 4 weeks after retrovirus injection. n = 24-27 neurons per group. Data are expressed as mean ± SEM. Two-way ANOVA followed by the Bonferroni's post hoc test (C, D, F-H, K-M). $^{*}P < 0.05$; $^{**}P < 0.01$; $^{***}P < 0.001$. $^{\#\#}P < 0.01$, $^{\#\#\#}P < 0.001$ vs CSDS + Vehicle.The statistical details can be found in Supplementary Table S4.

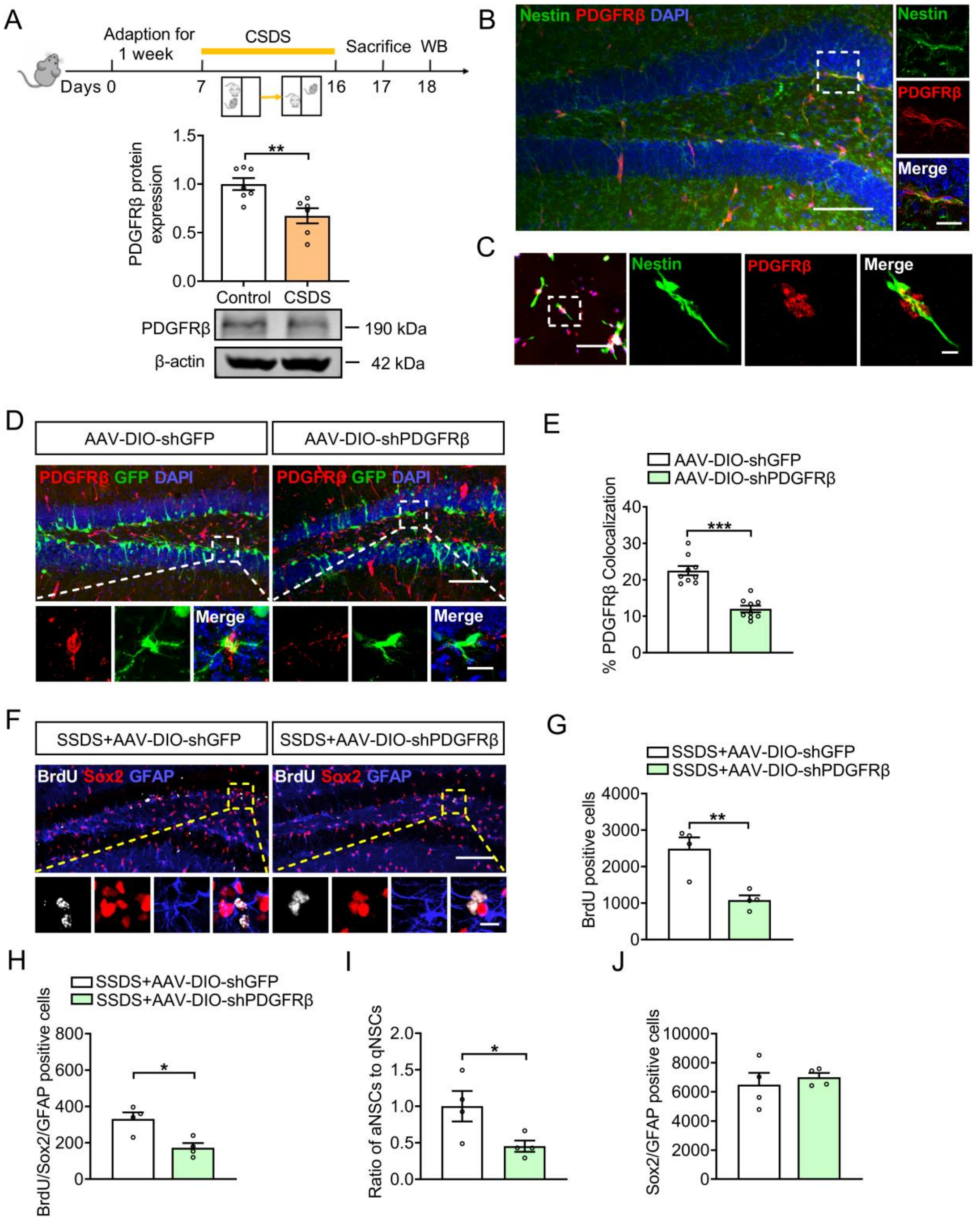

Suppl. Fig. 11 Deletion of PDGFRβ from NSCs impairs hippocampal neurogenesis. (**A**) Experimental timeline of CSDS experiments and the change in levels of PDGFRβ protein in the DG after CSDS. n = 6-7 per group. (**B**) Double-labeled confocal immunofluorescence images showing the colocalization of PDGFRβ (red) expression with Nestin (green). Top images: Scale bar =100 μm, Bottom images: Scale bar = 20 μm. (**C**) Immunohistochemistry for Nestin (green), PDGFRβ (red) nuclear counterstaining with DAPI (blue). Left images: Scale bar = 100 μm. Right images:

Scale bar = 10 µm. (**D**) Representative confocal micrographs of DG from control mice (left) or mice injected with AAV-DIO-shPDGFRβ (right), showing transduced cells marked by GFP (green) and PDGFRβ protein detected by immunolabeling (red). Top images: Scale bar =100 µm, Bottom images: Scale bar =20 µm. (**E**) Quantification of PDGFRβ neurons colocalized with GFP. n = 9 slices per group. (**F**) Immunohistochemistry for BrdU (white), Sox2 (red), and GFAP (blue) within the SGZ of the hippocampus in AAV-DIO-shGFP or AAV-DIO-shPDGFRβ- injected mice. Top images: Scale bar = 100 µm. Bottom images: Scale bar = 10 µm. (**G**) Quantification of the number of BrdU positive cells in DG of the hippocampus in AAV-DIO-shGFP or AAV-DIO-shPDGFRβ-injected mice. n = 4 per group. (**H**) Quantification of the number of BrdU/Sox2/GFAP positive NSCs in SGZ of the hippocampus in AAV-DIO-shGFP or AAV-DIO-shPDGFRβ-injected mice. n = 4 per group. (**I**) The ratio of aNSCs to qNSCs. n = 4 per group. (**J**) Quantification of the number of Sox2/GFAP positive NSCs in SGZ of the hippocampus in AAV-DIO-shGFP or AAV-DIO-shPDGFRβ-injected mice. n = 4 per group. Data are expressed as mean ± SEM. Student's *t* test (A, E, G-J). **P*< 0.05; ***P* < 0.01; ****P* < 0.001. The statistical details can be found in Supplementary Table S4.

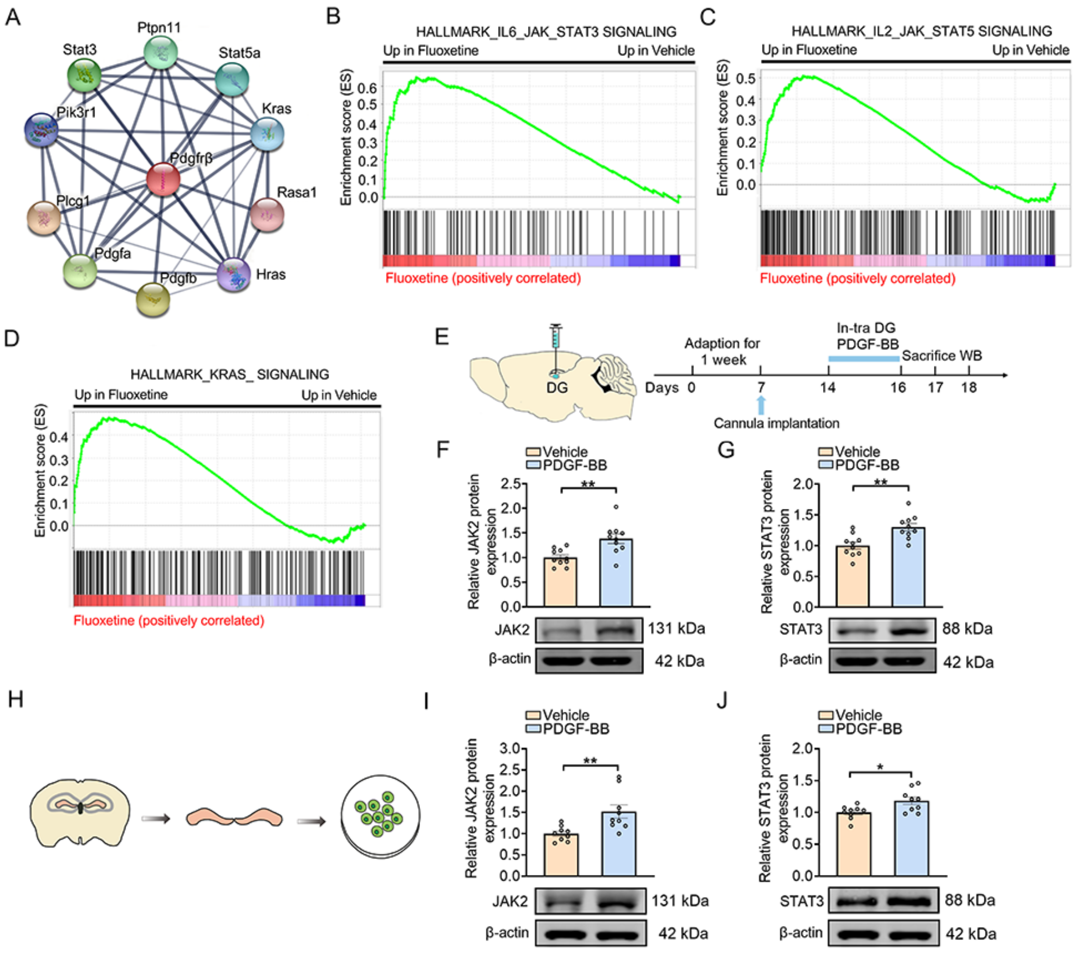

Suppl. Fig. 12 JAK2/STAT3 is involved in the antidepressant action mediated by PDGF-BB/PDGFRβ signaling pathway. (**A**) Functional protein association network plotted by using the STRING database. (**B-D**) GSEA plot of proteins expressed in fluoxetine-treated mice for the list of STAT3, STAT5, KRAS. (**E**) Experimental timeline for behavioral testing after intra-hippocampal infusion of vehicle and PDGF-BB. (**F**) The change of protein levels of JAK2 in the DG after PDGF-BB treatment. n = 10 per group. (**G**) The change of protein levels of STAT3 in the DG after PDGF-BB treatment. n = 10 per group. (**H**) Schematics of the experiments. (**I**) The change of protein levels of JAK2 in the DG after PDGF-BB treatment. n = 9 per group. (**J**) The change of protein levels of STAT3 in the DG after PDGF-BB treatment. n = 9 per group. Data are expressed as mean ± SEM. **P* < 0.05, ***P* < 0.01, ****P* < 0.01. Student's *t* test (F, G, I, J). The statistical details can be found in Supplementary Table S4.

Uncropped scans of key western blots

Figure S11A

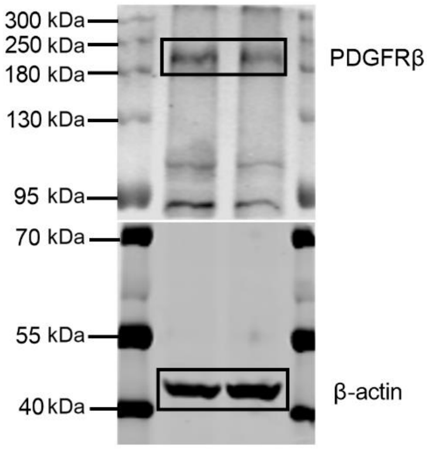

Figure S12F and Figure S12G

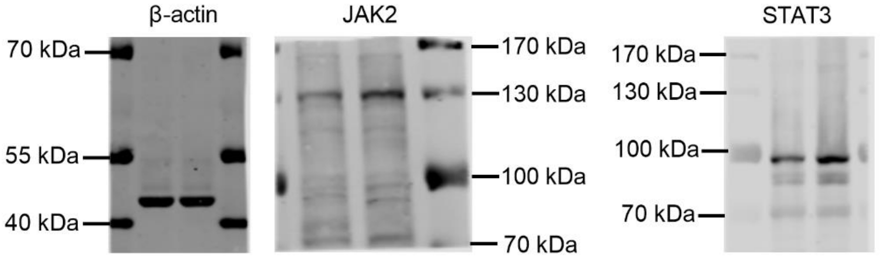

Figure S12I and Figure S12J

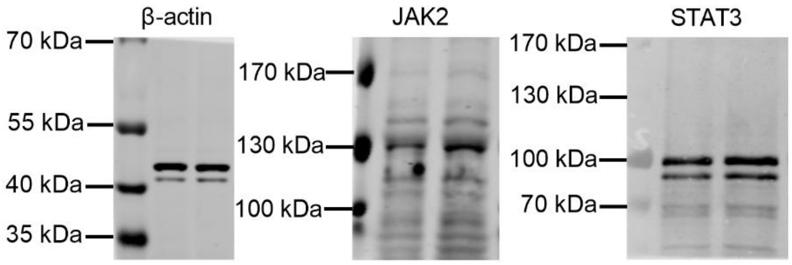

Supplementary Table S1. All primers used.

Names	Sequences (5' to 3')
PDGF-BB-R:	TACGGAGTCTCTGTGCAGCAGGC
PDGF-BB-F:	AGTCGGCATGAATCGCTGCTGGG
GAPDH-R:	ATGGTGAAGGTCGGTGTG
GAPDH-F:	CATTCTCGGCCTTGACTG

Supplementary Table S2. All antibodies and drugs used.

**Antibodies**	**SOURCE**	**IDENTIFIER**
Anti-PDGFRβ	Cell Signaling Technology	Cat# 3169
Anti-PDGFRβ	Abcam	Cat# ab32570
Anti-GFAP	Cell Signaling Technology	Cat# 3670
Anti-Sox2	Abcam	Cat# ab97959
Anti-BrdU	Abcam	Cat# ab6326
Anti-DCX	Cell Signaling Technology	Cat# 4604
Anti-Nestin	Millipore	Cat# MAB353
Anti-CTB	Abnova	Cat# PAB13910
Anti-β-actin	Cell Signaling Technology	Cat# 8480
Anti-Somatostatin-28	Synaptic Systems	Cat# 366004
Anti-Cholecystokinin 8	Abcam	Cat# ab37274
Anti-C-FOS	Abcam	Cat# ab190289
Anti-CCK	Abcam	Cat# ab37274
Anti-CaMKIIα	Cell Signaling Technology	Cat# 50049
Anti-VIP	Santa cruz biotechnology	Cat# sc-25347
Anti-GAD67	Abcam	Cat# ab26116
Anti-JAK2	Proteintech	Cat# 17670-1-AP
Anti-STAT3	Proteintech	Cat# 10253-2-AP
CTB-555	BrainVTA	Cat# 210127
Alex Fluor 488-conjugated donkey anti-mouse IgG	Invitrogen	Cat# A32766
Alex Fluor 488-conjugated donkey anti- rabbit IgG	Invitrogen	Cat# A21206
Alex Fluor 594-conjugated donkey anti-rabbit IgG	Invitrogen	Cat# A21207
Alex Fluor 647-conjugated donkey anti-rat IgG	Jackson ImmunoResearch	Cat# 712-605-153
Alex Fluor 405-conjugated donkey anti-mouse IgG	Abcam	Cat# ab175660
Alex Fluor 647-conjugated donkey anti- rabbit IgG	Invitrogen	Cat# A31573

Supplementary Table S3. All bacterial and virus Strains used.

AAV-EF1a-DIO-hM4D(Gi)-mCherry	Obio Technology Shanghai	HYMBH2481
AAV-EF1a-DIO-hM3D(Gq)-mCherry	Obio Technology Shanghai	HYMBH2482
AAV-hSyn-Cre	Obio Technology Shanghai	CN867
pROV-U6-shRNA-EF1a(S)-EGFP	Obio Technology Shanghai	CN889
qAkd-cmv-bGlobin-Flex-EGFP-MIR30shRNA (pdgfprβ)	Obio Technology Shanghai	Y10057
LV-U6-shPdgfb-Ubi-EGFP	Genechem Shanghai	81302-13
LV-U6-shPdgfb-Ubi-mcherry	Genechem Shanghai	92846-1
LV-Ubi-Pdgfb- Ubi-EGFP	Genechem Shanghai	45561-1
AAV-CMV-Pdgfb-EGFP	Genechem Shanghai	46612-1
AAV-CMV-Pdgfb-mCherry	Genechem Shanghai	61989-2

Supplementary Table S4. Statistical analysis for Figures 1-8 and Figures S1-10.

**Figure and numbers of animals or cells used**	**Statistical analysis**	***Post hoc tests***	**Mean ± SEM.**
**1D:** Behavioral test of SIT<br><br>Control + Vehicle (n = 12)<br>Control + CNO (n = 14)<br>CSDS + Vehicle (n =15)<br>CSDS + CNO (n = 15)	Two-way ANOVA<br><br>Stress: F (1,52) = 4.8290, p = 0.0325<br>Treatment: F (1,52) = 1.2060, p = 0.2772<br>Stress × Treatment: F (1,52) = 7.5570,<br>p = 0.0082	Bonferroni's *post hoc* test<br><br>Control + Vehicle versus CSDS + Vehicle, p = 0.0024<br>Control + CNO versus CSDS + CNO,<br>p > 0.9999<br>Control + Vehicle versus Control + CNO, p = 0.5305<br>CSDS + Vehicle versus CSDS + CNO,<br>p = 0.0133	 <br>Control + Vehicle 1.267 ± 0.179<br>Control + CNO 1.040 ± 0.151<br>CSDS + Vehicle 0.588 ± 0.079<br>CSDS + CNO 1.116 ± 0.137
**1E:** Behavioral test of TST<br>Control + Vehicle (n = 12)<br>Control + CNO (n = 14)<br>CSDS + Vehicle (n = 15)<br>CSDS + CNO (n = 15)	Two-way ANOVA<br><br>Stress: F (1,52) = 1.5620, p = 0.2170<br>Treatment: F (1,52) = 0.4100, p = 0.5248<br>Stress × Treatment: F (1,52) = 6.4500,<br>p = 0.0141	Bonferroni's *post hoc* test<br>Control + Vehicle versus CSDS + Vehicle,<br>p = 0.0226<br>Control + CNO versus CSDS + CNO,<br>p = 0.7117<br>Control + Vehicle versus Control + CNO, p = 0.4016<br>CSDS + Vehicle versus CSDS + CNO,<br>p = 0.0466	**Mean ± s.e.m.**<br><br>Control + Vehicle 127.8 ± 8.0<br>Control + CNO 146.4 ± 8.7<br>CSDS + Vehicle 164.9 ± 8.6<br>CSDS + CNO 133.8 ± 12.2
**1F:** Behavioral test of FST<br><br>Control + Vehicle (n = 12)<br>Control + CNO (n = 14)<br>CSDS + Vehicle (n = 15)<br>CSDS + CNO (n = 15)	Two-way ANOVA<br><br>Stress: F (1,52) = 0.1686, p = 0.6830<br>Treatment: F (1,52) = 15.0300, p = 0.0003<br>Stress × Treatment: F (1,52) = 9.3560,<br>p = 0.0035	Bonferroni's *post hoc* test<br><br>Control + Vehicle versus CSDS + Vehicle,<br>p = 0.0396<br>Control + CNO versus CSDS + CNO,<br>p = 0.1227<br>Control + Vehicle	**Mean ± s.e.m.**<br><br>Control + Vehicle 111.9 ± 7.5<br>Control + CNO 102.6 ± 15.4<br>CSDS + Vehicle 151.5 ± 9.4

		versus Control + CNO, $p > 0.9999$<br>CSDS + Vehicle versus CSDS + CNO, $p < 0.001$	CSDS + CNO 72.3 ± 10.8
**1G:** Behavioral test of OFT<br><br>Control + Vehicle (n = 12)<br>Control + CNO (n = 14)<br>CSDS + Vehicle (n = 15)<br>CSDS + CNO (n = 15)	Two-way ANOVA<br><br>Stress: $F_{(1,52)} = 0.6083$, $p = 0.4390$<br>Treatment: $F_{(1,52)} = 2.9630$, $p = 0.0911$<br>Stress × Treatment: $F_{(1,52)} = 0.01802$, $p = 0.8937$	Bonferroni's *post hoc* test<br><br>Control + Vehicle versus CSDS + Vehicle, $p > 0.9999$<br>Control + CNO versus CSDS + CNO, $p > 0.9999$<br>Control + Vehicle versus Control + CNO, $p = 0.4224$<br>CSDS + Vehicle versus CSDS + CNO, $p = 0.4975$	**Mean ± s.e.m.**<br><br>Control + Vehicle 35.11 ± 3.21<br>Control + CNO 29.67 ± 3.37<br>CSDS + Vehicle 32.43 ± 3.04<br>CSDS + CNO 27.78 ± 1.99
**1K:** Behavioral test of SIT<br>Vehicle (n = 19)<br>CNO (n = 20)	Unpaired *t* test<br><br>$t = 2.0300$, $p = 0.0496$		**Mean ± s.e.m.**<br><br>Vehicle 1.408 ± 0.228<br>CNO 0.876 ± 0.136
**1L:** Behavioral test of TST<br><br>Vehicle (n = 19)<br>CNO (n = 20)	Unpaired *t* test<br><br>$t = 2.7510$, $p = 0.0091$		**Mean ± s.e.m.**<br><br>Vehicle 130.1 ± 5.6<br>CNO 150.9 ± 5.1
**1M:** Behavioral test of FST<br><br>Vehicle (n = 19)<br>CNO (n = 20)	Unpaired *t* test<br><br>$t= 3.7140$, $p = 0.0007$		**Mean ± s.e.m.**<br><br>Vehicle 97.8 ± 8.1<br>CNO 139.4 ± 7.8
**1N:** Behavioral test of OFT<br><br>Vehicle (n = 19)<br>CNO (n = 20)	Unpaired *t* test<br><br>$t = 0.6909$, $p = 0.4939$		**Mean ± s.e.m.**<br><br>Vehicle 22.33 ± 1.87<br>CNO 20.68 ± 1.50
**2C:** DREADD inhibition of MS-DG GABAergic projections led to an	Unpaired *t* test		**Mean ± s.e.m.**

increment of c-Fos expression in DG.<br><br>Vehicle (n = 3)<br>CNO (n = 3)	 <br><br>t = 3.0440, p = 0.0383		 <br><br>Vehicle 1150.0 ± 278.7<br>CNO 2325.0 ± 266.9
**2D:** DREADD inhibition of MS-DG GABAergic projections led to an increment of c-Fos expression in SOM positive neurons.<br><br>Vehicle (n = 3)<br>CNO (n = 3)	Unpaired *t* test<br><br>t = 2.8910, p = 0.0445		**Mean ± s.e.m.**<br><br>Vehicle 153.3 ± 21.9<br>CNO 527.7 ± 127.6
**2F:** DREADD inhibition of MS-DG GABAergic projections led to an increment of PDGF-BB expression in DG.<br><br>Vehicle (n = 3)<br>CNO (n = 3)	Unpaired *t* test<br><br>t = 4.8190, p = 0.0085		**Mean ± s.e.m.**<br><br>Vehicle 1450.0 ± 531.5<br>CNO 4040.0 ± 79.4
**2G:** DREADD inhibition of MS-DG GABAergic projections led to an increment of PDGF-BB expression in SOM positive neurons.<br><br>Vehicle (n = 3)<br>CNO (n = 3)	Unpaired *t* test<br><br>t = 10.8000, p = 0.0004		**Mean ± s.e.m.**<br><br>Vehicle 470.0 ± 189.3<br>CNO 2523.0 ± 18.6
**3C:** Behavioral test of SIT<br><br>Control + AAV-GFP (n = 15)<br>Control + AAV-PDGF-BB (n = 13)	Two-way ANOVA<br><br>Stress: F (1,54) = 1.6910, p = 0.1990<br>Virus: F (1,54) = 13.5900, p = 0.0005	Bonferroni's *post hoc* test<br><br>Control + AAV-GFP versus CSDS + AAV-GFP, p = 0.0021	**Mean ± s.e.m.**<br><br>Control + AAV-GFP 1.367 ± 0.162<br>Control + AAV-PDGF-BB 1.382 ± 0.119

CSDS + AAV-GFP (n = 14)<br>CSDS + AAV-PDGF-BB (n = 16)	Stress × Virus: $F(1,54) = 12.8700$, $p = 0.0007$	Control + AAV-PDGF-BB versus CSDS + AAV-PDGF-BB,<br>$p = 0.2249$<br>Control + AAV-GFP versus Control + AAV-PDGF-BB,<br>$p > 0.9999$<br>CSDS + AAV-GFP versus CSDS + AAV-PDGF-BB, $p < 0.001$	CSDS + AAV-GFP 0.626 ± 0.146<br>CSDS + AAV-PDGF-BB 1.729 ± 0.162
**3D:** Behavioral test of TST<br><br>Control + AAV-GFP (n = 15)<br>Control + AAV-PDGF-BB (n = 13)<br>CSDS + AAV-GFP (n = 14)<br>CSDS + AAV-PDGF-BB (n = 16)	Two-way ANOVA<br><br>Stress: $F(1,54) = 5.0870$, $p = 0.0282$,<br>Virus: $F(1,54) = 40.8600$, $p < 0.001$<br>Stress × Virus: $F(1,54) = 7.8840$, $p = 0.0069$	Bonferroni's *post hoc* test<br><br>Control + AAV-GFP versus CSDS + AAV-GFP, $p = 0.0014$<br>Control + AAV-PDGF-BB versus CSDS + AAV-PDGF-BB,<br>$p > 0.9999$<br>Control + AAV-GFP versus Control + AAV-PDGF-BB,<br>$p = 0.0317$<br>CSDS + AAV-GFP versus CSDS + AAV-PDGF-BB,<br>$p < 0.001$	**Mean ± s.e.m.**<br><br>Control + AAV-GFP 131.6 ± 4.3<br>Control + AAV-PDGF-BB 104.9 ± 5.0<br>CSDS + AAV-GFP 169.3 ± 6.4<br>CSDS + AAV-PDGF-BB 100.8 ± 10.8
**3E:** Behavioral test of FST<br><br>Control + AAV-GFP (n = 15)<br>Control + AAV-PDGF-BB (n = 13)<br>CSDS + AAV-GFP (n = 14)<br>CSDS + AAV-PDGF-BB (n = 16)	Two-way ANOVA<br><br>Stress: $F(1,54) = 0.1426$, $p = 0.7072$<br>Virus: $F(1,54) = 7.4140$, $p = 0.0087$<br>Stress × Virus: $F(1,54) = 23.5700$, $p < 0.001$	Bonferroni's *post hoc* test<br><br>Control + AAV-GFP versus CSDS + AAV-GFP, $p = 0.0050$<br>Control + AAV-PDGF-BB versus CSDS + AAV-PDGF-BB,<br>$p = 0.0010$<br>Control + AAV-GFP versus Control + AAV-PDGF-BB,<br>$p = 0.2883$	**Mean ± s.e.m.**<br><br>Control + AAV-GFP 74.9 ± 10.6<br>Control + AAV-PDGF-BB 96.5 ± 10.4<br>CSDS + AAV-GFP 120.1 ± 11.6<br>CSDS + AAV-PDGF-BB 43.6 ± 7.7

		CSDS + AAV-GFP versus CSDS + AAV-PDGF-BB, p < 0.001	
**3F:** Behavioral test of OFT<br><br>Control + AAV-GFP (n = 15)<br>Control + AAV-PDGF-BB (n = 13)<br>CSDS + AAV-GFP (n = 14)<br>CSDS + AAV-PDGF-BB (n = 16)	Two-way ANOVA<br><br>Stress: F (1,54) = 0.0012, p = 0.9728<br>Virus: F (1,54) =1.4050, p = 0.2411<br>Stress × Virus: F (1,54) = 0.5903,<br>p = 0.4456	Bonferroni's *post hoc* test<br><br>Control + AAV-GFP versus CSDS + AAV-GFP, p > 0.9999<br>Control + AAV-PDGF-BB versus CSDS + AAV-PDGF-BB,<br>p > 0.9999<br>Control + AAV-GFP versus Control + AAV-PDGF-BB,<br>p > 0.9999<br>CSDS + AAV-GFP versus CSDS + AAV-PDGF-BB, p = 0.3309	**Mean ± s.e.m.**<br><br>Control + AAV-GFP 43.90 ± 3.28<br>Control + AAV-PDGF-BB 45.57 ± 5.42<br>CSDS + AAV-GFP 40.97 ± 4.00<br>CSDS + AAV-PDGF-BB 48.77 ± 3.33
**3H:** Behavioral test of SIT<br><br>Control + Vehicle (n = 12)<br>Control + PDGF-BB (n = 12)<br>CSDS + Vehicle (n = 12)<br>CSDS + PDGF-BB (n = 15)	Two-way ANOVA<br><br>Stress: F (1,47) = 25.8700, p < 0.001<br>Treatment: F (1,47) = 3.1380, p = 0.0830<br>Stress × Treatment: F (1,47) = 8.4590,<br>p = 0.0055	Bonferroni's *post hoc* test<br><br>Control + Vehicle versus CSDS + Vehicle, p < 0.001<br>Control + PDGF-BB versus CSDS + PDGF-BB, p =0.2408<br>Control + Vehicle versus Control + PDGF-BB, p = 0.8744<br>CSDS + Vehicle versus CSDS + PDGF-BB,<br>p = 0.0028	**Mean ± s.e.m.**<br><br>Control + Vehicle 1.263 ± 0.089<br>Control + PDGF-BB 1.125 ± 0.111<br>CSDS + Vehicle 0.297 ± 0.064<br>CSDS + PDGF-BB 0.862 ± 0.161
**3I:** Behavioral test of TST<br><br>Control + Vehicle (n = 12)<br>Control + PDGF-BB (n = 12)<br>CSDS + Vehicle (n = 12)	Two-way ANOVA<br><br>Stress: F (1,47) = 0.3129, p = 0.5785<br>Treatment: F (1,47) = 5.6880, p = 0.0212	Bonferroni's *post hoc* test<br><br>Control + Vehicle versus CSDS + Vehicle, p = 0.0218<br>Control + PDGF-BB versus CSDS +	**Mean ± s.e.m.**<br><br>Control + Vehicle 124.4 ± 5.5<br>Control + PDGF-BB 132.3 ± 8.1

CSDS + PDGF-BB (n = 15)	Stress × Virus: F (1,47) = 10.8100,<br>p = 0.0019	PDGF-BB, p = 0.1068<br>Control + Vehicle versus Control + PDGF-BB, p > 0.9999<br>CSDS + Vehicle versus CSDS + PDGF-BB, p = 0.0003	CSDS + Vehicle 158.2 ± 9.6<br>CSDS + PDGF-BB 108.4 ± 10.0
**3J:** Behavioral test of FST<br><br>Control + Vehicle (n = 12)<br>Control + PDGF-BB (n = 12)<br>CSDS + Vehicle (n = 12)<br>CSDS + PDGF-BB<br>(n = 15)	Two-way ANOVA<br><br>Stress: F (1,47) = 0.1019, p = 0.7510<br>Treatment: F (1,47) = 12.2400, p = 0.0010<br>Stress × Virus: F (1,47) = 12.6700,<br>p = 0.0009;	Bonferroni's *post hoc* test<br><br>Control + Vehicle versus CSDS + Vehicle, p = 0.0206<br>Control + PDGF-BB versus CSDS + PDGF-BB, p = 0.0456<br>Control + Vehicle versus Control + PDGF-BB, p > 0.9999<br>CSDS + Vehicle versus CSDS + PDGF-BB, p < 0.001	**Mean ± s.e.m.**<br><br>Control + Vehicle 85.7 ± 10.8<br>Control + PDGF-BB 86.3 ± 8.0<br>CSDS + Vehicle 122.8 ± 9.0<br>CSDS + PDGF-BB 55.2 ± 9.8
**3K:** Behavioral test of OFT<br><br>Control + Vehicle (n = 12)<br>Control + PDGF-BB (n = 12)<br>CSDS + Vehicle (n = 12)<br>CSDS + PDGF-BB (n = 15)	Two-way ANOVA<br><br>Stress: F (1,47) = 0.3096, p = 0.5806<br>Treatment: F (1,47) = 0.0005, p = 0.9828<br>Stress × Virus: F (1,47) = 4.5460, p = 0.0383	Bonferroni's *post hoc* test<br><br>Control + Vehicle versus CSDS + Vehicle, p = 0.1404<br>Control + PDGF-BB versus CSDS + PDGF-BB, p = 0.5163<br>Control + Vehicle versus Control + PDGF-BB, p = 0.2888<br>CSDS + Vehicle versus CSDS + PDGF-BB,<br>p = 0.2639	**Mean ± s.e.m.**<br><br>Control + Vehicle 54.43 ± 5.93<br>Control + PDGF-BB 46.28 ± 3.42<br>CSDS + Vehicle 44.25 ± 2.00<br>CSDS + PDGF-BB 52.24 ± 2.92
**4C:** Behavioral test of SIT<br><br>LV-shGFP (n = 15)<br>LV-shPDGF-BB (n = 15)	Unpaired *t* test<br><br>t = 2.8890, p = 0.0074		**Mean ± s.e.m.**<br><br>LV-shGFP 1.285 ± 0.167<br>LV-shPDGF-BB 0.674 ± 0.130

**4D:** Behavioral test of TST<br><br>LV-shGFP (n = 15)<br>LV-shPDGF-BB (n = 15)	Unpaired *t* test<br><br>t = 2.7370, p = 0.0106		**Mean ± s.e.m.**<br><br>LV-shGFP 132.2 ± 9.2<br>LV-shPDGF-BB 168.5 ± 9.5
**4E:** Behavioral test of FST<br><br>LV-shGFP (n = 15)<br>LV-shPDGF-BB (n = 15)	Unpaired *t* test<br><br>t = 3.0710, p = 0.0047		**Mean ± s.e.m.**<br><br>LV-shGFP<br>112.0 ± 9.2<br>LV-shPDGF-BB<br>147.9 ± 7.3
**4F:** Behavioral test of OFT<br><br>LV-shGFP (n = 15)<br>LV-shPDGF-BB (n = 15)	Unpaired *t* test<br><br>t = 0.1401, p = 0.8896		**Mean ± s.e.m.**<br><br>LV-shGFP 21.24 ± 2.05<br>LV-shPDGF-BB<br>21.62 ± 1.75
**4H:** Behavioral test of SIT<br><br>CSDS + Vehicle + Anti-IgG (n = 14)<br>CSDS + Vehicle + Anti-PDGF-BB (n = 15)<br>CSDS + CNO + Anti-IgG (n = 16)<br>CSDS + CNO + Anti-PDGF-BB (n = 16)	Two-way ANOVA<br><br>Treatment: F (1,57) = 0.9231, p = 0.3407<br>Treatment: F (1,57) = 15.6200, p = 0.0002<br>Treatment × Treatment: F (1,57) = 6.3810, p = 0.0143	Bonferroni's *post hoc* test<br><br>CSDS + Vehicle + Anti-IgG versus CSDS + CNO + Anti-IgG,<br>p < 0.001<br>CSDS + Vehicle + Anti-PDGF-BB versus<br>CSDS + CNO + Anti-PDGF-BB, p = 0.6263<br>CSDS + Vehicle + Anti-IgG versus CSDS + Vehicle + Anti-PDGF-BB,<br>p = 0.5692<br>CSDS + CNO + Anti-IgG versus CSDS + CNO + Anti-PDGF-BB, p = 0.0284	**Mean ± s.e.m.**<br><br>CSDS + Vehicle + Anti-IgG 0.292 ± 0.080<br>CSDS + Vehicle + Anti-PDGF-BB<br>0.418 ± 0.091<br>CSDS + CNO + Anti-IgG 0.814 ± 0.076<br>CSDS + CNO + Anti-PDGF-BB<br>0.533 ± 0.076
**4I:** Behavioral test of TST	Two-way ANOVA	Bonferroni's *post hoc* test	**Mean ± s.e.m.**

CSDS + Vehicle + Anti-IgG (n = 14)<br>CSDS + Vehicle + Anti-PDGF-BB (n = 15)<br>CSDS + CNO + Anti-IgG (n = 17)<br>CSDS + CNO + Anti-PDGF-BB (n = 16)	Treatment: $F (1,58) = 8.8430$, $p = 0.0043$<br>Treatment: $F (1,58) = 8.800$, $p = 0.0044$<br>Treatment × Treatment: $F (1,58) = 4.3430$, $p = 0.0416$	CSDS + Vehicle + Anti-IgG versus CSDS + CNO + Anti-IgG,<br>$p = 0.0015$<br>CSDS + Vehicle + Anti-PDGF-BB versus CSDS + CNO + Anti-PDGF-BB, $p > 0.9999$<br>CSDS + Vehicle + Anti-IgG versus CSDS + Vehicle + Anti-PDGF-BB,<br>$p > 0.9999$<br>CSDS + CNO + Anti-IgG versus CSDS + CNO + Anti-PDGF-BB, $p = 0.0010$	CSDS + Vehicle + Anti-IgG 165.5 ± 7.2<br>CSDS + Vehicle + Anti-PDGF-BB 173.3 ± 10.6<br>CSDS + CNO + Anti-IgG 121.4 ± 9.1<br>CSDS + CNO + Anti-PDGF-BB 165.6 ± 7.2
**4J:** Behavioral test of FST<br><br>CSDS + Vehicle + Anti-IgG (n = 14)<br>CSDS + Vehicle + Anti-PDGF-BB (n = 15)<br>CSDS + CNO + Anti-IgG (n = 17)<br>CSDS + CNO + Anti-PDGF-BB (n = 16)	Two-way ANOVA<br><br>Treatment: $F (1,58) = 4.2380$, $p = 0.0440$<br>Treatment: $F (1,58) = 1.5830$, $p = 0.2134$<br>Treatment × Treatment: $F (1,58) = 4.0880$, $p = 0.0478$	Bonferroni's *post hoc* test<br><br>CSDS + Vehicle + Anti-IgG versus CSDS + CNO + Anti-IgG,<br>$p = 0.0484$<br>CSDS + Vehicle + Anti-PDGF-BB versus CSDS + CNO + Anti-PDGF-BB, $p > 0.9999$<br>CSDS + Vehicle + Anti-IgG versus CSDS + Vehicle + Anti-PDGF-BB,<br>$p > 0.9999$<br>CSDS + CNO + Anti-IgG versus CSDS + CNO + Anti-PDGF-BB, $p = 0.0083$	**Mean ± s.e.m.**<br><br>CSDS + Vehicle + Anti-IgG 134.0 ± 8.5<br>CSDS + Vehicle + Anti-PDGF-BB 134.3 ± 10.9<br>CSDS + CNO + Anti-IgG 104.3 ± 8.5<br>CSDS + CNO + Anti-PDGF-BB 141.3 ± 8.1
**4K:** Behavioral test of OFT<br><br>CSDS + Vehicle + Anti-IgG (n = 14)<br>CSDS + Vehicle + Anti-PDGF-BB (n = 15)	Two-way ANOVA<br><br>Treatment: $F (1,59) = 0.0007$, $p = 0.9785$<br>Treatment: $F (1,59) = 0.02334$, $p = 0.8791$	Bonferroni's *post hoc* test<br><br>CSDS + Vehicle + Anti-IgG versus CSDS + CNO + Anti-IgG,<br>$p > 0.9999$	**Mean ± s.e.m.**<br><br>CSDS + Vehicle + Anti-IgG 30.65 ± 3.05

CSDS + CNO + Anti-IgG (n = 17)<br>CSDS + CNO + Anti-PDGF-BB (n = 17)	Treatment × Treatment: F (1,59) = 0.0274, p = 0.8691	CSDS + Vehicle + Anti-PDGF-BB versus CSDS + CNO + Anti-PDGF-BB, p > 0.9999<br>CSDS + Vehicle + Anti-IgG versus CSDS + Vehicle + Anti-PDGF-BB,<br>p > 0.9999<br>CSDS + CNO + Anti-IgG versus CSDS + CNO + Anti-PDGF-BB, p > 0.9999	CSDS + Vehicle + Anti-PDGF-BB 31.20 ± 2.43<br>CSDS + CNO + Anti-IgG 30.69 ± 2.66<br>CSDS + CNO + Anti-PDGF-BB 30.30 ± 3.08
**5C:** Quantification of the number of BrdU positive cells<br><br>Control + LV-GFP (n =4)<br>Control + LV-PDGF-BB (n = 4)<br>CSDS + LV-GFP (n = 4)<br>CSDS + LV-PDGF-BB (n = 4)	Two-way ANOVA<br><br>Stress: F (1,12) = 7.9930, p = 0.0153<br>Virus: F (1,12) = 61.5700, p < 0.001<br>Stress × Virus: F (1,12) = 10.6900, p = 0.0067	Bonferroni's *post hoc* test<br><br>Control + LV-GFP versus CSDS + LV-GFP, p = 0.0020<br>Control + LV-PDGF-BB versus CSDS + LV-PDGF-BB, p > 0.9999<br>Control + LV-GFP versus Control + LV-PDGF-BB,<br>p = 0.0143<br>CSDS + LV-GFP versus CSDS + LV-PDGF-BB,<br>p < 0.001	**Mean ± s.e.m.**<br><br>Control + LV-GFP 2238.0 ± 164.8<br>Control + LV-PDGF-BB 2833.0 ± 152.1<br>CSDS + LV-GFP 1445.0 ± 60.1<br>CSDS + LV-PDGF-BB 2890.0 ± 116.9
**5D:** Quantification of the number of BrdU/DCX positive cells<br><br>Control + LV-GFP (n =4)<br>Control + LV-PDGF-BB (n = 4)<br>CSDS + LV-GFP (n = 4)<br>CSDS + LV-PDGF-BB (n = 4)	Two-way ANOVA<br><br>Stress: F (1,12) = 9.4360, p = 0.0097<br>Virus: F (1,12) = 25.2700, p = 0.0003<br>Stress × Virus: F (1,12) = 5.5250,<br>p = 0.0367	Bonferroni's *post hoc* test<br><br>Control + LV-GFP versus CSDS + LV-GFP, p = 0.0048<br>Control + LV-PDGF-BB versus CSDS + LV-PDGF-BB, p > 0.9999<br>Control + LV-GFP versus Control + LV-PDGF-BB,<br>p = 0.1656	**Mean ± s.e.m.**<br><br>Control + LV-GFP 1380.0 ± 117.3<br>Control + LV-PDGF-BB 1668.0 ± 95.5<br>CSDS + LV-GFP 797.5 ± 94.2<br>CSDS + LV-PDGF-BB 1590.0 ± 120.1

		CSDS + LV-GFP versus CSDS + LV-PDGF-BB, $p = 0.0004$	
**5F:** Quantification of the number of BrdU/Sox2/GFAP positive NSCs<br><br>Control + LV-GFP (n =4)<br>Control + LV-PDGF-BB (n = 4)<br>CSDS + LV-GFP (n = 4)<br>CSDS + LV-PDGF-BB (n = 4)	Two-way ANOVA<br><br>Stress: $F(1,12) = 15.9500$, $p = 0.0018$<br>Virus: $F(1,12) = 22.7200$, $p = 0.0005$<br>Stress × Virus: $F(1,12) = 3.2040$,<br>$p = 0.0987$	Bonferroni's *post hoc* test<br><br>Control + LV-GFP versus CSDS + LV-GFP, $p = 0.0030$<br>Control + LV-PDGF-BB versus CSDS + LV-PDGF-BB, $p = 0.2904$<br>Control + LV-GFP versus Control + LV-PDGF-BB,<br>$p = 0.1141$<br>CSDS + LV-GFP versus CSDS + LV-PDGF-BB, $p = 0.0011$	**Mean ± s.e.m.**<br><br>Control + LV-GFP 557.5 ± 86.4<br>Control + LV-PDGF-BB 737.5 ± 19.3<br>CSDS + LV-GFP 207.8 ± 32.0<br>CSDS + LV-PDGF-BB 604.3 ± 76.0
**5G:** Quantification of the number of Sox2/GFAP positive NSCs<br><br>Control + LV-GFP (n =4)<br>Control + LV-PDGF-BB (n = 4)<br>CSDS + LV-GFP (n = 4)<br>CSDS + LV-PDGF-BB (n = 4)	Two-way ANOVA<br><br>Stress: $F(1,12) = 0.8694$, $p = 0.3695$<br>Virus: $F(1,12) = 5.8640$, $p = 0.0322$<br>Stress × Virus: $F(1,12) = 0.08552$, $p = 0.7749$	Bonferroni's *post hoc* test<br><br>Control + LV-GFP versus CSDS + LV-GFP, $p > 0.9999$<br>Control + LV-PDGF-BB versus CSDS + LV-PDGF-BB, $p = 0.8068$<br>Control + LV-GFP versus Control + LV-PDGF-BB,<br>$p = 0.3161$<br>CSDS + LV-GFP versus CSDS + LV-PDGF-BB, $p = 0.1581$	**Mean ± s.e.m.**<br><br>Control + LV-GFP 7825.0 ± 195.9<br>Control + LV-PDGF-BB 8250.0 ± 108.6<br>CSDS + LV-GFP 7953.0 ± 201.9<br>CSDS + LV-PDGF-BB 8495.0 ± 261.6
**5H:** The ratio of aNSCs to qNSCs<br><br>Control + LV-GFP (n =4)<br>Control + LV-PDGF-BB (n = 4)	Two-way ANOVA<br><br>Stress: $F(1,12) = 23.1900$, $p = 0.0004$	Bonferroni's *post hoc* test<br><br>Control + LV-GFP versus CSDS + LV-GFP, $p = 0.0009$	**Mean ± s.e.m.**<br><br>Control + LV-GFP 1.00 ± 0.15

CSDS + LV-GFP (n = 4)<br>CSDS + LV-PDGF-BB (n = 4)	Virus: F (1,12) = 22.7700, p = 0.0005<br>Stress × Virus: F (1,12) = 3.8660, p = 0.0728	Control + LV-PDGF-BB versus CSDS + LV-PDGF-BB, p = 0.1337<br>Control + LV-GFP versus Control + LV-PDGF-BB,<br>p = 0.1413<br>CSDS + LV-GFP versus CSDS + LV-PDGF-BB,<br>p = 0.0009	Control + LV-PDGF-BB 1.26 ± 0.02<br>CSDS + LV-GFP 0.37 ± 0.06<br>CSDS + LV-PDGF-BB 0.99 ± 0.10
**5K:** Quantification of branch number<br><br>Control + AAV-mCherry (n =26)<br>Control + AAV-PDGF-BB (n = 25)<br>CSDS + AAV-mCherry (n = 27)<br>CSDS + AAV-PDGF-BB (n = 25)	Two-way ANOVA<br><br>Stress: F (1,99) = 18.0900, p < 0.001<br>Virus: F (1,99) = 18.9300, p < 0.001<br>Stress × Virus: F (1,99) = 34.2900, p < 0.001	Bonferroni's *post hoc* test<br><br>Control + AAV-mCherry versus CSDS + AAV-mCherry, p < 0.001<br>Control + AAV-PDGF-BB versus CSDS + AAV-PDGF-BB,<br>p = 0.5330<br>Control + AAV-mCherry versus Control + AAV-PDGF-BB,<br>p = 0.5838<br>CSDS + AAV-mCherry versus CSDS + AAV-PDGF-BB, p < 0.001	**Mean ± s.e.m.**<br><br>Control + AAV-mCherry 5.7 ± 0.3<br>Control + AAV-PDGF-BB 5.3 ± 0.4<br>CSDS + AAV-mCherry 2.7 ± 0.2<br>CSDS + AAV-PDGF-BB 5.8 ± 0.3
**5L:** Quantification of the dendritic length<br><br>Control + AAV-mCherry (n =26)<br>Control + AAV-PDGF-BB (n = 25)<br>CSDS + AAV-mCherry (n = 27)<br>CSDS + AAV-PDGF-BB (n = 25)	Two-way ANOVA<br><br>Stress: F (1,99) = 10.4200, p = 0.0017<br>Virus: F (1,99) = 10.2100, p = 0.0019<br>Stress × Virus: F (1,99) = 32.1500,<br>p < 0.001	Bonferroni's *post hoc* test<br><br>Control + AAV-mCherry versus CSDS + AAV-mCherry, p < 0.001<br>Control + AAV-PDGF-BB versus CSDS + AAV-PDGF-BB,<br>p = 0.1837<br>Control + AAV-mCherry versus Control + AAV-PDGF-BB, p = 0.1694<br>CSDS + AAV-mCherry versus CSDS + AAV-PDGF-BB, p < 0.001	**Mean ± s.e.m.**<br><br>Control + AAV-mCherry 703.6 ± 32.4<br>Control + AAV-PDGF-BB 618.2 ± 39.1<br>CSDS + AAV-mCherry 396.3 ± 28.8<br>CSDS + AAV-PDGF-BB 702.5 ± 37.8
**6C:** Quantification of the	Unpaired *t* test		**Mean ± s.e.m.**

number of BrdU positive cells<br><br>LV-shGFP (n = 4)<br>LV-shPDGF-BB (n = 4)	 <br><br>t = 2.5100, p = 0.0459		 <br><br>LV-shGFP<br>2102.0 ± 197.0<br>LV-shPDGF-BB<br>1364.0 ± 218.3
**6D:** Quantification of the number of BrdU/DCX<br><br>LV-shGFP (n = 4)<br>LV-shPDGF-BB (n = 4)	Unpaired *t* test<br><br><br>t = 2.7740, p = 0.0322		**Mean ± s.e.m.**<br><br><br>LV-shGFP<br>1036.0 ± 127.8<br>LV-shPDGF-BB<br>580.0 ± 103.2
**6F:** Quantification of the number of BrdU/Sox2/GFAP positive NSCs<br><br>LV-shGFP (n = 4)<br>LV-shPDGF-BB (n = 4)	Unpaired *t* test<br><br><br><br><br>t = 3.5650, p = 0.0119		**Mean ± s.e.m.**<br><br><br><br><br>LV-shGFP<br>362.5 ± 23.9<br>LV-shPDGF-BB<br>173.0 ± 47.5
**6G:** Quantification of the number of Sox2/GFAP positive NSCs<br><br>LV-shGFP (n = 4)<br>LV-shPDGF-BB (n = 4)	Unpaired *t* test<br><br><br><br>t = 0.2787, p = 0.7898		**Mean ± s.e.m.**<br><br><br><br>LV-shGFP<br>6515.0 ± 120.7<br>LV-shPDGF-BB<br>6384.0 ± 454.2
**6H:** The ratio of aNSCs to qNSCs<br><br>LV-shGFP (n = 4)<br>LV-shPDGF-BB (n = 4)	Unpaired *t* test<br><br><br>t = 3.5560, p = 0.0120		**Mean ± s.e.m.**<br><br><br>LV-shGFP<br>1.00 ± 0.08<br>LV-shPDGF-BB<br>0.49 ± 0.12
**6K:** Quantification of branch number<br><br>LV-shmCherry (n = 25 neurons)	Unpaired *t* test<br><br><br>t = 6.0250, p < 0.001		**Mean ± s.e.m.**<br><br><br>LV-shCherry<br>6.4 ± 0.4<br>LV-shPDGF-BB

LV-shPDGF-BB (n = 25 neurons)			3.3 ± 0.3
**6L:** Quantification of the dendritic length<br><br>LV-shmCherry (n = 25 neurons)<br>LV-shPDGF-BB (n = 25 neurons)	Unpaired *t* test<br><br>t = 6.0040, p < 0.001		**Mean ± s.e.m.**<br><br>LV-shCherry<br>813.5 ± 40.6<br>LV-shPDGF-BB<br>477.9 ± 38.5
**7C:** Behavioral tests of SIT<br><br>AAV-DIO-shGFP (n = 15)<br>AAV-DIO-shPDGFRβ (n =12)	Unpaired *t* test<br><br>t = 2.8320, p = 0.0090		**Mean ± s.e.m.**<br><br>AAV-DIO-shGFP<br>1.247 ± 0.139 AAV-DIO-shPDGFβ<br>0.517 ± 0.230
**7D:** Behavioral tests of TST<br><br>AAV-DIO-shGFP (n = 15)<br>AAV-DIO-shPDGFRβ (n=12)	Unpaired t test<br><br>t = 2.0690, p = 0.0490		**Mean ± s.e.m.**<br><br>AAV-DIO-shGFP<br>137.9 ± 11.5<br>AAV-DIO-shPDGFβ 170.2 ± 9.9
**7E:** Behavioral tests of FST<br><br>AAV-DIO-shGFP (n = 15)<br>AAV-DIO-shPDGFRβ (n =12)	Unpaired t test<br><br>t = 2.2650, p = 0.0324		**Mean ± s.e.m.**<br><br>AAV-DIO-shGFP<br>96.0 ± 11.3<br>AAV-DIO-shPDGFβ 132.2 ± 10.9
**7F:** Behavioral tests of OFT<br><br>AAV-DIO-shGFP (n = 15)<br>AAV-DIO-shPDGFRβ (n =12)	Unpaired t test<br><br>t = 0.1951, p = 0.8469		**Mean ± s.e.m.**<br><br>AAV-DIO-GFP<br>31.28 ± 2.98<br>AAV-DIO-shPDGFβ 32.11 ± 2.94
**7H:** Behavioral tests of FST	Two-way ANOVA<br><br>Treatment: F (1,47) =	Bonferroni's *post hoc* test	**Mean ± s.e.m.**

AAV-DIO-shGFP + Vehicle (n = 13)<br>AAV-DIO-shGFP + PDGF-BB (n = 14)<br>AAV-DIO-shPDGFRβ + Vehicle (n = 12)<br>AAV-DIO-shPDGFRβ + PDGF-BB (n = 12)	0.4228, $p = 0.5187$<br>Virus: $F (1,47) = 14.8400$, $p = 0.0004$<br>Treatment × Virus: $F (1,47) = 7.9790$,<br>$p = 0.0069$	AAV-DIO-shGFP + Vehicle versus AAV-DIO-shPDGFRβ + Vehicle, $p = 0.9496$<br>AAV-DIO-shGFP + PDGF-BB versus AAV-DIO-shPDGFRβ + PDGF-BB,<br>$p < 0.001$<br>AAV-DIO-shGFP + Vehicle versus AAV-DIO-shGFP + PDGF-BB, $p = 0.0295$<br>AAV-DIO-shPDGFRβ + Vehicle versus AAV-DIO-shPDGFRβ + PDGF-BB,<br>$p = 0.2833$	AAV-DIO-shGFP + Vehicle 120.7 ± 4.8<br>AAV-DIO-shGFP + PDGF-BB 94.9 ± 8.7<br>AAV-DIO-shPDGFRβ + Vehicle 128.3 ± 5.9<br>AAV-DIO-shPDGFRβ + PDGF-BB 144.5 ± 9.2
**7I:** Behavioral tests of OFT<br><br>AAV-DIO-shGFP + Vehicle (n = 13)<br>AAV-DIO-shGFP + PDGF-BB (n = 14)<br>AAV-DIO-shPDGFRβ + Vehicle (n = 12)<br>AAV-DIO-shPDGFRβ + PDGF-BB (n = 12)	Two-way ANOVA<br><br>Treatment: $F (1,47) = 0.4061$, $p = 0.5271$<br>Virus: $F (1,47) = 2.8470$, $p = 0.0982$<br>Treatment × Virus: $F (1,47) = 0.04302$, $p = 0.8366$	Bonferroni's *post hoc* test<br><br>AAV-DIO-shGFP + Vehicle versus AAV-DIO-shPDGFRβ + Vehicle, $p = 0.3810$<br>AAV-DIO-shGFP + PDGF-BB versus AAV-DIO-shPDGFRβ + PDGF-BB,<br>$p = 0.5929$<br>AAV-DIO-shGFP + Vehicle versus AAV-DIO-shGFP + PDGF-BB, $p > 0.9999$<br>AAV-DIO-shPDGFRβ + Vehicle versus AAV-DIO-shPDGFRβ + PDGF-BB,<br>$p > 0.9999$	**Mean ± s.e.m.**<br><br>AAV-DIO-shGFP + Vehicle 42.34 ± 2.50<br>AAV-DIO-shGFP + PDGF-BB 40.02 ± 3.30<br>AAV-DIO-shPDGFRβ + Vehicle 37.14 ± 2.53<br>AAV-DIO-shPDGFRβ + PDGF-BB 35.96 ± 2.31
**7K:** Quantification of the number of BrdU positive cells	Two-way ANOVA	Bonferroni's *post hoc* test	**Mean ± s.e.m.**

AAV-DIO-shGFP + Vehicle (n = 3)<br>AAV-DIO-shGFP + PDGF-BB (n = 4)<br>AAV-DIO-shPDGFRβ + Vehicle (n = 3)<br>AAV-DIO-shPDGFRβ + PDGF-BB (n =4)	Treatment: $F (1,10) = 0.3994$, $p = 0.5416$<br>Virus: $F (1,10) = 5.8770$, $p = 0.0358$<br>Treatment × Virus: $F (1,10) = 15.8800$, $p = 0.0026$	AAV-DIO-shGFP + Vehicle versus AAV-DIO-shPDGFRβ + Vehicle, p = 0.6523<br>AAV-DIO-shGFP + PDGF-BB versus AAV-DIO-shPDGFRβ + PDGF-BB, p = 0.0013<br>AAV-DIO-shGFP + Vehicle versus AAV-DIO-shGFP + PDGF-BB, p = 0.0170<br>AAV-DIO-shPDGFRβ + Vehicle versus AAV-DIO-shPDGFRβ + PDGF-BB, p = 0.0784	AAV-DIO-shGFP + Vehicle 1827.0 ± 68.9<br>AAV-DIO-shGFP + PDGF-BB 3448.0 ± 541.0<br>AAV-DIO-shPDGFRβ + Vehicle 2375.0 ± 101.8<br>AAV-DIO-shPDGFRβ + PDGF-BB 1198.0 ± 227.7
**7L:** Quantification of the number of BrdU/Sox2/GFAP positive NSCs	Two-way ANOVA	Bonferroni's *post hoc* test	**Mean ± s.e.m.**
AAV-DIO-shGFP + Vehicle (n = 3)<br>AAV-DIO-shGFP + PDGF-BB (n = 4)<br>AAV-DIO-shPDGFRβ + Vehicle (n = 3)<br>AAV-DIO-shPDGFRβ + PDGF-BB (n = 4)	Treatment: $F (1,10) = 1.0040$, $p = 0.3400$<br>Virus: $F (1,10) = 4.9730$, $p = 0.0498$<br>Treatment × Virus: $F (1,10) = 7.8620$, $p = 0.0187$	AAV-DIO-shGFP + Vehicle versus AAV-DIO-shPDGFRβ + Vehicle, p > 0.9999<br>AAV-DIO-shGFP + PDGF-BB versus AAV-DIO-shPDGFRβ + PDGF-BB, p = 0.0065<br>AAV-DIO-shGFP + Vehicle versus AAV-DIO-shGFP + PDGF-BB, p = 0.0453<br>AAV-DIO-shPDGFRβ + Vehicle versus AAV-DIO-shPDGFRβ + PDGF-BB, p = 0.4629	AAV-DIO-shGFP + Vehicle 256.7 ± 32.83<br>AAV-DIO-shGFP + PDGF-BB 533.0 ± 92.3<br>AAV-DIO-shPDGFRβ + Vehicle 298.3 ± 83.7<br>AAV-DIO-shPDGFRβ + PDGF-BB 167.5 ± 50.1
**7M:** Quantification of the number of Sox2/GFAP positive NSCs	Two-way ANOVA	Bonferroni's *post hoc* test	**Mean ± s.e.m.**

AAV-DIO-shGFP + Vehicle (n = 3)<br>AAV-DIO-shGFP + PDGF-BB (n = 4)<br>AAV-DIO-shPDGFRβ + Vehicle (n = 3)<br>AAV-DIO-shPDGFRβ + PDGF-BB (n = 4)	Treatment: F (1,10) = 1.5410, p = 0.2428<br>Virus: F (1,10) = 1.2820, p = 0.2839<br>Treatment × Virus: F (1,10) = 1.5900, p = 0.2359	AAV-DIO-shGFP + Vehicle versus AAV-DIO-shPDGFRβ + Vehicle, p = 0.2145<br>AAV-DIO-shGFP + PDGF-BB versus AAV-DIO-shPDGFRβ + PDGF-BB, p > 0.9999<br>AAV-DIO-shGFP + Vehicle versus AAV-DIO-shGFP + PDGF-BB, p = 0.2890<br>AAV-DIO-shPDGFRβ + Vehicle versus AAV-DIO-shPDGFRβ + PDGF-BB, p > 0.9999	AAV-DIO-shGFP + Vehicle 8067.0 ± 34.8<br>AAV-DIO-shGFP + PDGF-BB 6115.0 ± 539.2<br>AAV-DIO-shPDGFRβ + Vehicle 6200.0 ± 595.3<br>AAV-DIO-shPDGFRβ + PDGF-BB 6215.0 ± 1127.0
**7N:** The ratio of aNSCs to qNSCs<br><br>AAV-DIO-shGFP + Vehicle (n = 3)<br>AAV-DIO-shGFP + PDGF-BB (n = 4)<br>AAV-DIO-shPDGFRβ + Vehicle (n = 3)<br>AAV-DIO-shPDGFRβ + PDGF-BB (n = 4)	Two-way ANOVA<br><br>Treatment: F (1,10) = 1.8670, p = 0.2017<br>Virus: F (1,10) = 3.5670, p = 0.0883<br>Treatment × Virus: F (1,10) = 8.7410, p = 0.0144	Bonferroni's *post hoc* test<br><br>AAV-DIO-shGFP + Vehicle versus AAV-DIO-shPDGFRβ + Vehicle, p = 0.9921<br>AAV-DIO-shGFP + PDGF-BB versus AAV-DIO-shPDGFRβ + PDGF-BB, p = 0.0082<br>AAV-DIO-shGFP + Vehicle versus AAV-DIO-shGFP + PDGF-BB, p = 0.0242<br>AAV-DIO-shPDGFRβ + Vehicle versus AAV-DIO-shPDGFRβ + PDGF-BB, p = 0.5742	**Mean ± s.e.m.**<br><br>AAV-DIO-shGFP + Vehicle 1.00 ± 0.12<br>AAV-DIO-shGFP + PDGF-BB 2.91 ± 0.69<br>AAV-DIO-shPDGFRβ + Vehicle 1.47 ± 0.31<br>AAV-DIO-shPDGFRβ + PDGF-BB 0.77 ± 0.16
**S2C:** Behavioral tests of SIT<br><br>Control (n = 13)	Unpaired *t* test<br><br>t = 11.0700,		**Mean ± s.e.m.**

CSDS (n =12)	p < 0.001		Control 1.884 ± 0.104<br>CSDS 0.502 ± 0.064
**S2D:** Behavioral tests of SPT<br><br>Control (n = 13)<br>CSDS (n =12)	Unpaired *t* test<br><br>t = 6.2040, p < 0.001		**Mean ± s.e.m.**<br><br>Control 87.97 ± 1.58<br>CSDS 52.40 ± 5.73
**S3B:** Behavioral effects of the chemogenetic inhibition of MS-DG neurons in the TST<br><br>Vehicle (n = 14)<br>CNO (n = 17)	Unpaired *t* test<br><br>t = 2.7080, p=0.0112		**Mean ± s.e.m.**<br><br>Vehicle 137.4 ± 7.6<br>CNO 102.5 ± 9.9
**S3C:** Behavioral effects of the chemogenetic inhibition of MS-DG neurons in the FST<br><br>Vehicle (n = 14)<br>CNO (n = 17)	Unpaired *t* test<br><br>t = 2.0960, p = 0.0449		**Mean ± s.e.m.**<br><br>Vehicle 131.1 ± 8.2<br>CNO 106.6 ± 8.2
**S3D:** Behavioral effects of the chemogenetic inhibition of MS-DG neurons in the OFT<br><br>Vehicle (n = 14)<br>CNO (n = 17)	Unpaired *t* test<br><br>t = 0.4048, p = 0.6886		**Mean ± s.e.m.**<br><br>Vehicle 38.69 ± 2.23<br>CNO 39.89 ± 1.96
**S4B:** Patch clamp recording from neurons expressing hM4Di-mCherry in the MS<br><br>Control (n = 4 cells)<br>CNO (n = 4 cells)	Unpaired *t* test<br><br>t = 4.8000, p = 0.0030		**Mean ± s.e.m.**<br><br>Control 9.5 ± 0.6<br>CNO 4.8 ± 0.8
**S4D:** Patch clamp recording from neurons expressing hM3Dq-mCherry in the MS	Two-tailed paired *t* test		**Mean ± s.e.m.**

CNO (n = 4 cells)	t = 14.8100,<br>P = 0.0007		Before-CNO<br>-63.80 ± 4.60<br>After-CNO<br>-40.50 ± 4.23
**S5B:** Behavioral effects of the chemogenetic activation of MS-DG neurons in the SIT<br><br>Vehicle (n = 8)<br>CNO (n = 10)	Unpaired *t* test<br><br>t = 1.1970, p = 0.2486		**Mean ± s.e.m.**<br><br>Vehicle 1.251 ± 0.136<br>CNO 1.688 ± 0.306
**S5C:** Behavioral effects of the chemogenetic activation of MS-DG neurons in the SPT<br><br>Vehicle (n = 8)<br>CNO (n = 11)	Unpaired *t* test<br><br>t = 0.2279, p = 0.8225		**Mean ± s.e.m.**<br><br>Vehicle 71.24 ± 8.09<br>CNO 73.04 ± 3.40
**S5D:** Behavioral effects of the chemogenetic activation of MS-DG neurons in the TST<br><br>Vehicle (n = 8)<br>CNO (n = 11)	Unpaired *t* test<br><br>t = 1.7330, p = 0.1013		**Mean ± s.e.m.**<br><br>Vehicle 143.1 ± 9.5<br>CNO 112.2 ± 13.5
**S5E:** Behavioral effects of the chemogenetic activation of MS-DG neurons in the FST<br><br>Vehicle (n = 8)<br>CNO (n = 11)	Unpaired *t* test<br><br>t = 0.2470, p = 0.8079		**Mean ± s.e.m.**<br><br>Vehicle 101.8 ± 9.0<br>CNO 106.0 ± 13.1
**S5F:** Behavioral effects of the chemogenetic activation of MS-DG neurons in the OFT<br><br>Vehicle (n = 8)<br>CNO (n = 11)	Unpaired *t* test<br><br>t = 0.2452, p = 0.8093		**Mean ± s.e.m.**<br><br>Vehicle 21.71 ± 2.97<br>CNO 22.60 ± 2.22

**S6C:** PDGF-BB mRNA expression in the DG after CSDS<br><br>Control (n = 10)<br>CSDS (n = 13)	Unpaired *t* test<br><br><br>t = 3.0480, p = 0.0061		**Mean ± s.e.m.**<br><br><br>Control 1.000 ± 0.048<br>CSDS 0.759 ± 0.059
**S6D:** PDGF-BB protein levels in the DG as determined by ELISA after CSDS<br><br>Control (n = 8)<br>CSDS (n = 8)	Unpaired *t* test<br><br><br>t = 2.6160, p = 0.0203		**Mean ± s.e.m.**<br><br><br>Control 172.2 ± 6.2<br>CSDS 138.3 ± 11.4
**S6E:** PDGF-BB mRNA expression in the DG after inhibition of MS-DG projection neurons<br><br>Control + Vehicle (n = 10)<br>Control + CNO (n = 10)<br>CSDS + Vehicle (n = 14)<br>CSDS + CNO (n = 16)	Two-way ANOVA<br><br><br>Stress: F (1,46) = 5.3210, p = 0.0256<br>Treatment: F (1,46) = 11.1600, p = 0.0017<br>Stress × Treatment: F (1,46) = 6.5860, p = 0.0136	Bonferroni's *post hoc* test<br><br><br>Control + Vehicle versus CSDS + Vehicle, p = 0.0028<br>Control + CNO versus CSDS + CNO, p > 0.9999<br>Control + Vehicle versus Control + CNO, p > 0.9999<br>CSDS + Vehicle versus CSDS + CNO, p < 0.001	**Mean ± s.e.m.**<br><br><br>Control + Vehicle 0.961 ± 0.088<br>Control + CNO 1.016 ± 0.064<br>CSDS + Vehicle 0.620 ± 0.063<br>CSDS + CNO 1.034 ± 0.062
**S6F:** PDGF-BB mRNA expression in the DG after stimulation MS-DG projection neurons<br><br>Vehicle (n = 13)<br>CNO (n = 14)	Unpaired *t* test<br><br><br>t = 2.5680, p = 0.0166		**Mean ± s.e.m.**<br><br><br>Vehicle 1.000 ± 0.055<br>CNO 0.839 ± 0.032
**S6J:** Behavioral test of TST	Unpaired *t* test		**Mean ± s.e.m.**

Vehicle (n = 14)<br>CNO (n = 16)	t = 3.3810, p = 0.0021		Vehicle 141.9 ± 7.0<br>CNO 100.6 ± 9.6
**S6K:** Behavioral test of FST<br><br>Vehicle (n = 14)<br>CNO (n = 16)	Unpaired *t* test<br><br>t = 3.6550, p = 0.0011		**Mean ± s.e.m.**<br><br>Vehicle 77.8 ± 10.4<br>CNO 34.9 ± 6.1
**S6L:** Behavioral test of OFT<br><br>Vehicle (n = 14)<br>CNO (n = 16)	Unpaired *t* test<br><br>t = 0.6239, p = 0.5377		**Mean ± s.e.m.**<br><br>Vehicle 37.41 ± 2.25<br>CNO 39.94 ± 3.23
**S6M:** PDGF-BB mRNA expression in the DG after MS to DG inhibition in the Vgat-Cre mice<br><br>Vehicle (n = 14)<br>CNO (n = 16)	Unpaired *t* test<br><br>t = 3.1000, p = 0.0044		**Mean ± s.e.m.**<br><br>Vehicle 1.000 ± 0.059<br>CNO 1.425 ± 0.117
**S7B:** PDGF-BB protein levels in the DG as determined by ELISA<br><br>AAV-GFP (n = 7)<br>AAV-PDGF-BB (n = 10)	Unpaired *t* test<br><br>t =4.4140, p = 0.0005		**Mean ± s.e.m.**<br><br>AAV-GFP 164.60 ± 14.73<br>AAV-PDGF-BB 265.60 ± 16.07
**S8B:** Time spent immobile in the FST<br><br>Vehicle (n = 8)<br>0.0072 μg/mL (n = 8)<br>0.072 μg/mL (n = 8)<br>0.72 μg/mL (n = 7)	One-way ANOVA<br><br>Treatment: F (3, 27) = 2.7560, p = 0.0618	Tukey's *post hoc* test<br><br>Vehicle versus 0.0072 μg/mL, p = 0.4412<br>Vehicle versus 0.072 μg/mL, p = 0.6350<br>Vehicle versus 0.72 μg/mL, p = 0.0383<br>0.0072 μg/mL versus 0.072 μg/mL, p = 0.9881<br>0.0072 μg/mL versus 0.72 μg/mL, p = 0.5159	**Mean ± s.e.m.**<br><br>Vehicle 136.1 ± 5.2<br>0.0072 μg/mL 110.3 ± 16.3<br>0.072 μg/mL 115.8 ± 15.0<br>0.72 μg/mL 85.7 ± 7.5

		0.072 µg/mL versus 0.72 µg/mL, p = 0.3422	
**S8C:** Locomotor activity measured by the total distance traveled by the WT mice with vehicle or PDGF-BB treatment<br><br>Vehicle (n = 8)<br>0.0072 µg/mL (n = 8)<br>0.072 µg/mL (n = 8)<br>0.72 µg/mL (n = 7)	One-way ANOVA<br><br>Treatment: F (3, 27) = 1.6110, p = 0.2099	Tukey's *post hoc* test<br><br>Vehicle versus 0.0072 µg/mL, p = 0.4757<br>Vehicle versus 0.072 µg/mL, p = 0.9162<br>Vehicle versus 0.72 µg/mL, p = 0.9996<br>0.0072 µg/mL versus 0.072 µg/mL, p = 0.1775<br>0.0072 µg/mL versus 0.72 µg/mL, p = 0.4470<br>0.072 µg/mL versus 0.72 µg/ML, p = 0.9524	**Mean ± s.e.m.**<br><br>Vehicle 32.60 ± 6.02<br>0.0072 µg/mL 42.10 ± 4.61<br>0.072 µg/mL 28.39 ± 3.81<br>0.72 µg/mL 31.93 ± 3.65
**S9B:** PDGF-BB protein levels in the DG as determined by ELISA<br><br>LV-shGFP (n = 8)<br>LV-shPDGF-BB (n = 8)	Unpaired *t* test<br><br>t = 4.9140, p = 0.0002		**Mean ± s.e.m.**<br><br>LV-shGFP 175.0 ± 15.8<br>LV-shPDGF-BB 90.5 ± 6.7
**S9C:** Behavioral effects of PDGF-BB knockdown in the SIT<br><br>LV-shGFP (n = 6)<br>LV-shPDGF-BB (n = 6)	Unpaired *t* test<br><br>t = 0.8668, p = 0.4064		**Mean ± s.e.m.**<br><br>LV-shGFP 1.252 ± 0.115<br>LV-shPDGF-BB 1.396 ± 0.120
**S9D:** Behavioral effects of PDGF-BB knockdown in the TST<br><br>LV-shGFP (n = 6)<br>LV-shPDGF-BB (n = 6)	Unpaired *t* test<br><br>t = 0.3781, p = 0.7132		**Mean ± s.e.m.**<br><br>LV-shGFP 134.0 ± 11.2

			LV-shPDGF-BB 139.5 ± 9.3
**S9E:** Behavioral effects of PDGF-BB knockdown in the FST<br><br>LV-shGFP (n = 6)<br>LV-shPDGF-BB (n = 6)	Unpaired *t* test<br><br>t = 1.2080, p = 0.2547		**Mean ± s.e.m.**<br><br>LV-shGFP 135.0 ± 8.9<br>LV-shPDGF-BB 122.8 ± 4.7
**S9F:** Behavioral effects of PDGF-BB knockdown in the OFT<br><br>LV-shGFP (n = 6)<br>LV-shPDGF-BB (n = 6)	Unpaired *t* test<br><br>t = 0.09797, p = 0.9239		**Mean ± s.e.m.**<br><br>LV-shGFP 43.76 ± 6.96<br>LV-shPDGF-BB 43.00 ± 3.51
**S10C:** Quantification of the number of BrdU positive cells<br><br>Control + Vehicle (n = 4)<br>Control + PDGF-BB (n = 4)<br>CSDS + Vehicle (n = 3)<br>CSDS + PDGF-BB (n = 4)	Two-way ANOVA<br><br>Stress: F (1,11) = 2.7820, p = 0.1235<br>Treatment: F (1,11) = 45.0200, p < 0.001<br>stress × treatment interaction: F (1,11) = 5.1380, p = 0.0446	Bonferroni's *post hoc* test<br><br>Control + Vehicle versus CSDS + Vehicle, p = 0.0427<br>Control + PDGF-BB versus CSDS + PDGF-BB, p > 0.9999<br>Control + Vehicle versus Control + PDGF-BB, p = 0.0149<br>CSDS + Vehicle versus CSDS + PDGF-BB, p = 0.0002	**Mean ± s.e.m.**<br><br>Control + Vehicle 2334.0 ± 124.7<br>Control + PDGF-BB 3693.0 ± 484.9<br>CSDS + Vehicle 1130.0 ± 15.28<br>CSDS + PDGF-BB 3876.0 ± 256.9
**S10D:** Quantification of the number of BrdU/DCX positive cells<br><br>Control + Vehicle (n = 4)<br>Control + PDGF-BB (n = 4)<br>CSDS + Vehicle (n = 3)	Two-way ANOVA<br><br>Stress: F (1,11) = 4.6700, p = 0.0536<br>Treatment: F (1,11) = 15.4500, p = 0.0024	Bonferroni's *post hoc* test<br><br>Control + Vehicle versus CSDS + Vehicle, p = 0.0474	**Mean ± s.e.m.**<br><br>Control + Vehicle 1489.0 ± 58.5<br>Control + PDGF-BB 2038.0 ± 300.2

CSDS + PDGF-BB (n = 4)	Stress × Treatment: F (1,11) = 2.8500, p = 0.1195	Control + PDGF-BB versus CSDS + PDGF-BB, p > 0.9999<br>Control + Vehicle versus Control + PDGF-BB, p = 0.2543<br>CSDS + Vehicle versus CSDS + PDGF-BB, p = 0.0056	CSDS + Vehicle 546.7 ± 73.1<br>CSDS + PDGF-BB 1923.0 ± 326.7
**S10F:** Quantification of the number of BrdU/Sox2/GFAP positive NSCs	Two-way ANOVA,	Bonferroni's *post hoc* test	**Mean ± s.e.m.**
Control + Vehicle (n = 4)<br>Control + PDGF-BB (n = 4)<br>CSDS + Vehicle (n = 3)<br>CSDS + PDGF-BB (n = 4)	Stress: F (1,11) = 6.1920, p = 0.0301,<br>Treatment: F (1,11) = 27.9000, p =0.0003<br>Stress × Treatment: F (1,11) = 4.2560, p = 0.0635	Control + Vehicle versus CSDS + Vehicle, p = 0.0202<br>Control + PDGF-BB versus CSDS + PDGF-BB, p > 0.9999<br>Control + Vehicle versus Control + PDGF-BB, p = 0.0744<br>CSDS + Vehicle versus CSDS + PDGF-BB, p = 0.0008	Control + Vehicle 335.0 ± 8.7<br>Control + PDGF-BB 467.5.0 ± 52.0<br>CSDS + Vehicle 147.7 ± 16.2<br>CSDS + PDGF-BB 450.0 ± 53.1
**S10G:** Quantification of the number of Sox2/GFAP positive NSCs	Two-way ANOVA	Bonferroni's *post hoc* test	**Mean ± s.e.m.**
Control + Vehicle (n = 4)<br>Control + PDGF-BB (n = 4)<br>CSDS + Vehicle (n = 3)<br>CSDS + PDGF-BB (n = 4)	Stress: F (1,11) = 0.1566, p = 0.6998<br>Treatment: F (1,11) = 0.009234, p = 0.9252<br>Stress × Treatment: F (1,11) = 1.5270, p = 0.2423	Control + Vehicle versus CSDS + Vehicle, p = 0.5800<br>Control + PDGF-BB versus CSDS + PDGF-BB, p > 0.9999<br>Control + Vehicle versus Control + PDGF-BB, p = 0.6962<br>CSDS + Vehicle versus CSDS + PDGF-BB, p = 0.9077	Control + Vehicle 5913.0 ± 168.6<br>Control + PDGF-BB 6345.0 ± 202.1<br>CSDS + Vehicle 6442.0 ± 644.1<br>CSDS + PDGF-BB 6072.0 ± 283.3

**S10H:** The ratio of aNSCs to qNSCs<br><br>Control + Vehicle (n = 4)<br>Control + PDGF-BB (n = 4)<br>CSDS + Vehicle (n = 3)<br>CSDS + PDGF-BB (n = 4)	Two-way ANOVA<br><br>Stress: F (1,11) = 8.1240, p = 0.0158<br>Treatment: F (1,11) = 32.9000, p = 0.0001<br>Stress × Treatment: F (1,11) = 8.1270,<br>p = 0.0158	Bonferroni's *post hoc* test<br><br>Control + Vehicle versus CSDS + Vehicle, p = 0.0051<br>Control + PDGF-BB versus CSDS + PDGF-BB, p > 0.9999<br>Control + Vehicle versus Control + PDGF-BB, p = 0.1145<br>CSDS + Vehicle versus CSDS + PDGF-BB,<br>p = 0.0002	**Mean ± s.e.m.**<br><br>Control + Vehicle 1.00 ± 0.02<br>Control + PDGF-BB 1.30 ± 0.15<br>CSDS + Vehicle 0.40 ± 0.01<br>CSDS + PDGF-BB 1.30 ± 0.12
**S10K:** Quantification of the<br>branch number<br><br>Control + Vehicle (n = 27)<br>Control + PDGF-BB (n = 24)<br>CSDS + Vehicle (n = 26)<br>CSDS + PDGF-BB (n = 24)	Two-way ANOVA<br><br>Stress: F (1,97) = 3.4990, p = 0.0644<br>Treatment: F (1,97) = 19.9200, p < 0.001<br>Stress × Treatment: F (1,97) = 6.8190, p = 0.0105	Bonferroni's *post hoc* test<br><br>Control + Vehicle versus CSDS + Vehicle, p = 0.0032<br>Control + PDGF-BB versus CSDS + PDGF-BB, p > 0.9999<br>Control + Vehicle versus Control + PDGF-BB, p = 0.3830<br>CSDS + Vehicle versus CSDS + PDGF-BB,<br>p < 0.001	**Mean ± s.e.m.**<br><br>Control + Vehicle 4.7 ± 0.3<br>Control + PDGF-BB 5.3 ± 0.4<br>CSDS + Vehicle 3.2 ± 0.3<br>CSDS + PDGF-BB 5.5 ± 0.4
**S10L:** Quantification of the dendritic length<br><br>Control + Vehicle (n = 27)<br>Control + PDGF-BB (n = 24)<br>CSDS + Vehicle (n = 26)<br>CSDS + PDGF-BB (n = 24)	Two-way ANOVA<br><br>Stress: F (1, 97) = 1.1470, p = 0.2869<br>Treatment: F (1,97) = 16.8100, p < 0.001<br>Stress × Treatment: F (1,97) = 18.4500,<br>p < 0.001	Bonferroni's *post hoc* test<br><br>Control + Vehicle versus CSDS + Vehicle, p = 0.0004<br>Control + PDGF-BB versus CSDS + PDGF-BB, p = 0.0567<br>Control + Vehicle versus Control + PDGF-BB,<br>p > 0.9999	**Mean ± s.e.m.**<br><br>Control + Vehicle 635.4 ± 32.4<br>Control + PDGF-BB 628.9 ± 30.1<br>CSDS + Vehicle 455.8 ± 35.9<br>CSDS + PDGF-BB 736.8 ± 34.5

		CSDS + Vehicle versus CSDS + PDGF-BB, $p < 0.001$	
**S11A:** The protein levels of PDGFRβ in the DG after CSDS<br><br>Control (n = 7)<br>CSDS (n = 6)	Unpaired *t* test<br><br><br>$t = 3.3470$, $p = 0.0065$		**Mean ± s.e.m.**<br><br><br>CSDS 1.00 ± 0.06<br>CSDS 0.67 ± 0.08
**S11E:** Quantification of PDGFRβ neurons colocalized with GFP<br><br>AAV-DIO-shGFP (n = 9)<br>AAV-DIO-shPDGFRβ (n = 9)	Unpaired *t* test<br><br><br>$t = 6.7530$, $p < 0.001$		**Mean ± s.e.m.**<br><br><br>AAV-DIO-shGFP 22.510 ± 1.263<br>AAV-DIO shPDGFRβ 12.000 ± 0.910
**S11G:** Quantification of the number of BrdU positive cells<br><br>AAV-DIO-shGFP (n = 4)<br>AAV-DIO-shPDGFRβ (n =4)	Unpaired *t* test<br><br><br>$t = 4.2070$, $p = 0.0056$		**Mean ± s.e.m.**<br><br><br>AAV-DIO-shGFP 2489.0 ± 309.1<br>AAV-DIO-shPDGFβ 1081.0 ± 127.9
**S11H:** Quantification of the number of BrdU/Sox2/GFAP positive NSCs<br><br>AAV-DIO-shGFP (n = 4)<br>AAV-DIO-shPDGFRβ (n =4)	Unpaired *t* test<br><br><br><br>$t = 3.6070$, $p = 0.0113$		**Mean ± s.e.m.**<br><br><br><br>AAV-DIO-shGFP 331.5 ± 35.8<br>AAV-DIO-shPDGFβ 173.3 ± 25.4
**S11I:** The ratio of aNSCs to qNSCs<br><br>AAV-DIO-shGFP (n = 4)<br>AAV-DIO-shPDGFRβ (n =4)	Unpaired *t* test<br><br>$t = 2.4610$, $p = 0.0491$		**Mean ± s.e.m.**<br><br>AAV-DIO-shGFP 1.00 ± 0.21

			AAV-DIO-shPDGFβ 0.45 ± 0.08
**S11J:** Quantification of the number of Sox2/GFAP positive NSCs<br><br>AAV-DIO-shGFP (n = 4)<br>AAV-DIO-shPDGFRβ (n =4)	Unpaired *t* test<br><br><br><br><br>t = 0.5874, p = 0.5783		**Mean ± s.e.m.**<br><br><br><br><br>AAV-DIO-shGFP 6490.0 ± 819.9<br>AAV-DIO-shPDGFβ 7003.0 ± 300.8
**S12F:** The protein levels of JAK2 in the DG after PDGF-BB treatment<br><br>Vehicle (n = 10)<br>PDGF-BB (n = 10)	Unpaired *t* test<br><br><br><br>t = 3.4140, p = 0.0031		**Mean ± s.e.m.**<br><br><br><br>Vehicle 1.00 ± 0.05<br>PDGF-BB 1.38 ± 0.10
**S12G:** The protein levels of STAT3 in the DG after PDGF-BB treatment<br><br>Vehicle (n = 10)<br>PDGF-BB (n = 10)	Unpaired *t* test<br><br><br><br>t = 3.6090, p = 0.0020		**Mean ± s.e.m.**<br><br><br><br>Vehicle 1.00 ± 0.06<br>PDGF-BB 1.30 ± 0.06
**S12I:** The protein levels of JAK2 in the DG after PDGF-BB treatment<br><br>Vehicle (n = 9)<br>PDGF-BB (n = 9)	Unpaired *t* test<br><br><br><br>t = 3.1210, p = 0.0066		**Mean ± s.e.m.**<br><br><br><br>Vehicle 1.00 ± 0.06<br>PDGF-BB 1.52 ± 0.16
**S12J:** The protein levels of STAT3 in the DG after PDGF-BB treatment<br><br>Vehicle (n = 9)<br>PDGF-BB (n = 9)	Unpaired *t* test<br><br><br><br>t = 2.6550, p = 0.0173		**Mean ± s.e.m.**<br><br><br><br>Vehicle 1.00 ± 0.03<br>PDGF-BB 1.18 ± 0.06
